# A complex matrix characterization approach, applied to cigarette smoke, that integrates multiple analytical methods and compound identification strategies for non‐targeted liquid chromatography with high‐resolution mass spectrometry

**DOI:** 10.1002/rcm.8571

**Published:** 2020-01-10

**Authors:** Daniel Arndt, Christian Wachsmuth, Christoph Buchholz, Mark Bentley

**Affiliations:** ^1^ PMI R&D Philip Morris Products S.A. Quai Jeanrenaud 5, CH‐2000 Neuchâtel Switzerland

## Abstract

**Rationale:**

For the characterization of the chemical composition of complex matrices such as tobacco smoke, containing more than 6000 constituents, several analytical approaches have to be combined to increase compound coverage across the chemical space. Furthermore, the identification of unknown molecules requiring the implementation of additional confirmatory tools in the absence of reference standards, such as tandem mass spectrometry spectra comparisons and *in silico* prediction of mass spectra, is a major bottleneck.

**Methods:**

We applied a combination of four chromatographic/ionization techniques (reversed‐phase (RP) – heated electrospray ionization (HESI) in both positive (+) and negative (−) modes, RP – atmospheric pressure chemical ionization (APCI) in positive mode, and hydrophilic interaction liquid chromatography (HILIC) – HESI positive) using a Thermo Q Exactive™ liquid chromatography/high‐resolution accurate mass spectrometry (LC/HRAM‐MS) platform for the analysis of 3R4F‐derived smoke. Compound identification was performed by using mass spectral libraries and *in silico* predicted fragments from multiple integrated databases.

**Results:**

A total of 331 compounds with semi‐quantitative estimates ≥100 ng per cigarette were identified, which were distributed within the known chemical space of tobacco smoke. The integration of multiple LC/HRAM‐MS‐based chromatographic/ionization approaches combined with complementary compound identification strategies was key for maximizing the number of amenable compounds and for strengthening the level of identification confidence. A total of 50 novel compounds were identified as being present in tobacco smoke. In the absence of reference MS^2^ spectra, *in silico* MS^2^ spectra prediction gave a good indication for compound class and was used as an additional confirmatory tool for our integrated non‐targeted screening (NTS) approach.

**Conclusions:**

This study presents a powerful chemical characterization approach that has been successfully applied for the identification of novel compounds in cigarette smoke. We believe that this innovative approach has general applicability and a huge potential benefit for the analysis of any complex matrices.

## 1 INTRODUCTION

High‐resolution accurate mass spectrometry (HRAM‐MS)‐based non‐targeted screening (NTS) is a key methodology for characterizing the chemical composition of complex matrices.^1^ One major part within such a workflow is compound identification that can be achieved by either matching compound features against spectral databases (suspect screening analysis [SSA]) or, without any prior knowledge, by comparing first‐order fragmentation (MS/MS) derived information with *in silico* predicted fragments from compound databases (non‐targeted analysis [NTA]).^2^ NTS enables the simultaneous identification and semi‐quantification of a large number of compounds using an unbiased approach. This approach also allows the performance of accurate mass measurements, tandem experiments to facilitate compound identification (ID), and retrospective targeted screening for compounds of interest.^3^, ^4^ Once interfaced with liquid chromatography (LC), it is also able to achieve isomeric separation of constituents and deliver information regarding the physicochemical properties of compounds.

Given the high number and structural diversity of small molecules in complex matrices such as biological specimens, natural products and tobacco smoke, the latter known to contain more than 6000 constituents,^5^ a combination of analytical approaches is required to cover the broadest possible range of compound classes within these diverse chemical spaces.^6^, ^7^, ^8^ Reversed‐phase (RP) chromatography is a universal separation mode that has been employed most commonly in non‐targeted LC/MS studies,^9^, ^10^ and hydrophilic interaction liquid chromatography (HILIC) has been shown to provide good retention for small and very polar molecules.^11^ In addition to these separation modes, heated electrospray ionization (HESI) and atmospheric pressure chemical ionization (APCI), using both positively (+) and negatively (−) charged ionization, have provided complementary information^12^, ^13^ depending upon analyte polarity, size, and the presence or absence of heteroatoms and functional groups.

For the purposes of establishing a powerful analytical workflow, there is more to consider than simply a requirement for a set of complementary analytical methods that cover the broadest possible chemical space. The integration of each applied method into a standardized and automated data evaluation process, including structural ID with cheminformatics tools, is key for successful handling of the vast amounts of data produced by NTS approaches^14^, ^15^ in a time‐efficient manner. Nevertheless, ID of organic molecules by LC/MS remains a major challenge,^16^, ^17^ with shortcomings including a lack of commercial mass spectral libraries and the unavailability of any standardized retention time (tR) or retention index (RI) systems. However, HRAM‐MS measurements of ionized molecules can be used as a starting point to generate molecular formulae, with consideration for the isotopic pattern^18^ and chemical and heuristic rules.^19^ In order to enhance the degree of confidence in structural candidates, or to achieve *de novo* ID, tandem mass spectral (MS^2^) library searches are of high interest, concomitant with an increasing availability of publicly and commercially available MS^2^ libraries.^3^, ^20^ In the absence of any reference MS^2^ spectra, computational approaches,^21^ including *in silico* fragmentation,^22^, ^23^ have emerged as additional sources of orthogonal information for successful compound ID.

The primary aim of this work was to establish and provide a detailed evaluation of a comprehensive LC/HRAM‐MS‐based NTS strategy for the chemical characterization of tobacco smoke, which combined multiple complementary separation and ionization modes. Evaluation of the output from these complementary analytical approaches was performed using a streamlined semi‐automated data processing and compound ID workflow aimed at improving both specificity and confidence in the annotation of small molecules even in the absence of reference standards, and further enabling the discovery of novel compounds.

## 2 EXPERIMENTAL

### 2.1 Materials

LC/MS‐grade acetonitrile (ACN), formic acid, ammonium fluoride, sodium hydroxide and ammonium acetate were purchased from Sigma‐Aldrich (Basel, Switzerland). LC/MS‐grade methanol (MeOH), isopropyl alcohol, and water were obtained from Honeywell Fluka (Fisher Scientific AG, Switzerland). Stable‐isotope‐labeled [^2^H_19_]decanoic acid and [^2^H_8_]isophorone (2,4,4,6,6‐[^2^H_5_]; 3‐methyl‐[^2^H_3_]) were purchased from C/D/N Isotopes Inc. (Pointe‐Claire, Quebec, Canada) and [2,4,5,6‐^2^H_4_]myosmine from Toronto Research Chemicals (Toronto, Ontario, Canada). The Pierce™ LTQ Velos ESI positive and Pierce™ ESI negative ion calibration solutions were obtained from Thermo Scientific (Fisher Scientific AG, Switzerland). The 3R4F^24^ reference cigarettes were purchased from the University of Kentucky (Kentucky Tobacco Research and Development Center, Lexington, KY, USA).

### 2.2 Sample generation and preparation

**Figure 1:**
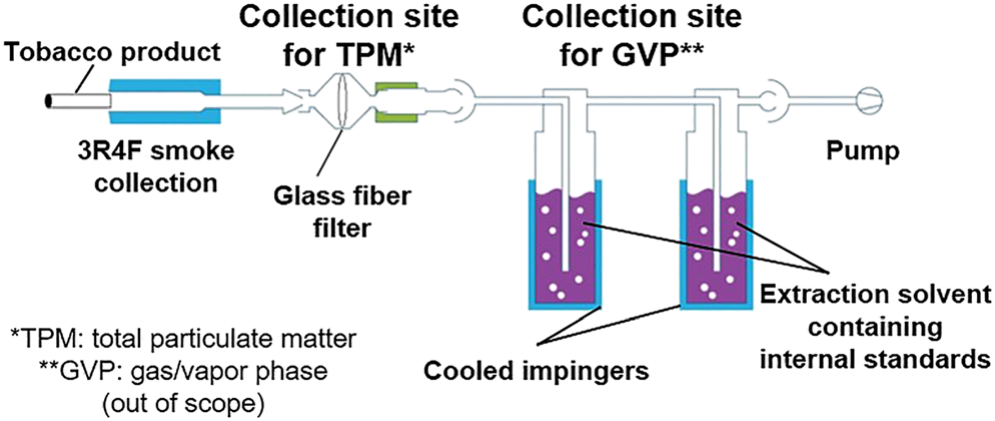
Setup for trapping of 3R4F‐derived whole smoke [Color figure can be viewed at wileyonlinelibrary.com]

Mainstream whole smoke derived from 3R4F cigarettes was generated according to the Health Canada Intense (HC) smoking regime^25^ using a linear smoking machine (puff volume ‐ 55 mL, duration ‐ 2 s, puff interval ‐ 30s). Trapping of the particulate phase (total particulate matter; TPM) was performed using a 44 mm Cambridge glass fiber filter pad (CFP). The gas/vapor phase fraction of whole smoke was trapped using two consecutive microimpingers placed behind the CFP, each filled with 10 mL of extraction solution maintained at approximately −60°C using a dry ice/isopropanol mixture (Figure 1). The total mass of material trapped by the CFP is also referred to as the TPM, which was determined as the weight difference of the CFP before and after the smoke generation process. In this publication we have focused on the analysis of the particulate phase, since the majority of compounds of 3R4F‐derived smoke amenable for analysis are present in this fraction, and the current manuscript is intended to focus on the developed methodology rather than a full characterization of all available smoke fractions. After TPM collection, the filter pad was crushed and extracted using two consecutive steps with either MeOH (2 × 5 mL) or ACN (2 × 5 mL) for RP and HILIC analysis, respectively. The extraction solutions (MeOH and ACN) contained [^2^H_19_]decanoic acid, [^2^H_8_]isophorone, and [2,4,5,6‐^2^H_4_]myosmine as internal standards (ISTD) at 67 μg/mL, 33 μg/mL, and 17 μg/mL, respectively. These internal standard concentrations correspond to amounts of 500 μg per cigarette (μg/cig) for [^2^H_19_]decanoic acid, 250 μg/cig for [^2^H_8_]isophorone, and 125 μg/cig for [2,4,5,6‐^2^H_4_]myosmine in the HPLC vial. Accumulated mainstream smoke from three cigarettes was collected for each sample replicate (*n* = 3). Blank samples were generated with the identical collection setup including the glass filter pad, but without a 3R4F cigarette. Pool samples were prepared as a control in order to represent the entire chemical space of all samples, which comprised equal volumes of smoke extracts and blanks. Prior to analysis, aliquots (300 μL) of the blank samples (*N* = 1 for each extraction), pool samples (N = 1), and extracted TPM sample replicates (*N* = 3) were further diluted with 700 μL MeOH or ACN for RP and HILIC separations, respectively.

### 2.3 Instrumentation

LC/HRAM‐MS analysis using full scan and data‐dependent first‐order fragmentation (MS^2^) modes with high‐energy collision‐induced dissociation (HCD) and stepped normalized collision energy (NCE) was performed using a Q Exactive™ Hybrid Quadrupole Orbitrap mass spectrometer (Thermo Fisher Scientific, Bremen, Germany). The Q Exactive™ was equipped with a choice of either a heated electrospray ionization (H‐ESI II) or APCI source, a quaternary Accela 1250 ultra‐high‐performance liquid chromatography (UHPLC) pump, an Accela AutoOpen autosampler, and a column oven 300 (Thermo Scientific, San Jose, CA, USA). Data were acquired by means of Xcalibur™ software (version 3.1; Thermo Scientific).

Four separate chromatographic/ionization approaches were employed: RP‐LC/HESI(+), RP‐LC/APCI(+), RP‐LC/HESI(–), and HILIC/HESI(+). The column oven and autosampler cooling tray temperatures were set at 50°C and 5°C, respectively. An injection volume of 1.5 μL was used for all injections. Chromatographic separations for RP methods were performed using a Hypersil GOLD™ column (150 × 2.1 mm i.d., 1.9 μm; Thermo Scientific, Waltham, MA, USA) preceded by a UHPLC guard filter cartridge (10 × 2.1 mm i.d., 0.2 μm). For RP positive ionization methods, using 10 mM ammonium acetate in water (eluent A) and 1 mM ammonium acetate in MeOH (eluent B), elution was performed using a 20‐min binary gradient at 400 μL/min: 15–90% B in 7 min, 90–100% B in 5.8 min, 100% B for 5.2 min, back to 15% B in 0.1 min, and 1.9 min equilibration. For RP‐LC/HESI(−), the same gradient was applied, using 1 mM ammonium fluoride in water as eluent A and MeOH as eluent B. Separation of analytes in HILIC mode was achieved using an Accucore™ HILIC column (150 × 2.1 mm i.d., 2.6 μm; Thermo Scientific, Waltham, MA, USA) preceded by a HILIC Defender guard cartridge (10 × 2.1 mm i.d., 2.6 μm). Using 10 mM ammonium acetate in water (eluent A) and 10 mM ammonium acetate in ACN (eluent B), analytes were eluted using a 15‐min binary gradient at 500 μL/min: 98–75% B in 7 min, back to 98% B in 1 min, and 7 min equilibration. A total of five injection replicates (*N* = 5) for each sample were analyzed in each method.

Full‐scan HRAM‐MS was performed over the range 80–800 Da (automatic gain control (AGC) target 3E6, maximum inject time 100 ms) at a resolution of 70,000 (full width at half maximum (FWHM)). For HCD first‐order fragmentation (Top3; loop count, 3; dynamic exclusion, 10 s) a data‐dependent MS^2^ Top3 of each scan at a resolution of 17,500 (FWHM) was used with applied stepped NCEs of 25, 50, and 75 eV (AGC Target 1E5, isolation window 1 Da). For HESI(+) and HESI(−), vaporizer heater temperature, capillary temperature, spray voltage, sheath gas, and auxiliary gas were set at 350°C, 380°C, ± 3.00 kV, 60, and 20 arbitrary units, respectively. APCI(+) settings were as follows: vaporizer heater temperature, 450°C; capillary temperature, 380°C; discharge current, 5.0 μA; sheath gas, 50 arbitrary units; auxiliary gas, 5 arbitrary units. The S‐Lens RF (radio frequency) level was maintained at 55% for all methods. Mass calibration was performed prior to each injection sequence using the H‐ESI II source and the Pierce™ LTQ Velos ESI positive ion calibration mix or the Pierce™ ESI negative ion calibration mix and sodium formate clusters (10mM in water/isopropanol 50:50 v/v) for negative mode. The calibration options provided within the Thermo Scientific software were used.

### 2.4 Data processing

Combined full‐scan and data‐dependent fragmentation data were processed using Progenesis QI™ software (Nonlinear Dynamics, Newcastle upon Tyne, UK), comprising raw data import, alignment, feature extraction, deconvolution, normalization with ISTDs, followed by compound annotation and ID. The pool sample was used as an alignment reference. The following feature extraction settings were used for all runs: sensitivity, automatic; minimum peak width, disabled; retention time limits, disabled; possible adducts, HESI(+): M + H/M + NH_4_, APCI(+): M + H/M + H − H_2_O, HESI(−): M−H/M + F − H. Normalization using a set of housekeeping compounds was performed between individual measurements, which were the same ISTDs as used for semi‐quantification. Manual verification and curation of putatively identified compounds, including the removal of noise artefacts, multiple adducts of the same molecule, and/or in‐source fragmentation products, was performed using Xcalibur™ Qual Browser (version 3.1; Thermo Scientific) software. The structural proposals for each compound in the curated list were further reviewed in Progenesis QI™, and the most likely candidate structure was assigned in consideration of peak abundance, *m/z* (mass‐to‐charge ratio), detected adducts, molecular formula, overall score for mass/tR deviations and isotope similarities, and fragmentation score (FS). A list of the best structural proposals for the extracted compounds was exported as a csv file.

### 2.5 Compound identification

Annotation was performed using a time‐efficient semi‐automatic stepwise SSA/NTA approach by means of matching experimental data with a commercial and an in‐house MS^2^ fragmentation database (SSA) and by matching the experimental fragments against *in silico*‐predicted fragments of compounds from both in‐house and publicly available structure databases (NTA). The fragmentation patterns for all detected compounds in the entire dataset were compared with *in silico*‐predicted fragmentation for compounds present in our in‐house Unique Compounds & Spectra Database (UCSD, PMI, Neuchâtel, Switzerland),^26^ currently comprising 11,392 tobacco‐related compounds, HMDB 4.0 (Human Metabolome Database, University of Alberta, Edmonton, Canada),^27^ and ChemSpider via search plugin with data sources of FDA (U.S. Food and Drug Administration, Silver Spring, MD, USA) and ChemIDplus (ChemIDplus, SIS, NLM, NIH, Bethesda, MD, USA). The MetaScope algorithm search was used within Progenesis QI™ software, with precursor and fragment tolerances of 5 ppm for positive modes and 7 ppm for HESI(−). Queries for elemental composition using FDA and ChemIDplus data sources were limited to a maximum of 100 C, 200 H, 30 O, 10 N, 2 P, and 2 S atoms. In addition, experimental fragmentation spectra for detected constituents were matched against MS^2^ spectra contained within the NIST 14 MS/MS library (U.S. National Institute of Standards and Technology, Gaithersburg, MD, USA), using the same precursor and fragment tolerance settings, and against accurate mass data for ionized molecules, experimental MS^2^ spectra, and tR information contained within the UCSD from the analysis of 460 tobacco‐related reference standards on the same Q Exactive™ MS platform. A tR tolerance of 0.7 min was set. All putative hits were ranked using Progenesis QI™ algorithms, which considered accurate mass similarity, tR similarity, isotope similarity, and FS. In a nutshell, feature comparison for the entire data set versus 233,744 MS/MS spectra (combining UCSD and NIST 14 MS/MS) for suspect screening, and versus >500,000 *in silico*‐predicted fragmentation patterns (for compounds contained within UCSD, HMDB 4.0, FDA and ChemIDplus) for non‐targeted analysis, was achieved within approximately 3 h.

### 2.6 Semi‐quantification

Excel was used for the calculation of semi‐quantitative levels and relative standard deviations (RSDs), which were based upon normalized peak volume ratios expressed as the sum of ion abundances within the isotope boundaries. The semi‐quantified concentration for each compound was estimated by comparison with an ISTD of known concentration, which was chosen depending upon the ionization mode used: ([^2^H_8_]isophorone for RP‐LC positive ionization methods, [^2^H_19_]decanoic acid for RP‐LC/HESI(−), and [2,4,5,6‐^2^H_4_]myosmine for HILIC/HESI(+)). Recognizing that ionization efficiencies may vary greatly between compounds, this simplified approach was estimated to be sufficient for determining compound yields to within an order of magnitude from actual.

### 2.7 Calculation of VP and logP_OW_ values

ACD/Labs Percepta Batch (version 2016.1.1; Advanced Chemistry Development, Inc., Toronto, ON, Canada) was used for calculation of vapor pressure (VP, in mmHg, at 25°C) and logP_octanol/water_ (logP_OW_) values for all compounds registered in UCSD, to create a two‐dimensional (2D) representation of the known chemical space for tobacco smoke 5 including compounds not amenable for classical small molecule mass spectrometric techniques (e.g. metals, peptides and proteins). In addition, VP and logP_OW_ values were predicted for 50 newly identified smoke constituents that were not present in UCSD. Calculations were based on molecular structures. LogP_OW_ is the logarithm of the concentration ratio of un‐ionized solute partitioned between octanol and water.

## 3 RESULTS AND DISCUSSION

### 3.1 Analytical approaches

**Figure 2:**
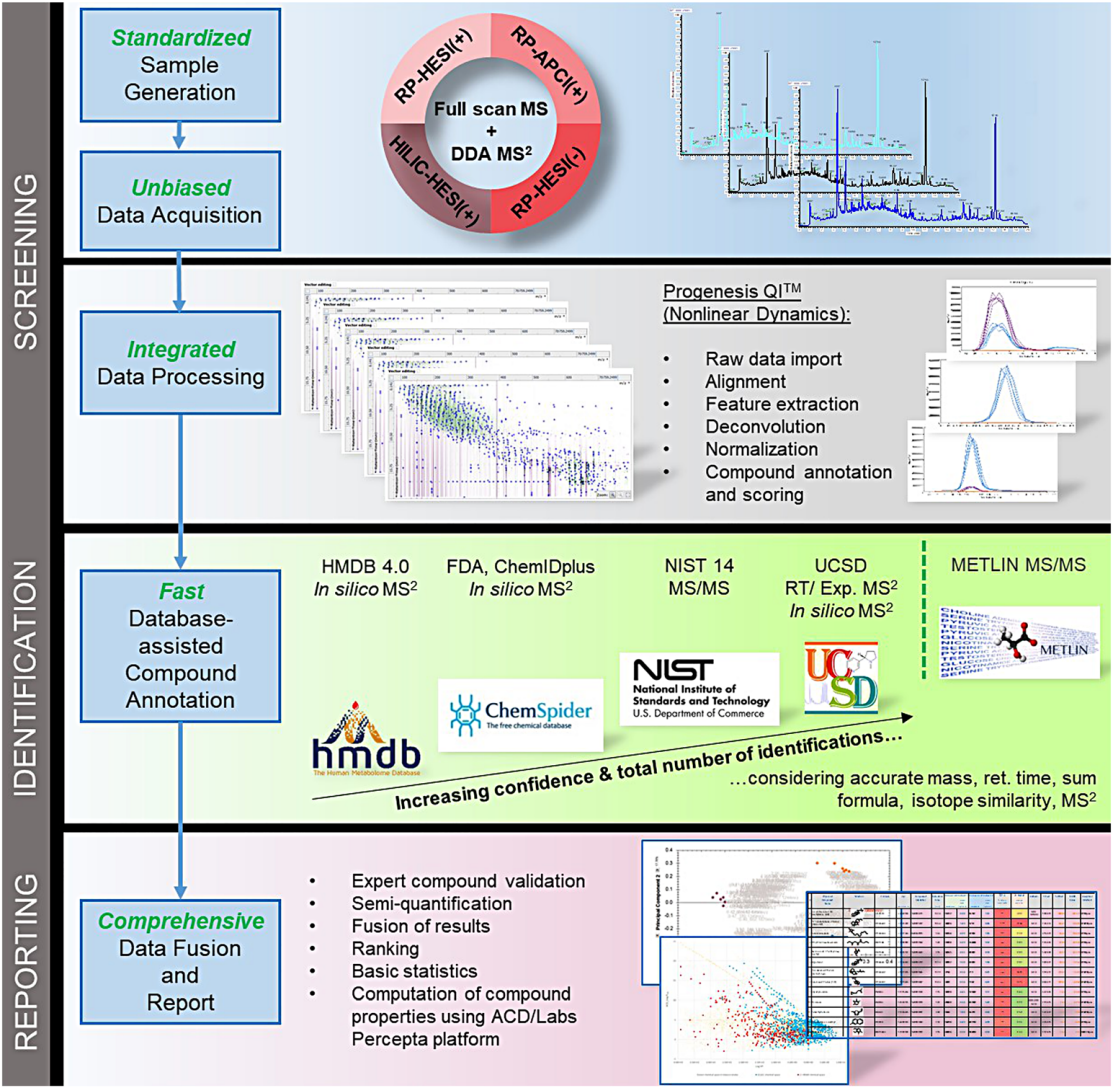
Workflow for LC/HRAM‐MS‐based NTS. DDA, data‐dependent acquisition; UCSD, Unique Compounds & Spectra Database (tobacco‐specific in‐house database) [Color figure can be viewed at wileyonlinelibrary.com]

The methodology was designed as a generic, unbiased approach for the comprehensive chemical characterization of different kinds of matrices (i.e., without any predefined target analytes). Considering the tremendous diversity in structural and chemical properties of small molecules, four separate chromatographic/ionization approaches (RP‐LC/HESI(+), RP‐LC/APCI(+), RP‐LC/HESI(–), HILIC/HESI(+)) for mass spectrometric analysis in full‐scan combined with MS^2^ data acquisition modes have been developed and integrated into a NTS workflow (Figure 2). Each of these four approaches was individually optimized to maximize chemical coverage and sensitivity. For instance, ammonium fluoride was added to the mobile phase to enhance ionization efficiency in the negative ionization mode, a recommendation that has been reported in the literature for global metabolite profiling.^28^

**Figure 3:**
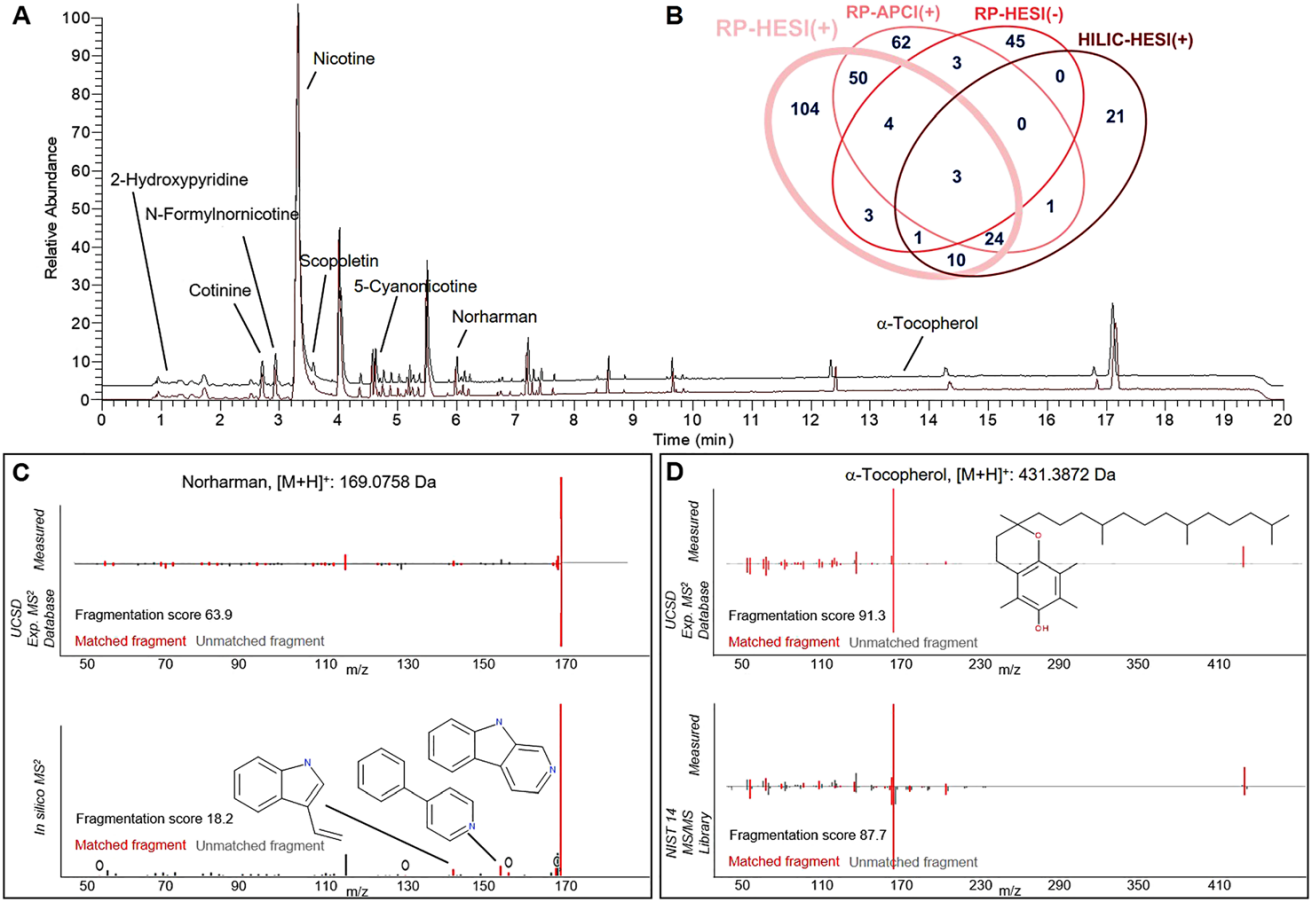
Chromatographic separation and compound ID using LC/HRAM‐MS‐based NTS. A, Overlaid BPCs from two injection replicates of a methanolic TPM extract of tobacco smoke acquired in RP‐LC/HESI(+) mode at the beginning and end of the analytical sequence. B, Coverage and overlap of compounds identified by the four separate chromatographic/ionization approaches. C, RP‐HESI(+) mass spectra for norharman and D, α‐tocopherol are given as examples for the outcome of the analysis performed using complementary ID methods. Further annotated analytes besides nicotine are discussed in the main text [Color figure can be viewed at wileyonlinelibrary.com]

RP‐LC/HESI(+) achieved the greatest coverage with 199 identified compounds, 104 of which were unique to that method. RP‐LC/HESI(+)‐HRAM‐MS base peak chromatograms (BPC) from two injection replicates of a methanolic TPM extract, acquired at the beginning and end of the analytical sequence, are overlaid in Figure 3A. The BPCs demonstrate excellent tR stability, which indicates the robustness of the chromatographic system and is key for proper alignment of the dataset and the confirmation of putative annotations with reference standards in the absence of RI markers. The Venn diagram presented in Figure 3B and the full list of 3R4F‐derived particulate phase smoke constituents presented in Table S1 (supporting information) clearly demonstrate the complementary characteristics of all applied analytical methods which contributed, in varying degrees, to the identification of 331 major constituents. Among the other chromatographic/ionization approaches, a total of 147 compounds were identified using RP‐APCI(+), comprising 62 method unique compounds and 81 compounds overlapping with RP‐HESI(+). Given that the same analytical column and solvents were used for RP methods with positive ionization, these numbers indicate both a high degree of complementarity and also many differences due to the varying susceptibility for matrix effects using either HESI(+) or APCI(+) ionization mechanisms, as has been reported previously.^29^, ^30^ Such an overlap between RP‐LC/HESI(+) and RP‐LC/APCI(+) was desirable in order to minimize the possibility for analytical gaps in the chemical space amenable to LC/HRAM‐MS. Using the RP‐LC/HESI(−) approach, a further 45 “unique to method” compounds were identified, in particular molecules containing carboxyl groups. Finally, HILIC/HESI(+) provided additional information for 21 polar compounds of low molecular weight.^6^, ^11^ Similar to the results presented here, several other published reports have shown an overall higher capacity for metabolic coverage in biological matrices when using a combination of RP‐LC, HILIC, aqueous normal phase, or perfluoro phase separations,^6^, ^11^, ^31^ leading to enhanced resolution of isomers and a concomitant detection of novel metabolites in human serum^6^ and urine.^11^, ^31^ The benefit of HILIC/HESI(+) for the analysis of polar compounds of low molecular weight, in particular for the TPM of 3R4F‐derived smoke, can be seen in the plot of logP_OW_ versus molecular weight for compounds identified by all four separate chromatographic/ionization approaches (Figure S1, supporting information). Of the 38 compounds that were measurable by both RP‐LC/HESI(+) and HILIC/HESI(+), 28 compounds eluted later on the RP column, and 10 were better retained by the HILIC/HESI(+) methodology.

### 3.2 Compound identification strategy

Integration of these analytical approaches with a semi‐automated stepwise data processing workflow was achieved using Progenesis QI™ software, querying fragmentation information from multiple sources/databases, which has been shown to be preferable over using fewer or single resources.^20^ High‐quality experimental MS^2^ mass spectra were obtained by applying both HCD and collision‐induced dissociation ion activation modes, which, amongst other MS parameters, can critically affect the matching of mass spectra.^3^ Parallel usage of both ion activation modes was found to be a complementary approach that increased compound ID rates.^32^, ^33^ A modified MetFrag *in silico* fragmentation algorithm,^23^, ^34^ implemented in Progenesis QI™, was used to perform *in silico* fragmentation of putative candidates retrieved from structure databases for each detected compound in the dataset, which were then compared with the experimentally determined fragmentation pattern. Each of the *in silico*‐predicted fragments were compared with the respective *m/z* trace for the experimental fragmentation spectrum and annotated with the assigned substructure. For the comparison of experimental versus database‐derived fragmentation, an algorithm based on the cosine similarity method^35^, ^36^ was employed in Progenesis QI™. As illustrated by the examples for norharman (87.7 μg/cig) and α‐tocopherol (33.6 μg/cig) presented in Figures 3C and 3D, respectively, *in silico* prediction for MS^2^ spectra, in addition to NIST MS/MS library and experimental MS^2^ fragmentation database comparisons, were applied successfully. FS ranged between 0 and 100. A FS > 45 was found to be strongly indicative for a putatively annotated compound, whereas a lower FS did not necessarily indicate a false candidate; however further confirmation was needed. Finally, FS were dependent upon the degree of fragmentation for individual compounds. For instance, major fragments of norharman were correctly assigned to substructures by the *in silico* fragmentation algorithm (Figure 3C, lower panel); however, several signals present in the experimental spectrum remained unexplained, potentially from in‐source fragmentation or side reactions, ultimately leading to a distinctly lower *in silico* FS of 18.2 versus UCSD database FS of 63.9 (Figure 3C, upper panel), because the latter database incorporated these aspects as it was experimentally determined. Both norharman and α‐tocopherol were subsequently confirmed by a reference standard. However, final confirmation is often not possible due to the unavailability of reference standards, greatly increasing the importance of computational approaches, including *in silico* fragmentation,^21^ if one considers the increasing numbers of unknowns revealed by rapid progress in MS instrumentation and front‐end separation technologies for a wide range of compound classes.

**Figure 4:**
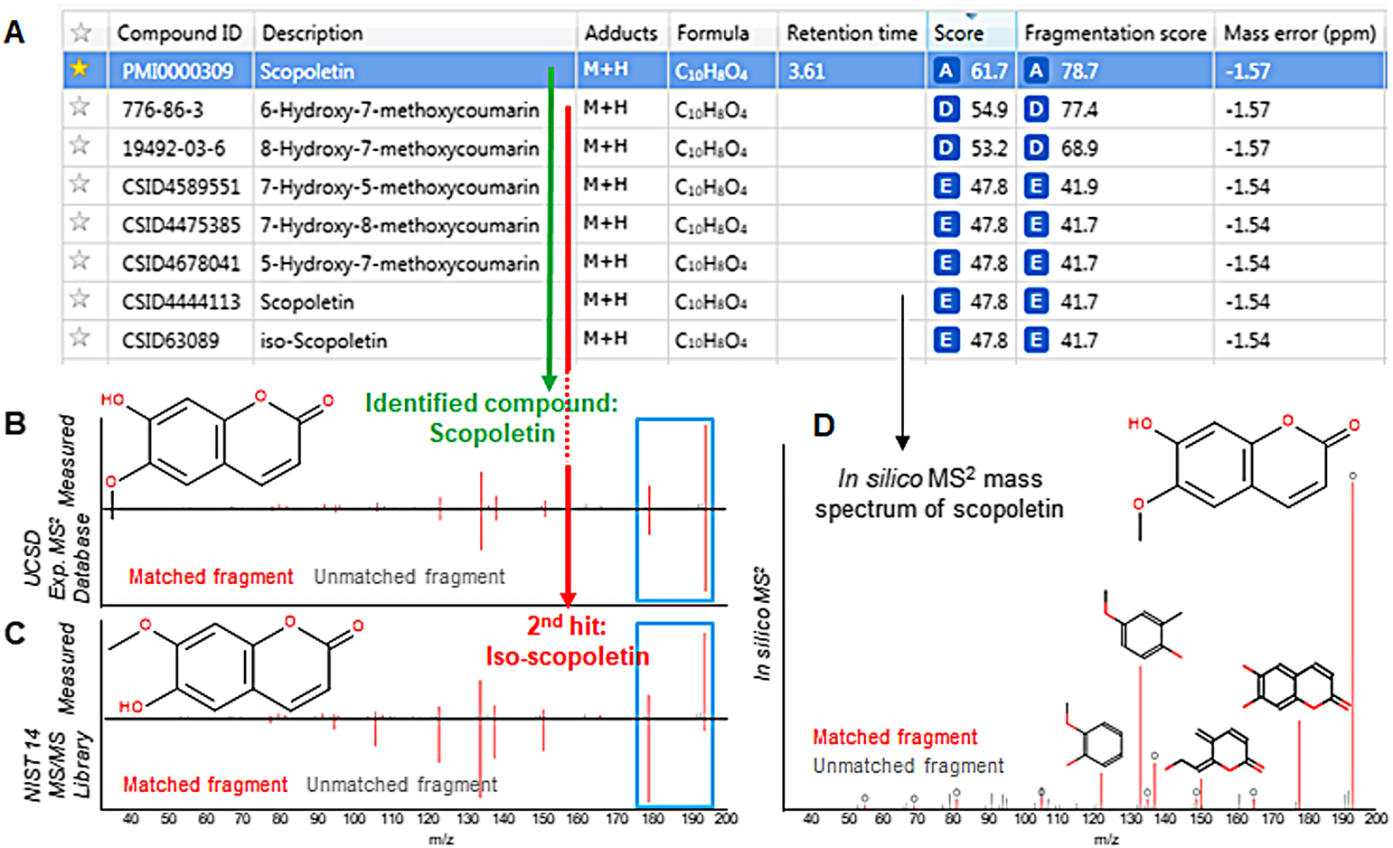
Differentiation of structural isomers for scopoletin in tobacco smoke using LC/HRAM‐MS and an experimental MS^2^ fragmentation database [Color figure can be viewed at wileyonlinelibrary.com]

A limitation of *in silico* fragmentation for the differentiation of structural isomers is demonstrated for scopoletin (7‐hydroxy‐6‐methoxycoumarin, see Figure 4) as an example, which was confirmed by reference standard. Nevertheless, our overall workflow of combined ID strategies enabled differentiation between structural isomers based upon the recorded first‐order fragmentation spectrum, providing that the fragmentation pattern was different. As shown in Figure 4A, a single feature (RP tR: 3.61 min, *m/z*: 193.0492 Da ([M + H]^+^)) had been initially assigned to several very similar molecules by Progenesis QI™. Scopoletin (Figure 4B) was identified as the top hit based upon a match with our in‐house experimental MS^2^ fragmentation database, whereas 6‐hydroxy‐7‐methoxycoumarin (Figure 4C), which is synonymous with iso‐scopoletin and is a configurational isomer of scopoletin, was ranked second highest overall by comparison with the NIST 14 MS/MS library. In addition to the experimental MS^2^ fragmentation database match (top hit), scopoletin was also proposed by comparison with *in silico*‐predicted MS^2^ spectra as the seventh hit (CSID4444113, Figure 4D). The assigned fragments matched the structural features for scopoletin with a good fit (FS = 41.7). However, higher‐ranked hits that corresponded to other hydroxymethoxycoumarin isomers were found, including those proposed by the *in silico* approach (CSID4589551, CSID4475385, CSID4678041, Figure 4), all of which exhibited near identical fragmentation and could not be distinguished based on scoring. In a second example, shown in Figure S2 (supporting information), cotinine (C10H12N2O) could be distinguished from *N*‐formylnornicotine (C10H12N2O) due to the higher spectral match score with the first‐order fragmentation spectrum of cotinine comprised in UCSD. In both examples, tR information strengthened the confidence for correct annotation of the isomeric compounds. The two pairs were baseline‐separated in RP mode due to the chromatographic resolution achieved by sub‐2‐μm particle packed columns, which in this study also contributed to the successful ID of other isomeric pairs/groups that had identical or similar MS^2^ spectra.

### 3.3 Chemical constituents of 3R4F TPM

As a proof of principle, LC/HRAM‐MS‐based non‐targeted screening was used for the chemical characterization of the particulate phase of 3R4F‐derived smoke. A subset comprising the top 25 major chemical constituents is presented in Table 1. In addition, the full list of 331 identified compounds is shown in Table S1 (supporting information). Details for the name and identifier, CAS number, empirical formula, semi‐quantitative yield, monoisotopic masses for the measured *m/z* values, tR, RSD, logVP and logP_OW_ values, analytical method, and key parameters for compound ID (scores, mass and tR deviations, ID strategy) are shown in the same table. Compounds are presented in order of their semi‐quantitative estimates in the samples (yields in [μg/cig]) (Table S1, supporting information). Identified compounds were semi‐quantified with concentrations from 6762 μg/cig (solanesol) down to 0.19 μg/cig (2(3*H*)‐furanone, dihydro‐5‐(1‐hydroxyethyl)), spanning a range between 27‐fold higher, down to one‐thousandth of the concentration of their corresponding internal standard, [^2^H_8_]isophorone. The semi‐quantitative levels should be considered as estimates to within an order of magnitude from actual, since calculations were based on a single ISTD per method. A more accurate semi‐quantification approach, for example, based on individual ISTDs for different compound classes, is desirable, which is planned to be developed in the future.

**Table 1:** Top 25 most abundant chemical constituents identified in 3R4F‐derived smoke by LC/HRAM‐MS NTS^a^

#	Name^b^	Identifier	Formula	*m/z* expt^c^	tR(min)	RSD^d^ (%)	Overall score	FS	Δm^e^ (ppm)	Isotope similarity	ΔtR^f,g^(min)	Method^h^	ID basis
1	**Solanesol**	PMI0000409	C45H74O	648.60630	17.08	2.7	71.0	91.3	−2.4	99.8	0.0	**RP‐LC/HESI(+)**, RP‐LC/APCI(+)	UCSD MS^2^
2	**Nicotine**	PMI0004286	C10H14N2	163.12266	3.32	2.9	59.3	98.9	−1.9	89.7	−0.1	**RP‐LC/HESI(+)**, RP‐LC/APCI(+), HILIC/HESI(+)	UCSD MS^2^
3	**Bombiprenone**	PMI0006795	C43H70O	620.57552	16.76	3.3	42.7	15.3	−1.0	99.4	n/a	**RP‐LC/HESI(+)**, RP‐LC/APCI(+)	UCSD *in silico* MS^2^
4	**Triacetin**	PMI0000113	C9H14O6	236.11230	4.03	5.2	76.0	100.0	−2.2	99.3	0.0	RP‐LC/HESI(+)	UCSD MS^2^
5	**7‐Ketositosterol**	PMI0009304	C29H48O2	429.37223	12.30	7.3	44.9	30.3	−1.1	95.5	n/a	**RP‐LC/HESI(+)**, RP‐LC/APCI(+), HILIC/HESI(+)	UCSD *in silico* MS^2^
6	**Pytoene carotenoid**	HMDB39093	C40H64	545.50760	15.51	4.2	41.8	11.7	−0.9	98.5	n/a	RP‐LC/HESI(+)	HMDB *in silico* MS^2^
7	**5,9,13,17,21,25,29‐Hentriacontaheptaen‐2‐one, 6,10,14,18,22,26,30‐heptamethyl**	PMI0006129	C38H62O	552.51270	15.19	3.7	46.0	33.7	−0.9	97.5	n/a	RP‐LC/HESI(+)	UCSD *in silico* MS^2^
8	**(3β)‐3‐Methylandrost‐5‐en‐17‐one**	CSID114234	C20H30O	287.23637	8.39	3.1	50.4	56.9	−2.0	97.4	n/a	RP‐LC/HESI(+)	ChemIDplus *in silico* MS^2^
9	**Palmitic acid**	PMI0000164	C16H32O2	255.23332	9.78	4.0	56.6	59.2	1.4	98.1	0.1	RP‐LC/HESI(−)	UCSD MS^2^
10	**N‐Octanoylnornicotine**	PMI0001863	C17H26N2O	275.21140	7.21	4.4	62.8	58.2	−1.4	97.5	0.0	**RP‐LC/HESI(+)**, RP‐LC/APCI(+), HILIC/HESI(+)	UCSD MS^2^
11	**Solanochromene**	PMI0008547	C53H80O2	766.64837	18.77	4.1	48.6	49.3	−2.1	96.5	n/a	**RP‐LC/HESI(+)**, RP‐LC/APCI(+)	UCSD *in silico* MS^2^
12	**Linolenic acid**	PMI0000169	C18H30O2	277.21765	9.11	4.4	48.8	27.5	1.2	97.8	0.1	RP‐LC/HESI(+), **RP‐LC/HESI(−)**	UCSD MS^2^
13	**Scopoletin**	PMI0000309	C10H8O4	193.04923	3.61	1.5	61.7	78.7	−1.6	99.2	0.0	**RP‐LC/HESI(+)**, RP‐LC/APCI(+), RP‐HESI(−)	UCSD MS^2^
14	**2,7,12‐Cyclotetradecatrien‐1‐ol, 1,7‐dimethyl‐11‐methylene‐4‐(1‐methylethyl)‐**	PMI0008333	C20H32O	289.25194	8.58	3.2	56.7	88.9	−2.3	97.5	n/a	**RP‐LC/HESI(+)**, RP‐LC/APCI(+)	UCSD *in silico* MS^2^
15	**Linolic acid**	PMI0000168	C18H32O2	279.23333	9.50	4.4	57.2	64.0	1.3	97.7	0.1	RP‐LC/HESI(−)	UCSD MS^2^
16	***N*‐Formylnornicotine**	PMI0006520	C10H12N2O	177.10195	2.94	3.8	64.5	61.1	−1.6	97.9	0.0	**RP‐LC/HESI(+)**, RP‐LC/APCI(+), HILIC/HESI(+)	UCSD MS^2^
17	**(all‐E)‐6,10,14,18,22,26‐hexamethyl‐5,9,13,17,21,25‐Heptacosahexaen‐2‐one**	PMI0006786	C33H54O	484.45070	13.81	4.7	44.8	28.1	−0.9	97.2	n/a	RP‐LC/HESI(+)	UCSD *in silico* MS^2^
18	**α‐Levantenolide**	PMI0008002	C20H30O3	319.22598	7.74	2.4	41.1	11.9	−2.5	96.7	n/a	RP‐LC/HESI(+)	UCSD *in silico* MS^2^
19	**Solanesyl acetate**	PMI0008537	C47H76O2	690.61729	18.75	3.5	39.7	9.9	−1.6	90.6	n/a	RP‐LC/HESI(+)	UCSD *in silico* MS^2^
20	**1,3,5,7,11‐Cembrapentaene, (1E,3Z,5E,7Z,11E)**	PMI0009274	C20H30	271.24145	9.86	3.1	52.0	64.6	−2.1	97.8	n/a	**RP‐LC/HESI(+)**, RP‐LC/APCI(+)	UCSD *in silico* MS^2^
21	**14,15‐Dinor‐8‐labdene‐7,13‐dione**	PMI0006630	C18H28O2	277.21576	7.59	2.0	42.4	16.1	−1.6	97.8	n/a	RP‐LC/HESI(+)	UCSD *in silico* MS^2^
22	**Cotinine**	PMI0001948	C10H12N2O	177.10197	2.73	3.1	53.5	69.4	−1.6	89.7	−0.1	**RP‐LC/HESI(+)**, RP‐LC/APCI(+), HILIC/HESI(+)	UCSD MS^2^
23	**Stearic acid**	PMI0000166	C18H36O2	283.26469	10.58	2.3	60.6	77.6	1.5	97.7	0.1	RP‐LC/HESI(−)	UCSD MS^2^
24	**Piperidine, 1‐(3‐pyridinemethyl)‐2‐cyano‐4,5‐didehydro**	PMI0007755	C12H13N3	200.11786	4.58	4.0	43.6	33.0	−1.8	87.3	n/a	**RP‐LC/HESI(+)**, RP‐LC/APCI(+), HILIC/HESI(+)	UCSD *in silico* MS^2^
25	**β‐Levantenolide**	PMI0008043	C20H30O3	319.22634	6.85	3.0	41.5	11.9	−1.4	97.3	n/a	RP‐LC/HESI(+)	UCSD *in silico* MS^2^

a ^a^Compounds are sorted in descending order of yield. A full list of identified compounds including CAS number, yield, logVP, and logP_OW_ values is provided in Table S1 (supporting information). ^b^ Confidence levels: dark green, confirmed: tR and mass spectra were within specified tolerance ranges in comparison to a standard under the same experimental conditions; light green, high: overall score > 50 or overall score > 45 and FS > 45; yellow, medium: overall score < 45 or overall score between 45 and 50 and FS < 45. ^c^ *m/z* quantifier ion from method specified in “method” column. ^d^ RSD, relative standard deviation (*N* = 15 total observations from three sample replicates that were injected fivefold; calculation based upon normalized peak volume ratios from Progenesis QI™). ^e^ Δm, difference between experimental and theoretical mass. ^f^ ΔtR, difference between tR in a sample and standard in database. ^g^ n/a, not available. ^h^ Method highlighted in bold: method from which the information on the analytical figures were extracted, for cases where compounds were identified with multiple analytical methods.

For the purpose of determining the reliability of our workflow, including sample generation and preparation as well as data acquisition and processing, a total of *N* = 15 observations were made, derived from three sample replicates and five injections per replicate. RSD values for identified compounds ranged between 1% and 12%, demonstrating good analytical performance as well as reliable compound extraction, alignment, and integration achieved by Progenesis QI™.

The overall ID score was calculated between 0 and 80 as a combination of FS, tR, accurate mass match and isotopic similarity, with each parameter equally weighted.^37^ Higher scores up to 100 could not be achieved with our approach due to the absence of collision cross section information from ion mobility experiments, which were not performed. An isotope pattern filter has proven beneficial to further reduce the number of structural proposals for a given empirical formula in cases where high mass accuracy alone was insufficient for compound ID when querying elemental composition.^18^, ^19^, ^38^, ^39^ For evaluating whether isotope similarity values were negatively impacted by low signal intensity, as has already been reported in the literature,^38^, ^39^ compounds were grouped according to isotope similarity (>95 and < 95) and their distribution over the concentration range was evaluated (see Figure S3, supporting information). No clear relationship could be established between low isotope similarity and low concentration, with the majority of compounds of isotope similarity <95 found to be in the 4–40 μg/cig intermediate range. This included 5‐methoxytryptophan (57.7) and acetamide, *N*‐(2‐phenylethyl) (63.2), which exhibited the lowest isotope similarity values for the entire dataset due to coeluting matrix compounds, and to which only medium probability for identification was assigned. Nevertheless, for 71% and 89% of all compounds, isotope similarity values were considered acceptable between the ranges 95–100 and 90–95, respectively. In addition, highly consistent values for isotope similarity were obtained, as shown in Figure S4 (supporting information), which presents base peak and extracted ion chromatograms for two compounds of low concentration analyzed by each of the four analytical methods.

**Figure 5:**
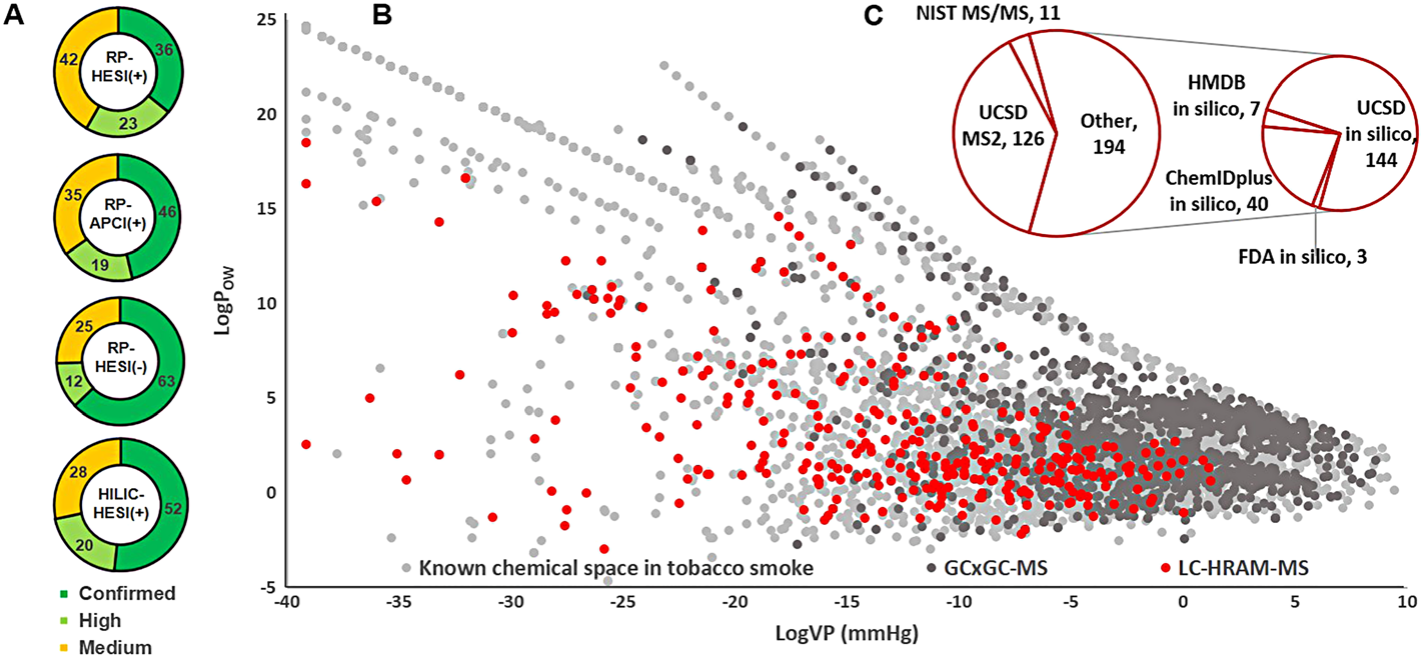
Identified chemical components in TPM derived from a 3R4F reference cigarette. A, Relative distribution of confidence levels for identification of compounds using the four analytical methods. Confidence levels are specified in Table 1 (footnote ^b^). B, Distribution of compounds identified by LC/HRAM‐MS‐based NTS and a complementary GC × GC/TOFMS‐based platform analysis performed in our laboratories^42^ within the known chemical space of tobacco smoke. C, Total number of compounds identified by the different ID strategies [Color figure can be viewed at wileyonlinelibrary.com]

Confirmed proposals resulting in the highest degree of confidence (i.e., level 1 IDs as proposed by Sumner et al^40^ and later refined by Schymanski et al^41^) were validated by comparison of tR and mass spectra for analytical reference standards analyzed under the same experimental conditions (*N* = 126 in total, ~38% confirmed). Confidence for compound ID was assigned as “high” if the overall score was above 50 or between 45 and 50 in combination with a FS above 45, which indicated either correct compound ID or a similar structural isomer. Scores not matching these criteria were classified as “medium” confidence IDs, which typically points to at least the correct compound class. Figure 5A depicts the relative distributions of confirmed, high, and medium IDs for the four different analytical methods, which were independent of compound concentration as shown in the left panel of Figure S3 (supporting information). Details for the individual compounds are indicated by color code in Table 1 and Table S1 (supporting information). Overall scores for compounds reported ranged between 35 and 76. RP‐LC/HESI(+) represented the highest proportion of unconfirmed compounds (>60%), with two‐thirds having only medium‐confidence IDs. A proportion of these putatively identified compounds have not been reported as being present in tobacco and/or tobacco smoke by Rodgman and Perfetti^5^ and were therefore not present in UCSD. In addition, a large number of reactive intermediates (e.g., carotenoid degradation products) unique to the RP‐LC/HESI(+) method, for which reference standards were not commercially available, contributed to the high proportion of unconfirmed compounds. In contrast, more than 60% of compounds identified using RP‐LC/HESI(−) were confirmed by reference standard, with the remaining compounds being identified with either medium (25%) or high confidence (12%). The FS values for RP‐LC/HESI(−) compounds were noticeably lower than that measured with all other methods (median FS = 36 versus 44, P = 0.4, Student's t test), due to the lower degree of fragmentation and a concomitant lower proportion of explained fragments observed in negative ionization mode. It is clear from these findings that additional confirmatory tools are required to complement existing techniques,^16^ thereby increasing the accuracy for structural elucidation of small molecules.

The confidence for compound identification in the TPM of 3R4F‐derived smoke increased noticeably using the following integrated sequence of compound identification strategies: UCSD experimental MS^2^ comparison *>* NIST MS/MS comparison > *in silico* prediction of MS^2^ spectra comparison. Applying these strategies sequentially, it was possible to assess the reliability/strength of each approach on a compound‐by‐compound basis. The most reliable ID strategies for individual compounds are listed in Table 1 and Table S1 (supporting information) (“ID Basis”). *In silico* prediction of MS^2^ spectra gave a good indication for compound class, thereby narrowing the chemical space in which to search for individual compounds, with limitations for distinction between structural isomers (as demonstrated by the example of scopoletin in the previous section). These limitations were mitigated by the application of UCSD experimental MS^2^ and NIST MS/MS comparisons, which were ranked based upon FS. Evaluation of a small set of 16 compounds that were identified using both UCSD MS^2^ and NIST MS/MS sources demonstrated that FS using the UCSD experimental MS^2^ library were significantly higher than for NIST MS/MS library comparisons (median FS 64.0 and 21.1 respectively; P = 0.004, Student's t test; see scores listed in Table S2 (supporting information)), because only UCSD mass spectra incorporate all instrument‐ and method‐specific compound characteristics. However, commercially or publicly available MS/MS libraries have proven beneficial for compound annotation in cases of limited availability of authentic standards.^43^

### 3.4 Analytical coverage of the NTS workflow

Natural products such as tobacco contain a high number of diverse small molecules with a wide range of concentrations. In order to assess the coverage of our LC/HRAM‐MS‐based non‐targeted workflow, the 331 major constituents identified as being present in the particulate phase of 3R4F smoke were compared with the known chemical space of tobacco smoke. Predicted VP and logP_OW_ values for 4141 compounds (from UCSD) known to be present in tobacco smoke were plotted as logP_OW_ versus logVP to create a 2D representation of the chemical space for tobacco smoke. Figure 5B shows the distribution of chemical constituents measured by LC/HRAM‐MS (red dots) within the chemical space comprising these 4141 tobacco and/or smoke constituents (light grey dots). The 331 compounds identified by the four LC/HRAM‐MS approaches were distributed over a considerable proportion of the chemical space, with a particular strength observed for semi‐volatile and nonvolatile compounds over a wide range of polarities up to logP_OW_ >15. Low‐polarity compounds that were not covered, in particular long‐chain wax esters, either exceeded the acquisition mass range of 80–800 Da or were not amenable to the extraction conditions employed. Extraction with MeOH/ACN, as performed for our NTS workflow, was not optimal for this class of compounds and would require the use of more lipophilic solvents.^44^ In combination with complementary NTS approaches based on comprehensive two‐dimensional gas chromatography with time‐of‐flight mass spectrometry (GC × GC/TOFMS) developed in our laboratories,^42^ a very broad range of TPM compounds that was almost fully representative of the known chemical space for tobacco smoke could be analyzed (dark grey and red dots versus light grey dots in Figure 5B). Analytical crossover between techniques was also observed, a substantial fraction of semi‐volatile compounds being amenable for analysis using both GC‐ and LC‐based non‐targeted platforms. In summary, it was demonstrated that, similar to previous results in global metabolic profiling studies,^7^, ^8^ a combination of analytical platforms was able to achieve considerable coverage of the target chemical space.

The coverage achieved using LC/HRAM‐MS was optimized through the use of complementary compound ID strategies. Querying multiple databases in SSA and NTA was key for optimizing not only confidence levels, but also absolute numbers of identified compounds. According to Vinaixa et al,^20^ there is a relatively low overlap of compounds with MS^n^ (*n* ≥2) spectra in existing spectral databases, which explains why most users currently search multiple databases.^15^ NIST MS/MS searches yielded 11 unique compound IDs in our study, whereas UCSD MS^2^ comparisons confirmed 126 chemical constituents in 3R4F‐derived smoke (Figure 5C; “ID Basis” column in Table 1 and Table S1 (supporting information)). A total of 50 constituents identified as being present in tobacco smoke were not listed in UCSD or NIST 14 MS/MS libraries (namely compounds without a PMI code as identifier in Table S1, supporting information). For those compounds, annotation was achieved via *in silico* prediction of MS^2^ spectra based on HMDB, FDA, and ChemIDplus structure databases. This demonstrates the versatility and potential applicability of our NTS workflow for other matrices, and, in that context, continuous efforts are being made to include additional repositories, such as the METLIN MS/MS database^45^ and full integration of the recent US EPA's Chemistry Dashboard^46^ (comprising ~760,000 chemical substances including ~700,000 mapped MS‐Ready structures^47^) into a combined SSA/NTA workflow. Nevertheless, additional efforts will be needed in the future with regards to the identification of compounds from non‐targeted approaches, since annotated spectral data are only available for 5–10% of the small molecules present in major databases. This will require a coordinated effort from the scientific community in our field to meet the needs posed by novel applications in the future.^20^

## 4 CONCLUSIONS

The present study demonstrated the power of using multiple LC/HRAM‐MS‐based chromatographic/ionization approaches combined with complementary strategies for the identification of 3R4F‐derived smoke constituents using SSA and NTA. In total, 331 compounds with semi‐quantitative estimates ≥100 ng per cigarette were identified by LC/HRAM‐MS‐based NTS, which were distributed within the known chemical space for tobacco smoke. Used in combination with complementary NTS approaches based on GC × GC/TOFMS in our laboratories, the potential to analyze a very broad range of compounds, almost fully representative of the known chemical space for tobacco smoke, has been indicated.

Of the four complementary analytical methods employed for LC/HRAM‐MS, RP‐LC/HESI(+) covered a major part with 199 identified compounds. However, it exhibited the highest relative percentage of unconfirmed proposed compounds (~ 60%), because it included many reactive intermediates that were not commercially available as reference standards. As a consequence, additional confirmatory tools such as tR prediction models are considered and planned for the future, in order to increase the confidence for compound identification in the absence of reference standards for absolute confirmation.

Finally, for the first time we have demonstrated an integrated approach that, using different compound identification strategies, including multiple database querying, optimized not only the levels for identification confidence but also the total number of compounds identified. For compounds that were not present in any commercially available or in‐house spectral database, application of an *in silico* fragmentation approach using structural information from HMDB, FDA, and ChemIDplus databases resulted in the identification of 50 novel constituents present in tobacco smoke. This approach provides a benchmark for a fully integrated high‐resolution accurate mass based non‐targeted analysis workflow with subsequent compound ID and semi‐quantification ability. The method has general applicability and offers a huge potential for the analysis of complex matrices in various scientific fields, such as metabolomics and environmental science.

## Supporting information


**Table S1**
Full list of identified compounds in 3R4F‐derived smoke by LC‐HRAM‐MS NTS
**Table S2** Subset of compounds in 3R4F‐derived smoke identified with UCSD MS^2^ and NIST MS/MS libraries
**Figure S1** Identified compounds of tobacco smoke by use of the four separate chromatographic/ionization approaches in LC‐HRAM‐MS‐based NTS
**Figure S2** Differentiation of structural isomers in tobacco smoke using LC‐HRAM‐MS and an experimental MS^2^ fragmentation database
**Figure S3** Distribution of groups of compounds with different identification confidence and isotope similarity within the concentration range
**Figure S4** Base peak and extracted ion chromatograms for two compounds of low concentration for each of the four analytical methods

## References

[1] Dettmer K , Aronov PA , Hammock BD . Mass spectrometry‐based metabolomics. Mass Spectrom Rev. 2007;26(1):51‐78. 16921475 10.1002/mas.20108 PMC1904337

[2] Schymanski EL , Singer HP , Slobodnik J , et al. Non‐target screening with high‐resolution mass spectrometry: Critical review using a collaborative trial on water analysis. Anal Bioanal Chem. 2015;407(21):6237‐6255. 25976391 10.1007/s00216-015-8681-7

[3] Kind T , Tsugawa H , Cajka T , et al. Identification of small molecules using accurate mass MS/MS search. Mass Spectrom Rev. 2017;9999:1‐20. 10.1002/mas.21535 PMC8106966 28436590

[4] Rathahao‐Paris E , Alves S , Junot C , Tabet J‐C . High resolution mass spectrometry for structural identification of metabolites in metabolomics. Metabolomics. 2016;12(1):10‐24.

[5] Rodgman A , Perfetti TA . The Chemical Components of Tobacco and Tobacco Smoke. 2nd ed. Boca Raton, FL: CRC Press; 2013.

[6] Boudah S , Olivier MF , Aros‐Calt S , et al. Annotation of the human serum metabolome by coupling three liquid chromatography methods to high‐resolution mass spectrometry. J Chromatogr B Analyt Technol Biomed Life Sci. 2014;966:34‐47. 10.1016/j.jchromb.2014.04.025 24815365

[7] Lawton KA , Berger A , Mitchell M , et al. Analysis of the adult human plasma metabolome. Pharmacogenomics. 2008;9(4):383‐397. 18384253 10.2217/14622416.9.4.383

[8] van der Werf MJ , Overkamp KM , Muilwijk B , Coulier L , Hankemeier T . Microbial metabolomics: Toward a platform with full metabolome coverage. Anal Biochem. 2007;370(1):17‐25. 17765195 10.1016/j.ab.2007.07.022

[9] Theodoridis G , Gika HG , Wilson ID . LC‐MS‐based methodology for global metabolite profiling in metabonomics/metabolomics. TrAC Trends Anal Chem. 2008;27(3):251‐260.

[10] Theodoridis G , Gika HG , Wilson ID . Mass spectrometry‐based holistic analytical approaches for metabolite profiling in systems biology studies. Mass Spectrom Rev. 2011;30(5):884‐906. 21384411 10.1002/mas.20306

[11] Zhang T , Creek DJ , Barrett MP , Blackburn G , Watson DG . Evaluation of coupling reversed phase, aqueous normal phase, and hydrophilic interaction liquid chromatography with Orbitrap mass spectrometry for metabolomic studies of human urine. Anal Chem. 2012;84(4):1994‐2001. 22409530 10.1021/ac2030738

[12] Imbert L , Gaudin M , Libong D , et al. Comparison of electrospray ionization, atmospheric pressure chemical ionization and atmospheric pressure photoionization for a lipidomic analysis of Leishmania donovani. J Chromatogr A. 2012;1242:75‐83. 22560453 10.1016/j.chroma.2012.04.035

[13] Rivera S , Vilaro F , Canela R . Determination of carotenoids by liquid chromatography/mass spectrometry: Effect of several dopants. Anal Bioanal Chem. 2011;400(5):1339‐1346. 21380750 10.1007/s00216-011-4825-6

[14] Di Guida R , Engel J , Allwood JW , et al. Non‐targeted UHPLC‐MS metabolomic data processing methods: A comparative investigation of normalisation, missing value imputation, transformation and scaling. Metabolomics. 2016;12(5):93‐106. 27123000 10.1007/s11306-016-1030-9 PMC4831991

[15] Lai Z , Tsugawa H , Wohlgemuth G , et al. Identifying metabolites by integrating metabolome databases with mass spectrometry cheminformatics. Nat Methods. 2018;15(1):53‐56. 29176591 10.1038/nmeth.4512 PMC6358022

[16] Dias DA , Jones OA , Beale DJ , et al. Current and future perspectives on the structural identification of small molecules in biological systems. Metabolites. 2016;6(4):46‐74. 10.3390/metabo6040046 PMC5192452 27983674

[17] Dunn WB , Erban A , Weber RJM , et al. Mass appeal: Metabolite identification in mass spectrometry‐focused untargeted metabolomics. Metabolomics. 2013;9(1):44‐66.

[18] Kind T , Fiehn O . Metabolomic database annotations via query of elemental compositions: Mass accuracy is insufficient even at less than 1 ppm. BMC Bioinformatics. 2006;7(1):234‐243. 16646969 10.1186/1471-2105-7-234 PMC1464138

[19] Kind T , Fiehn O . Seven golden rules for heuristic filtering of molecular formulas obtained by accurate mass spectrometry. BMC Bioinformatics. 2007;8(1):105‐124. 17389044 10.1186/1471-2105-8-105 PMC1851972

[20] Vinaixa M , Schymanski EL , Neumann S , Navarro M , Salek RM , Yanes O . Mass spectral databases for LC/MS‐ and GC/MS‐based metabolomics: State of the field and future prospects. TrAC Trends Anal Chem. 2016;78:23‐35.

[21] Hufsky F , Scheubert K , Böcker S . Computational mass spectrometry for small‐molecule fragmentation. TrAC Trends Anal Chem. 2014;53:41‐48. 10.1186/1758-2946-5-12 PMC3648359 23453222

[22] Ruttkies C , Schymanski EL , Wolf S , Hollender J , Neumann S . MetFrag relaunched: Incorporating strategies beyond in silico fragmentation. J Chem. 2016;8(1):3‐18. 10.1186/s13321-016-0115-9 PMC4732001 26834843

[23] Wolf S , Schmidt S , Muller‐Hannemann M , Neumann S . In silico fragmentation for computer assisted identification of metabolite mass spectra. BMC Bioinformatics. 2010;11(1):148‐159. 20307295 10.1186/1471-2105-11-148 PMC2853470

[24] Roemer E , Schramke H , Weiler H , et al. Mainstream smoke chemistry and in vitro and in vivo toxicity of the reference cigarettes 3R4F and 2R4F. Contributions to Tobacco Research. 2012;25(1):316‐335.

[25] Health Canada . Tobacco Products Information Regulations SOR/2000–273, Schedule 2. http://laws-lois.justice.gc.ca/PDF/SOR-2000-273.pdf. Accessed June 4, 2019.

[26] Martin E , Monge A , Duret JA , Gualandi F , Peitsch MC , Pospisil P . Building an R&D chemical registration system. J Chem. 2012;4(1):11‐24. 10.1186/1758-2946-4-11 PMC3430593 22650418

[27] Wishart DS , Feunang YD , Marcu A , et al. HMDB 4.0: The human metabolome database for 2018. Nucleic Acids Res. 2018;46(D1):D608‐D617. 29140435 10.1093/nar/gkx1089 PMC5753273

[28] Yanes O , Tautenhahn R , Patti GJ , Siuzdak G . Expanding coverage of the metabolome for global metabolite profiling. Anal Chem. 2011;83(6):2152‐2161. 21329365 10.1021/ac102981k PMC3285547

[29] Peters FT , Remane D . Aspects of matrix effects in applications of liquid chromatography‐mass spectrometry to forensic and clinical toxicology – a review. Anal Bioanal Chem. 2012;403(8):2155‐2172. 22549818 10.1007/s00216-012-6035-2

[30] Remane D , Meyer MR , Wissenbach DK , Maurer HH . Ion suppression and enhancement effects of co‐eluting analytes in multi‐analyte approaches: Systematic investigation using ultra‐high‐performance liquid chromatography/mass spectrometry with atmospheric‐pressure chemical ionization or electrospray ionization. Rapid Commun Mass Spectrom. 2010;24(21):3103‐3108. 20941756 10.1002/rcm.4736

[31] Roux A , Xu Y , Heilier JF , et al. Annotation of the human adult urinary metabolome and metabolite identification using ultra high performance liquid chromatography coupled to a linear quadrupole ion trap‐Orbitrap mass spectrometer. Anal Chem. 2012;84(15):6429‐6437. 22770225 10.1021/ac300829f

[32] Bushee JL , Argikar UA . An experimental approach to enhance precursor ion fragmentation for metabolite identification studies: Application of dual collision cells in an orbital trap. Rapid Commun Mass Spectrom. 2011;25(10):1356‐1362. 21504000 10.1002/rcm.4996

[33] Mullard G , Allwood JW , Weber R , et al. A new strategy for MS/MS data acquisition applying multiple data dependent experiments on Orbitrap mass spectrometers in non‐targeted metabolomic applications. Metabolomics. 2015;11(5):1068‐1080.

[34] Nonlinear Dynamics . How does the theoretical fragmentation work? http://www.nonlinear.com/progenesis/qi/v2.2/faq/theoretical-fragmentation-algorithm.aspx. Accessed June 4, 2019.

[35] Nonlinear Dynamics . How does database fragmentation scoring work? http://www.nonlinear.com/progenesis/qi/v2.2/faq/database-fragmentation-algorithm.aspx. Accessed June 4, 2019.

[36] Horai H , Arita M , Nishioka T . Comparison of ESI‐MS spectra in MassBank database. *Proc Int Conf Biomed Eng Inform 2*. 2008:853–857.

[37] Nonlinear Dynamics . Identification scoring in Progenesis QI. http://blog.nonlinear.com/2016/04/18/identification‐scoring‐in‐progenesis‐qi/ http://blog.nonlinear.com/2016/04/18/identification-scoring-in-progenesis-qi/ Accessed June 4, 2019.

[38] Weber RJ , Southam AD , Sommer U , Viant MR . Characterization of isotopic abundance measurements in high resolution FT‐ICR and Orbitrap mass spectra for improved confidence of metabolite identification. Anal Chem. 2011;83(10):3737‐3743. 21466230 10.1021/ac2001803

[39] Xu Y , Heilier JF , Madalinski G , et al. Evaluation of accurate mass and relative isotopic abundance measurements in the LTQ‐orbitrap mass spectrometer for further metabolomics database building. Anal Chem. 2010;82(13):5490‐5501. 20515063 10.1021/ac100271j

[40] Sumner LW , Amberg A , Barrett D , et al. Proposed minimum reporting standards for chemical analysis chemical analysis working group (CAWG) metabolomics standards initiative (MSI). Metabolomics. 2007;3(3):211‐221. 24039616 10.1007/s11306-007-0082-2 PMC3772505

[41] Schymanski EL , Jeon J , Gulde R , et al. Identifying small molecules via high resolution mass spectrometry: Communicating confidence. Environ Sci Technol. 2014;48(4):2097‐2098. 24476540 10.1021/es5002105

[42] Knorr A , Almstetter M , Martin E , Castellon A , Pospisil P , Bentley MC . Performance evaluation of a nontargeted platform using two‐dimensional gas chromatography time‐of‐flight mass spectrometry integrating computer‐assisted structure identification and automated Semiquantification for the comprehensive chemical characterization of a complex matrix. Anal Chem. 2019;91(14):9129‐9137. 31265256 10.1021/acs.analchem.9b01659

[43] Stein S . Mass spectral reference libraries: An ever‐expanding resource for chemical identification. Anal Chem. 2012;84(17):7274‐7282. 22803687 10.1021/ac301205z

[44] Vrkoslav V , Urbanova K , Cvacka J . Analysis of wax ester molecular species by high performance liquid chromatography/atmospheric pressure chemical ionisation mass spectrometry. J Chromatogr A. 2010;1217(25):4184‐4194. 20079497 10.1016/j.chroma.2009.12.048

[45] Guijas C , Montenegro‐Burke JR , Domingo‐Almenara X , et al. METLIN: A technology platform for identifying knowns and unknowns. Anal Chem. 2018;90(5):3156‐3164. 29381867 10.1021/acs.analchem.7b04424 PMC5933435

[46] Williams AJ , Grulke CM , Edwards J , et al. The CompTox chemistry dashboard: A community data resource for environmental chemistry. J Chem. 2017;9(1):61‐87. 10.1186/s13321-017-0247-6 PMC5705535 29185060

[47] McEachran AD , Mansouri K , Grulke C , Schymanski EL , Ruttkies C , Williams AJ . "MS‐ready" structures for non‐targeted high‐resolution mass spectrometry screening studies. J Chem. 2018;10(1):45‐60. 10.1186/s13321-018-0299-2 PMC6117229 30167882

