# Supplementary material for: A complex matrix characterization approach, applied to cigarette smoke, that integrates multiple analytical methods and compound identification strategies for non‐targeted liquid chromatography with high‐resolution mass spectrometry
Source: Rapid Commun Mass Spectrom. 2020 Jan 10;34(2):e8571. doi: 10.1002/rcm.8571 (PMC7050541; doi:10.1002/rcm.8571)
Supplement: Supplementary file 1 — Table S1 Full list of identified compounds in 3R4F‐derived smoke by LC‐HRAM‐MS NTS Table S2 Subset of compounds in 3R4F‐derived smoke identified with UCSD MS2 and NIST MS/MS libraries Figure S1 Identified compounds of tobacco smoke by use of the four separate chromatographic/ionization approaches in LC‐HRAM‐MS‐based NTS Figure S2 Differentiation of structural isomers in tobacco smoke using LC‐HRAM‐MS and an experimental MS2 fragmentation database Figure S3 Distribution of groups of compounds with different identification confidence and isotope similarity within the concentration range Figure S4 Base peak and extracted ion chromatograms for two compounds of low concentration for each of the four analytical methods [file RCM-34-e8571-s001.docx]

**Rapid Communications in Mass Spectrometry**

**Supporting information**

**A complex matrix characterization approach, applied to cigarette smoke, that integrates multiple analytical methods and compound identification strategies for non-targeted liquid chromatography with high-resolution mass spectrometry**

Daniel Arndt*, Christian Wachsmuth*, Christoph Buchholz, Mark Bentley

PMI R&D, Philip Morris Products S.A., Quai Jeanrenaud 5, CH-2000 Neuchâtel, Switzerland

*These authors contributed equally to this work and therefore should be considered equal first authors.

**Table S1** Full list of identified compounds in 3R4F-derived smoke by LC-HRAM-MS NTS

**Table S2** Subset of compounds in 3R4F-derived smoke identified with UCSD MS^2^ and NIST MS/MS libraries

**Figure S1** Identified compounds of tobacco smoke by use of the four separate chromatographic/ionization approaches in LC-HRAM-MS-based NTS

**Figure S2** Differentiation of structural isomers in tobacco smoke using LC-HRAM-MS and an experimental MS^2^ fragmentation database

**Figure S3** Distribution of groups of compounds with different identification confidence and isotope similarity within the concentration range

**Figure S4** Base peak and extracted ion chromatograms for two compounds of low concentration for each of the four analytical methods

**Table S1 Full list of identified compounds in 3R4F-derived smoke by LC-HRAM-MS NTS.** Compounds are sorted in descending order of yield (microgram per cigarette).

Confidence levels: dark green, confirmed: tR and mass spectra within specified tolerance ranges in comparison to a standard under the same experimental conditions; light green, high: overall score > 50 or overall score > 45 and FS > 45; yellow, medium: overall score < 45 or overall score between 45 and 50 and FS < 45.

*m/z* expt, *m/z* quantifier ion from method specified in “method” column; RSD, relative standard deviation (N = 15 total observations from three sample replicates that were injected fivefold); LogVP, log-transformed vapor pressure; LogP_OW_, log-transformed octanol/water partition coefficient; FS, fragmentation score; Δm, difference between experimental and theoretical mass; ΔtR, difference between tR in a sample and standard in database; na, not available. Method highlighted in bold: method from which the information on the analytical figures were extracted, for cases where compounds were identified with multiple analytical methods

| **#** | | **Name** | **Identifier** | **CAS** | **Formula** | **Yield (µg/cig)** | ***m/z* expt** | **tR (min)** | **RSD  (%)** | **LogVP  (mmHg)** | **LogP_OW_** | **Overall Score** | **FS** | **Δm (ppm)** | **Isotope Similarity** | **ΔtR  (min)** | | **Method** | **ID Basis** |
| --- | --- | --- | --- | --- | --- | --- | --- | --- | --- | --- | --- | --- | --- | --- | --- | --- | --- | --- | --- |
| 1 | | **Solanesol** | PMI0000409 | 13190-97-1 | C45H74O | 6762.02 | 648.60630 | 17.08 | 2.7 | 0.0 | 0.0 | 71.0 | 91.3 | -2.4 | 99.8 | 0.0 | | **RP-HESI(+)**, RP-APCI(+) | UCSD MS^2^ |
| 2 | | **Nicotine** | PMI0004286 | 22083-74-5 | C10H14N2 | 3006.20 | 163.12266 | 3.32 | 2.9 | -3.5 | 0.7 | 59.3 | 98.9 | -1.9 | 89.7 | -0.1 | | **RP-HESI(+)**, RP-APCI(+), HILIC-HESI(+) | UCSD MS^2^ |
| 3 | | **Bombiprenone** | PMI0006795 | 21978-49-4 | C43H70O | 1213.51 | 620.57552 | 16.76 | 3.3 | -39.1 | 16.3 | 42.7 | 15.3 | -1.0 | 99.4 | na | | **RP-HESI(+)**, RP-APCI(+) | UCSD *in silico* MS^2^ |
| 4 | | **Triacetin** | PMI0000113 | 102-76-1 | C9H14O6 | 585.34 | 236.11230 | 4.03 | 5.2 | -4.3 | -0.2 | 76.0 | 100.0 | -2.2 | 99.3 | 0.0 | | RP-HESI(+) | UCSD MS^2^ |
| 5 | | **7-Ketositosterol** | PMI0009304 | 2034-74-4 | C29H48O2 | 534.10 | 429.37223 | 12.30 | 7.3 | -29.9 | 8.5 | 44.9 | 30.3 | -1.1 | 95.5 | na | | **RP-HESI(+)**, RP-APCI(+), HILIC-HESI(+) | UCSD *in silico* MS^2^ |
| 6 | | **Pytoene Carotenoid** | HMDB39093 | 13920-14-4 | C40H64 | 492.91 | 545.50760 | 15.51 | 4.2 | -32.0 | 16.6 | 41.8 | 11.7 | -0.9 | 98.5 | na | | RP-HESI(+) | HMDB *in silico* MS^2^ |
| 7 | | **5,9,13,17,21,25,29-Hentriacontaheptaen-2-one, 6,10,14,18,22,26,30-heptamethyl** | PMI0006129 | 6704-02-5 | C38H62O | 446.72 | 552.51270 | 15.19 | 3.7 | -33.2 | 14.3 | 46.0 | 33.7 | -0.9 | 97.5 | na | | RP-HESI(+) | UCSD *in silico* MS^2^ |
| 8 | | **(3β)-3-Methylandrost-5-en-17-one** | CSID114234 | 90468-14-7 | C20H30O | 410.89 | 287.23637 | 8.39 | 3.1 | -12.9 | 5.9 | 50.4 | 56.9 | -2.0 | 97.4 | na | | RP-HESI(+) | ChemIDplus *in silico* MS^2^ |
| 9 | | **Palmitic acid** | PMI0000164 | 57-10-3 | C16H32O2 | 396.90 | 255.23332 | 9.78 | 4.0 | -10.3 | 7.2 | 56.6 | 59.2 | 1.4 | 98.1 | 0.1 | | RP-HESI(-) | UCSD MS^2^ |
| 10 | | **N-Octanoylnornicotine** | PMI0001863 | 38854-10-3 | C17H26N2O | 394.08 | 275.21140 | 7.21 | 4.4 | -16.3 | 3.4 | 62.8 | 58.2 | -1.4 | 97.5 | 0.0 | | **RP-HESI(+)**, RP-APCI(+), HILIC-HESI(+) | UCSD MS^2^ |
| 11 | | **Solanochromene** | PMI0008547 | 56084-94-7 | C53H80O2 | 274.79 | 766.64837 | 18.77 | 4.1 | 0.0 | 0.0 | 48.6 | 49.3 | -2.1 | 96.5 | na | | **RP-HESI(+)**, RP-APCI(+) | UCSD *in silico* MS^2^ |
| 12 | | **Linolenic acid** | PMI0000169 | 463-40-1 | C18H30O2 | 258.65 | 277.21765 | 9.11 | 4.4 | -19.3 | 6.5 | 48.8 | 27.5 | 1.2 | 97.8 | 0.1 | | RP-HESI(+), **RP-HESI(-)** | UCSD MS^2^ |
| 13 | | **Scopoletin** | PMI0000309 | 92-61-5 | C10H8O4 | 232.56 | 193.04923 | 3.61 | 1.5 | -15.4 | 1.3 | 61.7 | 78.7 | -1.6 | 99.2 | 0.0 | | **RP-HESI(+)**, RP-APCI(+), RP-HESI(-) | UCSD MS^2^ |
| 14 | | **2,7,12-Cyclotetradecatrien-1-ol, 1,7-dimethyl-11methylene-4-(1-methylethyl)-** | PMI0008333 | 60026-11-1 | C20H32O | 217.63 | 289.25194 | 8.58 | 3.2 | -17.0 | 7.3 | 56.7 | 88.9 | -2.3 | 97.5 | na | | **RP-HESI(+)**, RP-APCI(+) | UCSD *in silico* MS^2^ |
| 15 | | **Linolic acid** | PMI0000168 | 60-33-3 | C18H32O2 | 207.07 | 279.23333 | 9.50 | 4.4 | -12.6 | 7.2 | 57.2 | 64.0 | 1.3 | 97.7 | 0.1 | | RP-HESI(-) | UCSD MS^2^ |
| 16 | | **N-Formylnornicotine** | PMI0006520 | 38840-03-8 | C10H12N2O | 206.38 | 177.10195 | 2.94 | 3.8 | -10.8 | -0.2 | 64.5 | 61.1 | -1.6 | 97.9 | 0.0 | | **RP-HESI(+)**, RP-APCI(+), HILIC-HESI(+) | UCSD MS^2^ |
| 17 | | **(all-E)-6,10,14,18,22,26-hexamethyl-5,9,13,17,21,25-Heptacosahexaen-2-one** | PMI0006786 | 32304-17-9 | C33H54O | 150.60 | 484.45070 | 13.81 | 4.7 | -27.6 | 12.3 | 44.8 | 28.1 | -0.9 | 97.2 | na | | RP-HESI(+) | UCSD *in silico* MS^2^ |
| 18 | | **α-Levantenolide** | PMI0008002 | 5989-73-1 | C20H30O3 | 149.50 | 319.22598 | 7.74 | 2.4 | -16.6 | 4.6 | 41.1 | 11.9 | -2.5 | 96.7 | na | | RP-HESI(+) | UCSD *in silico* MS^2^ |
| 19 | | **Solanesyl acetate** | PMI0008537 | 29144-38-5 | C47H76O2 | 147.48 | 690.61729 | 18.75 | 3.5 | 0.0 | 0.0 | 39.7 | 9.9 | -1.6 | 90.6 | na | | RP-HESI(+) | UCSD *in silico* MS^2^ |
| 20 | | **1,3,5,7,11-Cembrapentaene, (1E,3Z,5E,7Z,11E)** | PMI0009274 | 420793-93-7 | C20H30 | 132.14 | 271.24145 | 9.86 | 3.1 | -11.3 | 8.8 | 52.0 | 64.6 | -2.1 | 97.8 | na | | **RP-HESI(+)**, RP-APCI(+) | UCSD *in silico* MS^2^ |
| 21 | | **14,15-Dinor-8-labdene-7,13-dione** | PMI0006630 | 72446-33-4 | C18H28O2 | 127.92 | 277.21576 | 7.59 | 2.0 | -12.3 | 4.1 | 42.4 | 16.1 | -1.6 | 97.8 | na | | RP-HESI(+) | UCSD *in silico* MS^2^ |
| 22 | | **Cotinine** | PMI0001948 | 486-56-6 | C10H12N2O | 127.34 | 177.10197 | 2.73 | 3.1 | -7.8 | -0.2 | 53.5 | 69.4 | -1.6 | 89.7 | -0.1 | | **RP-HESI(+)**, RP-APCI(+), HILIC-HESI(+) | UCSD MS^2^ |
| 23 | | **Stearic acid** | PMI0000166 | 57-11-4 | C18H36O2 | 125.58 | 283.26469 | 10.58 | 2.3 | -11.7 | 8.2 | 60.6 | 77.6 | 1.5 | 97.7 | 0.1 | | RP-HESI(-) | UCSD MS^2^ |
| 24 | | **Piperidine, 1-(3-pyridinemethyl)-2-cyano-4,5didehydro** | PMI0007755 | na | C12H13N3 | 124.59 | 200.11786 | 4.58 | 4.0 | -11.4 | 0.2 | 43.6 | 33.0 | -1.8 | 87.3 | na | | **RP-HESI(+)**, RP-APCI(+), HILIC-HESI(+) | UCSD *in silico* MS^2^ |
| 25 | | **β-Levantenolide** | PMI0008043 | 30987-49-6 | C20H30O3 | 123.32 | 319.22634 | 6.85 | 3.0 | -16.6 | 4.6 | 41.5 | 11.9 | -1.4 | 97.3 | na | | RP-HESI(+) | UCSD *in silico* MS^2^ |
|  | |  |  |  |  |  |  |  |  |  |  |  |  |  |  |  | |  |  |
| 26 | | **Hydroxyretinol** | PMI0012017 | 6890-93-3 | C20H30O2 | 123.01 | 303.23134 | 7.82 | 4.7 | -22.4 | 5.0 | 45.8 | 33.9 | -1.7 | 97.4 | na | | RP-HESI(+) | UCSD *in silico* MS^2^ |
| 27 | | **3-Methoxybenzidine** | PMI0011783 | 3365-87-5 | C13H14N2O | 122.28 | 215.11758 | 4.91 | 1.9 | -11.7 | 1.6 | 50.5 | 58.9 | -1.4 | 95.2 | na | | RP-HESI(+) | UCSD *in silico* MS^2^ |
| 28 | | **5-Cyanonicotine** | PMI0000263 | 42459-12-1 | C11H13N3 | 119.03 | 188.11793 | 4.64 | 6.8 | -9.1 | -0.3 | 73.5 | 100.0 | -1.5 | 88.4 | 0.0 | | **RP-HESI(+)**, RP-APCI(+), HILIC-HESI(+) | UCSD MS^2^ |
| 29 | | **1'-(7-Hydroxyoctanoyl)nornicotine** | PMI0005728 | 77829-18-6 | C17H26N2O2 | 116.98 | 291.20613 | 5.28 | 4.4 | -21.7 | 1.2 | 47.9 | 44.8 | -2.0 | 97.1 | na | | **RP-HESI(+)**, RP-APCI(+) | UCSD *in silico* MS^2^ |
| 30 | | **Cholesteryl acetate** | PMI0011804 | 604-35-3 | C29H48O2 | 112.46 | 429.37223 | 13.75 | 12.2 | -21.1 | 10.7 | 38.7 | 0.0 | -1.1 | 95.0 | na | | RP-HESI(+) | NIST MS/MS |
| 31 | | **N'-Carbomethoxyanabasine** | PMI0007363 | 56078-09-2 | C12H16N2O2 | 106.28 | 221.12802 | 1.27 | 1.9 | -9.8 | 0.9 | 48.6 | 47.3 | -2.0 | 98.2 | na | | **RP-HESI(+)**, RP-APCI(+) | UCSD *in silico* MS^2^ |
| 32 | | **3-hydroxy-5-methoxy-6-methyl-3,4-dihydro-2H-pyran-4-one** | HMDB36380 | na | C7H10O4 | 106.11 | 159.06512 | 4.03 | 8.0 | -7.8 | -0.3 | 45.5 | 28.2 | -0.4 | 99.4 | na | | RP-APCI(+) | HMDB *in silico* MS^2^ |
| 33 | | **Damascenone** | PMI0000304 | 23726-93-4 | C13H18O | 105.15 | 191.14268 | 7.44 | 3.9 | -5.3 | 4.0 | 59.1 | 89.7 | -1.9 | 98.5 | -0.3 | | RP-HESI(+) | UCSD MS^2^ |
| 34 | | **2-Cyclohexen-1-one, 2,4,4-trimethyl-3-(1,3butadienyl)** | PMI0007742 | 84696-84-4 | C13H18O | 103.85 | 191.14267 | 7.31 | 3.4 | -6.2 | 3.2 | 51.6 | 61.9 | -2.0 | 98.3 | na | | **RP-HESI(+)**, RP-APCI(+) | UCSD *in silico* MS^2^ |
| 35 | | **Phytoene** | PMI0007615 | 540-04-5 | C40H64 | 97.09 | 545.50769 | 18.55 | 3.8 | -32.0 | 16.6 | 50.2 | 43.9 | -0.7 | 96.1 | 0.6 | | **RP-HESI(+)**, RP-APCI(+) | UCSD MS^2^ |
| 36 | | **N-Acetyl-Anabasine** | PMI0011813 | 91557-10-7 | C12H16N2O | 96.53 | 205.13322 | 4.72 | 4.9 | -12.0 | 0.8 | 42.5 | 33.0 | -1.6 | 81.3 | na | | **RP-HESI(+)**, RP-APCI(+), HILIC-HESI(+) | UCSD *in silico* MS^2^ |
| 37 | | **(1S,4R,2E,7E,11E)-6-Keto-2,7,11-cembratriene-4-ol** | PMI0000995 | 57760-52-8 | C20H32O2 | 96.40 | 305.24693 | 7.46 | 5.1 | -19.8 | 5.8 | 45.9 | 34.5 | -1.9 | 97.3 | na | | RP-HESI(+) | UCSD *in silico* MS^2^ |
| 38 | | **2-(5-Methylfuran-2-yl)-5-[(2S)-1-methylpyrrolidin-2-yl]pyridine** | PMI0000955 | na | C15H18N2O | 95.95 | 243.14878 | 7.66 | 1.8 | -9.1 | 2.3 | 41.4 | 12.2 | -1.7 | 96.7 | na | | RP-HESI(+) | UCSD *in silico* MS^2^ |
| 39 | | **5-Methylcotinine** | PMI0009912 | 1076198-50-9 | C11H14N2O | 92.27 | 191.11747 | 3.26 | 3.1 | -11.2 | 0.2 | 45.8 | 29.2 | -2.2 | 97.7 | -0.3 | | **RP-HESI(+)**, HILIC-HESI(+) | UCSD MS^2^ |
| 40 | | **Norharman** | PMI0000439 | 244-63-3 | C11H8N2 | 87.72 | 169.07575 | 6.00 | 2.5 | -12.1 | 2.8 | 51.6 | 63.9 | -1.6 | 88.6 | -0.3 | | **RP-HESI(+)**, RP-APCI(+), HILIC-HESI(+) | UCSD MS^2^ |
| 41 | | **1-Palmitoyl-4-(palmitoyloxy)proline** | CSID2295947 | na | C37H69NO5 | 87.16 | 608.52398 | 14.37 | 4.5 | na | na | 40.6 | 9.5 | -1.4 | 95.3 | na | | RP-HESI(+) | ChemIDplus *in silico* MS^2^ |
| 42 | | **Linolenin, 1-mono-** | PMI0000384 | 18465-99-1 | C21H36O4 | 85.65 | 353.26796 | 9.25 | 4.1 | -24.7 | 5.5 | 56.5 | 87.9 | -1.9 | 97.0 | na | | RP-HESI(+) | NIST MS/MS |
| 43 | | **Stigmasta-3,5-diene** | PMI0007573 | 79897-80-6 | C29H48 | 84.89 | 397.38217 | 12.36 | 4.4 | -18.9 | 12.2 | 45.5 | 34.7 | -1.8 | 95.1 | na | | RP-HESI(+) | UCSD *in silico* MS^2^ |
| 44 | | **Cohibin C** | HMDB35397 | 293735-20-3 | C37H68O4 | 78.58 | 577.51814 | 15.37 | 4.3 | na | na | 42.0 | 21.5 | -1.5 | 90.2 | na | | RP-HESI(+) | HMDB *in silico* MS^2^ |
| 45 | | **Pyrrolidine, 1-(6-hydroxy-1-oxooctyl)-2-(3-pyridinyl)** | PMI0005729 | 77829-17-5 | C17H26N2O2 | 77.31 | 291.20614 | 5.18 | 4.2 | -21.7 | 1.2 | 75.0 | 98.0 | -1.9 | 96.7 | 0.0 | | **RP-HESI(+),** RP-APCI(+), HILIC-HESI(+) | UCSD MS^2^ |
| 46 | | **Moupinamide** | PMI0001823 | 66648-43-9 | C18H19NO4 | 76.22 | 314.13811 | 4.78 | 2.8 | -33.2 | 2.0 | 59.6 | 96.6 | -1.8 | 97.1 | -0.3 | | **RP-HESI(+),** RP-APCI(+), RP-HESI(-), | UCSD MS^2^ |
| 47 | | **28-Homoteasterone** | PMI0011841 | 90524-90-6 | C29H50O4 | 75.06 | 463.37760 | 11.90 | 3.2 | -36.3 | 5.0 | 53.4 | 70.0 | -1.3 | 98.6 | na | | RP-HESI(+) | UCSD *in silico* MS^2^ |
| 48 | | **Methyl 3-[5-(2-oxiranyl)pentyl]undecanoate** | CSID83890 | 2500-59-6 | C19H36O3 | 74.96 | 313.27302 | 9.92 | 5.3 | -12.6 | 6.3 | 42.8 | 19.9 | -2.3 | 96.7 | na | | **RP-HESI(+),** RP-APCI(+) | ChemIDplus *in silico* MS^2^ |
| 49 | | **(1E)-5-Methyl-1-(3,4,5-trimethoxyphenyl)-1-hexen-3-one** | CSID4950306 | 66596-38-1 | C16H22O4 | 74.01 | 279.15989 | 5.03 | 4.2 | -14.3 | 3.2 | 40.1 | 8.3 | 2.9 | 96.0 | na | | RP-HESI(+) | ChemIDplus *in silico* MS^2^ |
| 50 | | **Piperidine, 1-(3-pyridinemethyl)-2-cyano** | PMI0007756 | na | C12H15N3 | 72.95 | 202.13355 | 5.21 | 1.5 | -10.5 | 0.3 | 42.7 | 28.4 | -1.6 | 87.3 | na | | **RP-HESI(+),** RP-APCI(+) | UCSD *in silico* MS^2^ |
| 51 | | **2,4,9,13-Cyclotetradecatetraen-1-ol, 3,9,13trimethyl-6-(1-methylethyl)** | PMI0008332 | 39815-66-2 | C20H32O | 72.01 | 289.25198 | 8.86 | 4.1 | -17.5 | 7.3 | 56.7 | 88.6 | -2.1 | 97.6 | na | | RP-HESI(+) | UCSD *in silico* MS^2^ |
| 52 | | **1-(1-Oxohexyl)-2-(3-pyridinyl)-Pyrrolidine** | PMI0006608 | 38854-09-0 | C15H22N2O | 69.52 | 247.18003 | 6.08 | 2.8 | -14.4 | 2.4 | 63.7 | 88.5 | -1.9 | 97.3 | 0.0 | | **RP-HESI(+),** RP-APCI(+), HILIC-HESI(+) | UCSD MS^2^ |
| 53 | | **4-Phenyl-2-quinolinol** | CSID333458 | 5855-57-2 | C15H11NO | 66.34 | 239.11749 | 6.72 | 3.2 | -18.7 | 3.8 | 48.2 | 48.1 | -1.7 | 94.7 | na | | RP-HESI(+) | ChemIDplus *in silico* MS^2^ |
| 54 | | **1-(2-Icosanyl)naphthalene** | CSID77783 | 135585-40-9 | C30H48 | 65.70 | 409.38239 | 12.80 | 4.0 | -21.5 | 13.8 | 39.3 | 1.8 | -1.2 | 96.3 | na | | RP-HESI(+) | ChemIDplus *in silico* MS^2^ |
| 55 | | **2-Methyl-3-phenyl-pyrazine** | PMI0009736 | 29444-53-9 | C11H10N2 | 65.54 | 171.09137 | 5.26 | 2.4 | -4.1 | 2.3 | 50.6 | 49.6 | -1.8 | 96.3 | -0.2 | | **RP-HESI(+),** RP-APCI(+) | UCSD MS^2^ |
| 56 | | **Pyridine, 3-[1-(5-ethyl-2-furanyl)-1H-pyrrol-2-yl]** | PMI0005488 | 78210-88-5 | C15H14N2O | 64.50 | 239.11748 | 6.94 | 2.1 | -13.0 | 3.8 | 46.0 | 34.5 | -1.7 | 97.8 | na | | **RP-HESI(+),** RP-APCI(+) | UCSD *in silico* MS^2^ |
| 57 | | **2-(5,6,7,8-Tetrahydro-1-naphthalenyl)acetamide** | CSID23896 | 13052-98-7 | C12H15NO | 62.98 | 190.12227 | 6.30 | 2.6 | -12.6 | 2.1 | 47.7 | 44.1 | -1.9 | 96.7 | na | | RP-HESI(+) | ChemIDplus *in silico* MS^2^ |
| 58 | | **N'-Formylanatabine** | PMI0006544 | 61892-65-7 | C11H12N2O | 14.02 | 189.10205 | 3.55 | 1.8 | -10.8 | 0.0 | 50.4 | 53.3 | -1.0 | 92.2 | 0.2 | | RP-HESI(+), **RP-APCI(+)**, HILIC-HESI(+) | UCSD MS^2^ |
| 59 | | **(8E,13cis)-10-Hydroxy-11,12-didehydro-7,10-dihydroretinol** | CSID4945473 | 3230-75-9 | C20H30O2 | 61.51 | 303.23131 | 8.23 | 3.7 | -21.7 | 7.2 | 42.7 | 18.4 | -1.8 | 97.1 | na | | RP-HESI(+) | ChemIDplus *in silico* MS^2^ |
| 60 | | **Myosmine** | PMI0004067 | 532-12-7 | C9H10N2 | 60.00 | 147.09140 | 4.07 | 4.9 | -3.1 | -0.6 | 56.1 | 77.9 | -1.9 | 98.9 | -0.3 | | **RP-HESI(+)**, RP-APCI(+), HILIC-HESI(+) | UCSD MS^2^ |
| 61 | | **Harmaline** | PMI0002136 | 304-21-2 | C13H14N2O | 59.82 | 215.11756 | 4.67 | 2.4 | -12.1 | 1.0 | 50.0 | 54.5 | -1.5 | 97.5 | na | | **RP-HESI(+)**, RP-APCI(+), HILIC-HESI(+) | NIST MS/MS |
| 62 | | **Stigmasta-3,5,24(28)-triene** | PMI0005974 | 86709-50-4 | C29H46 | 59.01 | 395.36670 | 12.10 | 4.6 | -19.0 | 11.9 | 47.0 | 42.9 | -1.3 | 93.7 | na | | RP-HESI(+) | UCSD *in silico* MS^2^ |
| 63 | | **Nicotelline** | PMI0005465 | 494-04-2 | C15H11N3 | 58.99 | 234.10219 | 5.17 | 2.0 | -13.9 | 2.1 | 62.3 | 95.3 | -1.7 | 98.2 | -0.1 | | **RP-HESI(+)**, RP-APCI(+), HILIC-HESI(+) | UCSD MS^2^ |
| 64 | | **Pyrrolo[1,2-a]pyrazine-1,4-dione, hexahydro-3-(2methylpropyl)** | PMI0000046 | 5654-86-4 | C11H18N2O2 | 58.70 | 211.14366 | 3.85 | 2.5 | -15.6 | -0.8 | 60.5 | 75.0 | -2.1 | 98.1 | 0.0 | | **RP-HESI(+)**, RP-APCI(+) | UCSD MS^2^ |
| 65 | | **3,4-Dimethyl-5-pentyl-2-furanundecanoic acid** | PMI0011800 | 57818-36-7 | C22H38O3 | 58.16 | 351.28875 | 9.87 | 6.2 | -21.0 | 8.6 | 53.5 | 73.6 | -1.8 | 96.1 | na | | RP-HESI(+) | UCSD *in silico* MS^2^ |
| 66 | | **Ergosta-3,5,7-triene, (24epsilon)** | PMI0007564 | 77327-07-2 | C28H46 | 58.15 | 383.36658 | 12.07 | 4.2 | -17.8 | 11.7 | 47.8 | 45.8 | -1.7 | 95.1 | na | | RP-HESI(+) | UCSD *in silico* MS^2^ |
| 67 | | **6-(Heptyloxy)-3-pyridinamine** | PMI0011838 | 857219-70-6 | C12H20N2O | 57.29 | 209.16448 | 6.77 | 2.6 | -9.4 | 3.7 | 46.5 | 47.6 | -1.7 | 86.9 | na | | **RP-HESI(+)**, RP-APCI(+), RP-HESI(-), HILIC-HESI(+) | UCSD *in silico* MS^2^ |
| 68 | | **N-Methyl-N-palmitoylglycine** | PMI0011852 | 2421-33-2 | C19H37NO3 | 55.31 | 328.28393 | 9.69 | 4.6 | -21.2 | 6.5 | 45.8 | 35.0 | -2.1 | 96.7 | na | | RP-HESI(+) | UCSD *in silico* MS^2^ |
| 69 | | **1,2,7,7',8,8',11',12'-Octahydro-psi,psi-caroten-1-ol** | CSID4947056 | 29753-46-6 | C40H64O | 53.13 | 561.50264 | 13.19 | 3.6 | na | na | 49.0 | 50.3 | -0.6 | 95.6 | na | | RP-HESI(+) | ChemIDplus *in silico* MS^2^ |
| 70 | | **4-Hydroxy-4-(3-pyridyl)butyric acid** | PMI0011329 | 15569-97-8 | C9H11NO3 | 52.67 | 199.10739 | 1.54 | 3.1 | -15.4 | -0.4 | 51.3 | 59.9 | -1.8 | 98.7 | na | | RP-HESI(+) | UCSD *in silico* MS^2^ |
| 71 | | **4,8,13-Cyclotetradecatriene-1,3-diol, 1,5,9-trimethyl-12-(1-methylethyl)-, 3-acetate, [1S(1R*,3S*,4E,8E,12S*,13E)]** | PMI0000992 | 82043-08-1 | C22H36O3 | 51.65 | 349.27309 | 9.71 | 4.2 | -20.2 | 6.8 | 48.7 | 59.4 | -1.8 | 86.3 | na | | RP-HESI(+) | UCSD *in silico* MS^2^ |
| 72 | | **Harman** | PMI0000269 | 486-84-0 | C12H10N2 | 50.93 | 183.09141 | 6.13 | 3.4 | -11.8 | 3.3 | 50.7 | 57.0 | -1.5 | 87.5 | -0.2 | | **RP-HESI(+)**, RP-APCI(+), HILIC-HESI(+) | UCSD MS^2^ |
|  | |  |  |  |  |  |  |  |  |  |  |  |  |  |  |  | |  |  |
| 73 | | **9H-Pyrido[3,4-b]indole, 1-ethyl** | PMI0005131 | 20127-61-1 | C13H12N2 | 50.55 | 197.10703 | 6.50 | 2.4 | -12.0 | 3.8 | 55.1 | 54.7 | -1.5 | 93.3 | 0.1 | | **RP-HESI(+)**, HILIC-HESI(+) | UCSD MS^2^ |
| 74 | | **(3β)-Ergosta-5,25-dien-3-ol** | PMI0006712 | 52936-69-3 | C28H46O | 50.02 | 399.36118 | 13.27 | 3.3 | -25.2 | 9.9 | 53.1 | 73.4 | -2.4 | 95.2 | na | | RP-HESI(+) | UCSD *in silico* MS^2^ |
| 75 | | **Pyridine, 3-[1-(5-propyl-2-furanyl)-1H-pyrrol-2-yl]** | PMI0005569 | 78210-89-6 | C16H16N2O | 48.70 | 253.13307 | 7.38 | 2.1 | -13.8 | 4.4 | 45.4 | 32.3 | -1.9 | 96.9 | na | | RP-HESI(+) | UCSD *in silico* MS^2^ |
| 76 | | **3-Methyl-5H-pyrido(4,3-b)indole** | CSID4548099 | 58096-07-4 | C12H10N2 | 48.44 | 183.09143 | 6.60 | 2.2 | -11.8 | 2.7 | 40.6 | 30.6 | -1.3 | 74.2 | na | | RP-HESI(+) | ChemIDplus *in silico* MS^2^ |
| 77 | | **(2Z)-3-(3-Aminophenyl)-2-(4-methoxyphenyl)acrylonitrile** | CSID4820621 | 5462-59-9 | C16H14N2O | 48.05 | 251.11736 | 6.84 | 3.6 | -17.8 | 3.1 | 49.8 | 54.6 | -2.1 | 96.8 | na | | RP-HESI(+) | ChemIDplus *in silico* MS^2^ |
| 78 | | **α-Cyperone** | PMI0007359 | 473-08-5 | C15H22O | 47.78 | 219.17397 | 7.91 | 2.7 | -8.1 | 4.2 | 51.8 | 54.7 | -1.7 | 97.7 | -0.4 | | **RP-HESI(+)**, RP-APCI(+) | UCSD MS^2^ |
| 79 | | **N-({4-[4-(2-Methyl-1H-imidazol-1-yl)butyl]phenyl}acetyl)-L-seryl-N-(2-cyclohexylethyl)-L-lysinamide** | CSID393759 | 164931-25-3 | C33H52N6O4 | 47.60 | 597.41200 | 11.78 | 3.8 | 0.0 | 3.1 | 39.8 | 0.4 | -0.5 | 99.0 | na | | RP-HESI(+) | ChemIDplus *in silico* MS^2^ |
| 80 | | **4-Piperidinol, 4-(2-methylphenyl)-** | PMI0011366 | 83674-76-4 | C12H17NO | 47.34 | 209.16446 | 7.06 | 2.5 | -9.7 | 1.6 | 40.5 | 6.6 | -1.1 | 97.1 | na | | RP-HESI(+) | UCSD *in silico* MS^2^ |
| 81 | | **Nicoteine** | PMI0004386 | 366-18-7 | C10H8N2 | 47.14 | 157.07584 | 4.38 | 4.2 | -4.6 | 1.3 | 49.4 | 55.4 | -1.2 | 89.5 | -0.5 | | **RP-HESI(+)**, RP-APCI(+), HILIC-HESI(+) | UCSD MS^2^ |
| 82 | | **Cholesteryl linolenate** | PMI0007837 | 2545-22-4 | C45H74O2 | 46.33 | 664.60150 | 16.13 | 10.9 | 0.0 | 0.0 | 38.9 | 0.0 | -1.9 | 96.6 | na | | RP-HESI(+) | NIST MS/MS |
| 83 | | **Isolinderenolide** | HMDB38104 | 139328-80-6 | C21H34O3 | 45.04 | 335.25723 | 9.93 | 3.4 | -24.4 | 7.2 | 41.6 | 15.0 | -2.5 | 96.1 | na | | RP-HESI(+) | HMDB *in silico* MS^2^ |
| 84 | | **1-(2,3-Dihydro-1H-indol-4-yloxy)-3-(isopropylamino)-2-propanol** | CSID2322363 | 79364-16-2 | C14H22N2O2 | 43.89 | 251.17487 | 2.12 | 7.6 | -18.8 | 1.6 | 51.3 | 61.2 | -2.1 | 97.6 | na | | RP-HESI(+) | ChemIDplus *in silico* MS^2^ |
| 85 | | **N-Butylbenzamide** | CSID68522 | 2782-40-3 | C11H15NO | 43.74 | 195.14889 | 6.06 | 3.0 | -9.4 | 2.5 | 43.0 | 28.8 | -1.7 | 88.4 | na | | RP-HESI(+) | ChemIDplus *in silico* MS^2^ |
| 86 | | **2-Naphthaleneethanol, 3,4-dihydro-1,5,6-trimethyl** | PMI0005342 | 102977-87-7 | C15H20O | 43.60 | 217.15839 | 7.85 | 2.7 | -10.6 | 4.3 | 47.3 | 40.7 | -1.4 | 97.4 | na | | RP-HESI(+) | UCSD *in silico* MS^2^ |
| 87 | | **1-[4-(Dimethylamino)-2-butyn-1-yl]-5-methyl-2-pyrrolidinone** | PMI0011847 | 98673-90-6 | C11H18N2O | 41.43 | 195.14888 | 6.22 | 2.7 | -8.5 | 0.7 | 41.4 | 20.8 | -1.5 | 88.1 | na | | **RP-HESI(+)**, RP-APCI(+), HILIC-HESI(+) | UCSD *in silico* MS^2^ |
| 88 | | **2-Piperidone, 3,4-dehydro-** | PMI0002565 | 6052-73-9 | C5H7NO | 40.99 | 98.06046 | 1.51 | 2.9 | -6.0 | -0.6 | 60.7 | 77.4 | 4.3 | 99.2 | 0.0 | | RP-HESI(+) | UCSD MS^2^ |
| 89 | | **α-Tocoquinone** | PMI0007590 | 7559-04-8 | C29H50O3 | 40.50 | 429.37258 | 12.38 | 7.1 | -29.9 | 10.4 | 60.6 | 89.8 | -0.3 | 95.0 | 0.2 | | RP-HESI(+), **RP-APCI(+)**, RP-HESI(-) | UCSD MS^2^ |
| 90 | | **Nicotyrin-2-aldehyde** | PMI0001883 | 3614-77-5 | C11H10N2O | 40.44 | 187.08631 | 5.38 | 1.9 | -10.5 | 0.9 | 41.0 | 18.7 | -1.5 | 88.3 | na | | **RP-HESI(+)**, RP-HESI(-) | UCSD *in silico* MS^2^ |
| 91 | | **Docosanoic acid** | PMI0005889 | 112-85-6 | C22H44O2 | 40.07 | 339.32736 | 12.36 | 4.2 | -14.1 | 10.3 | 62.9 | 90.3 | 1.5 | 96.8 | 0.1 | | RP-HESI(-) | UCSD MS^2^ |
| 92 | | **3-Pyridinol** | PMI0000271 | 109-00-2 | C5H5NO | 39.06 | 96.04485 | 1.73 | 2.6 | -8.6 | 0.6 | 54.7 | 77.2 | 4.8 | 94.6 | -0.1 | | RP-HESI(+) | UCSD MS^2^ |
| 93 | | **γ-(Methylamino)-3-pyridinebutanoic acid** | PMI0008168 | 17270-48-3 | C10H14N2O2 | 37.81 | 195.11257 | 2.09 | 2.4 | -12.0 | 0.1 | 45.4 | 35.3 | -1.2 | 93.1 | na | | **RP-HESI(+)**, RP-APCI(+) | UCSD *in silico* MS^2^ |
| 94 | | **2-Furancarboxylic acid, 3-methyl** | PMI0003316 | 4412-96-8 | C6H6O3 | 37.72 | 127.03891 | 1.59 | 1.8 | -3.6 | 1.1 | 53.9 | 67.9 | -0.4 | 99.3 | 0.2 | | RP-HESI(+) | UCSD MS^2^ |
| 95 | | **5-{(3aS,5R,6R,6aS)-5-Hydroxy-6-[(1E,4S)-4-hydroxy-4-methyl-1-octen-1-yl]-1,3a,4,5,6,6a-hexahydro-2-pentalenyl}pentanoic acid** | CSID4943781 | 133906-74-8 | C22H36O4 | 36.66 | 365.26817 | 8.70 | 6.1 | -28.0 | 3.8 | 54.3 | 76.8 | -1.3 | 96.2 | na | | RP-HESI(+) | ChemIDplus *in silico* MS^2^ |
| 96 | | **Pyridine, 3-[2,5-dihydro-1-[(5-methyl-2furanyl)methyl]-1H-pyrrol-2-yl]** | PMI0008244 | 78210-45-4 | C15H16N2O | 36.60 | 241.13307 | 6.30 | 2.6 | -10.1 | 1.6 | 43.8 | 32.8 | -1.9 | 88.8 | na | | RP-HESI(+) | UCSD *in silico* MS^2^ |
| 97 | | **1,4-Benzenediamine, N-phenyl** | PMI0004952 | 101-54-2 | C12H12N2 | 36.25 | 185.10706 | 6.35 | 2.7 | -10.3 | 1.3 | 40.3 | 29.8 | -1.4 | 73.5 | na | | RP-HESI(+) | NIST MS/MS |
| 98 | | **Arachidic acid** | PMI0000329 | 506-30-9 | C20H40O2 | 35.76 | 311.29617 | 11.48 | 3.3 | -12.9 | 9.3 | 62.3 | 90.3 | 2.0 | 97.3 | 0.1 | | RP-HESI(-) | UCSD MS^2^ |
| 99 | | **4,6-Dihydroxy-20-nor-2,7-cembradien-12-one** | PMI0008379 | 119613-98-8 | C19H32O3 | 35.63 | 326.26838 | 9.32 | 3.2 | -21.7 | 3.6 | 51.6 | 63.7 | -1.9 | 96.7 | na | | RP-HESI(+) | UCSD *in silico* MS^2^ |
| 100 | | **α-Tocopherol** | PMI0006937 | 59-02-9 | C29H50O2 | 33.57 | 431.38720 | 13.31 | 4.8 | -21.5 | 11.9 | 74.0 | 91.3 | -2.7 | 92.5 | 0.0 | | **RP-HESI(+)**, RP-APCI(+), RP-HESI(-) | UCSD MS^2^ |
| 101 | | **Pyrrolo[1,2-a]pyrazine-1,4-dione, hexahydro-3propyl-, (3S-trans)** | PMI0008177 | 26626-89-1 | C10H16N2O2 | 33.50 | 197.12815 | 2.83 | 2.7 | -15.8 | -1.2 | 50.5 | 51.9 | -1.5 | 97.3 | -0.2 | | **RP-HESI(+)**, RP-APCI(+) | UCSD MS^2^ |
| 102 | | **N-(4-Hydroxyphenyl)-2-isopropyladenosine** | CSID114003 | 84930-20-1 | C19H23N5O5 | 33.45 | 419.20371 | 6.62 | 3.1 | -34.7 | 0.7 | 40.2 | 4.6 | -0.1 | 96.5 | na | | RP-HESI(+) | ChemIDplus *in silico* MS^2^ |
| 103 | | **(3β,5α)-Stigmasta-8,14,24(28)-trien-3-ol** | PMI0006674 | 34350-85-1 | C29H46O | 33.45 | 411.36184 | 13.02 | 3.6 | -28.4 | 9.9 | 45.6 | 35.2 | -0.7 | 93.8 | na | | RP-HESI(+) | UCSD *in silico* MS^2^ |
| 104 | | **3-Pyridinebutanol, d-amino** | PMI0008120 | 70898-36-1 | C9H14N2O | 33.13 | 167.11760 | 4.79 | 2.1 | -9.8 | -0.6 | 43.6 | 29.9 | -1.7 | 90.2 | na | | RP-HESI(+) | UCSD *in silico* MS^2^ |
| 105 | | **1H-Purine-2,6-dione, 8-(cyclohexylamino)-7-heptyl-3,7-dihydro-3-methyl-** | PMI0011812 | 106939-19-9 | C19H31N5O2 | 32.30 | 379.28151 | 10.12 | 5.2 | 0.0 | 0.0 | 39.5 | 0.6 | -0.3 | 97.0 | na | | RP-HESI(+) | UCSD *in silico* MS^2^ |
| 106 | | **3-Buten-2-one, 4-(3-hydroxy-2,6,6-trimethyl-1cyclohexen-1-yl)-, (E)** | PMI0008201 | 14398-34-6 | C13H20O2 | 32.21 | 209.15318 | 5.55 | 3.0 | -11.7 | 1.9 | 55.4 | 77.6 | -2.1 | 96.8 | -0.5 | | **RP-HESI(+)**, RP-APCI(+) | UCSD MS^2^ |
| 107 | | **Cyclo (Pro-Leu)** | PMI0007082 | 2873-36-1 | C11H18N2O2 | 31.77 | 211.14366 | 3.94 | 1.9 | -15.6 | -0.8 | 50.1 | 54.9 | -2.1 | 97.2 | na | | **RP-HESI(+)**, RP-APCI(+) | UCSD *in silico* MS^2^ |
| 108 | | **Isolinderanolide** | HMDB38105 | 139559-06-1 | C21H36O3 | 31.56 | 337.27260 | 10.39 | 5.6 | -24.4 | 7.7 | 39.9 | 5.3 | -3.3 | 98.3 | na | | RP-HESI(+) | HMDB *in silico* MS^2^ |
| 109 | | **Farnesylacetone** | PMI0009656 | 762-29-8 | C18H30O | 30.94 | 263.23644 | 9.64 | 3.9 | -11.6 | 6.2 | 63.1 | 76.2 | -1.9 | 97.6 | 0.0 | | **RP-HESI(+)**, RP-APCI(+) | UCSD MS^2^ |
| 110 | | **Benzidine** | PMI0005706 | 92-87-5 | C12H12N2 | 30.24 | 185.10707 | 5.96 | 2.4 | -10.6 | 1.6 | 48.2 | 48.4 | -1.4 | 94.1 | na | | **RP-HESI(+)**, RP-APCI(+) | UCSD *in silico* MS^2^ |
| 111 | | **Lignoceric acid** | PMI0005933 | 557-59-5 | C24H48O2 | 30.07 | 367.35877 | 13.24 | 3.5 | -15.2 | 11.4 | 52.1 | 0.0 | 1.7 | 96.2 | 0.0 | | RP-HESI(-) | UCSD MS^2^ |
| 112 | | **4-Methylacetophenone** | PMI0003633 | 122-00-9 | C9H10O | 29.60 | 152.10671 | 5.73 | 2.6 | -1.7 | 2.1 | 56.7 | 0.0 | -2.1 | 98.6 | 0.0 | | RP-HESI(+) | UCSD MS^2^ |
| 113 | | **Octacosanoic acid** | PMI0006014 | 506-48-9 | C28H56O2 | 29.45 | 423.42134 | 14.90 | 3.6 | -17.2 | 13.5 | 49.7 | 4.0 | 1.4 | 95.0 | -0.1 | | RP-HESI(-) | UCSD MS^2^ |
| 114 | | **N'-Acetylanatabine** | PMI0006569 | 61892-64-6 | C12H14N2O | 29.17 | 203.11746 | 3.90 | 2.2 | -11.1 | 0.5 | 50.0 | 55.2 | -2.1 | 97.3 | na | | **RP-HESI(+),** RP-APCI(+), HILIC-HESI(+) | UCSD *in silico* MS^2^ |
| 115 | | **Myristic acid** | PMI0000161 | 544-63-8 | C14H28O2 | 28.89 | 227.20198 | 9.07 | 5.7 | -8.9 | 6.1 | 57.2 | 54.7 | 1.4 | 97.6 | 0.1 | | RP-HESI(-) | UCSD MS^2^ |
| 116 | | **2-Methyl-3-(2-methylphenyl)-6-phenyl-4(3H)-pyrimidinone** | CSID107643 | 87356-71-6 | C18H16N2O | 28.66 | 277.13321 | 7.52 | 3.3 | -15.9 | 3.4 | 45.1 | 31.3 | -1.2 | 95.5 | na | | RP-HESI(+) | ChemIDplus *in silico* MS^2^ |
| 117 | | **Phytuberol** | PMI0000007 | 56857-64-8 | C15H24O3 | 27.77 | 253.17931 | 5.22 | 2.2 | -13.1 | 1.5 | 47.4 | 41.8 | -2.0 | 97.4 | na | | **RP-HESI(+)**, RP-APCI(+) | UCSD *in silico* MS^2^ |
| 118 | | **16-Hydroxy-9-hexadecenoic acid** | PMI0011844 | 17278-80-7 | C16H30O3 | 27.08 | 269.21267 | 7.79 | 4.1 | -16.5 | 4.6 | 41.3 | 10.7 | 1.7 | 97.6 | na | | RP-HESI(-) | UCSD *in silico* MS^2^ |
| 119 | | **3-Hydroxypalmitic acid** | PMI0011795 | 1111092-41-1 | C16H32O3 | 26.86 | 271.22835 | 8.57 | 3.6 | -17.3 | 5.2 | 51.1 | 49.9 | 1.8 | 97.3 | -0.3 | | RP-HESI(-) | UCSD MS^2^ |
| 120 | | **Pyridinol, methyl** | PMI0002811 | 1003-56-1 | C6H7NO | 26.11 | 110.06029 | 2.53 | 3.3 | -7.2 | 0.0 | 50.2 | 52.6 | 2.2 | 93.4 | 0.1 | | **RP-HESI(+),** RP-APCI(+) | UCSD MS^2^ |
| 121 | | **o-Anisidin** | PMI0003229 | 90-04-0 | C7H9NO | 26.08 | 124.07568 | 4.13 | 2.1 | -2.4 | 1.1 | 60.4 | 50.2 | -0.1 | 98.2 | 0.0 | | **RP-HESI(+),** RP-APCI(+) | UCSD MS^2^ |
| 122 | | **(S)-1-(1-oxobutyl)-2-(3-pyridinyl)-Pyrrolidine** | PMI0006586 | 69730-91-2 | C13H18N2O | 25.59 | 219.14881 | 5.42 | 6.4 | -12.6 | 1.3 | 44.1 | 36.5 | -1.7 | 86.2 | na | | RP-HESI(+) | UCSD *in silico* MS^2^ |
| 123 | | **Ethyl nicotinate** | PMI0000758 | 614-18-6 | C8H9NO2 | 25.47 | 152.07049 | 1.01 | 5.7 | -2.4 | 1.4 | 40.1 | 12.3 | -0.7 | 88.9 | na | | RP-APCI(+) | UCSD *in silico* MS^2^ |
| 124 | | **5,9,13,17-Nonadecatetraen-2-one, 6,10,14,18tetramethyl** | PMI0007520 | 6809-52-5 | C23H38O | 25.40 | 348.32604 | 10.94 | 2.9 | -16.8 | 8.2 | 67.6 | 88.0 | -1.5 | 96.5 | 0.0 | | RP-HESI(+) | UCSD MS^2^ |
| 125 | | **5-[(2S)-1-Methylpyrrolidin-2-yl]-N-phenylpyridin-2-amine** | PMI0001479 | na | C16H19N3 | 25.33 | 254.16466 | 5.09 | 1.9 | -12.4 | 2.5 | 50.5 | 57.7 | -2.0 | 97.1 | na | | **RP-HESI(+),** RP-APCI(+) | UCSD *in silico* MS^2^ |
| 126 | | **N-Acetylbenzidine** | CSID17740 | 3366-61-8 | C14H14N2O | 25.20 | 227.11742 | 5.76 | 2.7 | -18.7 | 1.9 | 43.9 | 30.7 | -2.1 | 91.4 | na | | RP-HESI(+) | FDA *in silico* MS^2^ |
| 127 | | **Acetamide, N-(2-phenylethyl)** | PMI0004520 | 877-95-2 | C10H13NO | 25.17 | 164.10685 | 4.75 | 2.0 | -9.8 | 1.1 | 35.1 | 13.4 | -0.9 | 63.2 | na | | RP-HESI(+) | UCSD *in silico* MS^2^ |
| 128 | | **7-Dehydrocholesterol** | PMI0011573 | 434-16-2 | C27H44O | 24.98 | 385.34591 | 12.88 | 2.4 | -25.5 | 9.5 | 39.0 | 1.4 | -1.5 | 95.3 | na | | RP-HESI(+) | NIST MS/MS |
| 129 | | **2H-Pyrrol-2-one, 4-ethyl-1,5-dihydro-3-methyl** | PMI0003308 | 766-45-0 | C7H11NO | 24.76 | 126.09131 | 3.72 | 3.4 | -5.5 | 0.5 | 50.3 | 52.9 | -0.3 | 99.0 | na | | **RP-HESI(+)**, RP-APCI(+) | UCSD *in silico* MS^2^ |
| 130 | | **4-(3-{(E)-[Amino(nitroamino)methylene]amino}propyl)-2-phenyl-1,3-oxazol-5-yl pivalate** | CSID159794 | 71162-59-9 | C18H23N5O5 | 24.64 | 407.20386 | 7.03 | 3.7 | -28.9 | 2.8 | 40.2 | 4.7 | 0.3 | 96.7 | na | | RP-HESI(+) | ChemIDplus *in silico* MS^2^ |
| 131 | | **Adenine** | PMI0007238 | 73-24-5 | C5H5N5 | 23.92 | 136.06157 | 1.18 | 3.0 | -26.6 | 0.0 | 62.7 | 68.0 | -1.5 | 93.0 | 0.0 | | **RP-HESI(+)**, RP-APCI(+), RP-HESI(-), HILIC-HESI(+) | UCSD MS^2^ |
| 132 | | **9H-Pyrido[3,4-b]indole, 1-(1-propenyl)** | PMI0005263 | 78210-55-6 | C14H12N2 | 23.87 | 209.10701 | 6.96 | 2.2 | -14.5 | 3.9 | 47.5 | 53.7 | -1.5 | 85.8 | na | | **RP-HESI(+)**, HILIC-HESI(+) | UCSD *in silico* MS^2^ |
| 133 | | **Isoquinoline, 3-methyl** | PMI0003968 | 1125-80-0 | C10H9N | 23.69 | 161.10705 | 2.39 | 3.8 | -3.1 | 2.4 | 45.7 | 32.3 | -1.4 | 97.9 | na | | RP-HESI(+) | UCSD *in silico* MS^2^ |
| 134 | | **Shikimic acid** | PMI0006517 | 138-59-0 | C7H10O5 | 23.54 | 173.04553 | 0.95 | 3.6 | -16.9 | -0.9 | 56.0 | 77.8 | -0.1 | 99.6 | 0.1 | | RP-HESI(-) | UCSD MS^2^ |
| 135 | | **α-Nicotyrine** | PMI0004444 | 525-75-7 | C10H10N2 | 23.49 | 159.09138 | 5.57 | 2.6 | -4.6 | 1.8 | 56.6 | 75.9 | -1.9 | 89.3 | -0.1 | | **RP-HESI(+)**, RP-APCI(+), RP-HESI(-), HILIC-HESI(+) | UCSD MS^2^ |
| 136 | | **2,7-Cembradiene-4,6,11,12-tetrol** | PMI0005886 | na | C20H36O4 | 22.71 | 358.29445 | 9.63 | 3.7 | -24.0 | 3.5 | 46.4 | 40.9 | -2.2 | 93.5 | na | | RP-HESI(+) | UCSD *in silico* MS^2^ |
| 137 | | **2-Propenamide, 3-(4-hydroxyphenyl)** | PMI0004515 | 59576-98-6 | C9H9NO2 | 22.57 | 181.09679 | 2.41 | 3.1 | -15.7 | 0.8 | 46.1 | 35.3 | -2.2 | 98.0 | na | | RP-HESI(+) | UCSD *in silico* MS^2^ |
| 138 | | **3-Hydroxysolavetivone** | PMI0008267 | 62623-88-5 | C15H22O2 | 22.43 | 235.16880 | 6.03 | 3.0 | -14.2 | 2.7 | 54.0 | 75.8 | -1.9 | 96.6 | na | | **RP-HESI(+)**, RP-APCI(+) | UCSD *in silico* MS^2^ |
| 139 | | **Palmitoleic acid** | PMI0000328 | 373-49-9 | C16H30O2 | 22.01 | 253.21769 | 9.25 | 3.6 | -12.8 | 6.6 | 52.7 | 54.1 | 1.5 | 97.3 | 0.2 | | RP-HESI(-) | UCSD MS^2^ |
| 140 | | **Cerebronic acid** | PMI0011805 | 544-57-0 | C24H48O3 | 21.77 | 383.35388 | 11.04 | 2.7 | -27.1 | 10.5 | 65.1 | 96.7 | 2.1 | 95.6 | -0.1 | | RP-HESI(-) | UCSD MS^2^ |
| 141 | | **3-Methyl-tyrosine** | CSID140377 | 17028-03-4 | C10H13NO3 | 21.68 | 213.12293 | 2.31 | 2.8 | -13.7 | 0.8 | 50.0 | 54.7 | -2.2 | 97.8 | na | | RP-HESI(+) | ChemIDplus *in silico* MS^2^ |
| 142 | | **Nicotine N-oxide** | PMI0006905 | 2820-55-5 | C10H14N2O | 21.65 | 179.11757 | 2.49 | 2.0 | -9.9 | -1.2 | 48.6 | 42.1 | -1.8 | 97.2 | -0.2 | | **RP-HESI(+)**, RP-APCI(+) | UCSD MS^2^ |
| 143 | | **β-D-Glucopyranose, 6-acetate 2,3,4-tris((+)-3methylpentanoate)** | PMI0008497 | 28977-67-5 | C26H44O10 | 21.64 | 534.32713 | 9.00 | 8.2 | -32.3 | 6.2 | 41.0 | 7.4 | -0.3 | 98.0 | na | | RP-HESI(+) | UCSD *in silico* MS^2^ |
| 144 | | **Heptadecanoic acid** | PMI0000165 | 506-12-7 | C17H34O2 | 21.60 | 269.24904 | 10.17 | 5.1 | -8.1 | 7.7 | 61.8 | 85.4 | 1.6 | 97.7 | 0.1 | | RP-HESI(-) | UCSD MS^2^ |
| 145 | | **3-(1-Pyrrolidinyl)phenol** | CSID105150 | 25912-16-7 | C10H13NO | 21.36 | 181.13319 | 5.35 | 3.3 | -9.0 | 1.6 | 43.0 | 28.2 | -2.2 | 89.5 | na | | RP-HESI(+) | ChemIDplus *in silico* MS^2^ |
| 146 | | **Moroctic acid** | PMI0011796 | 20290-75-9 | C18H28O2 | 21.28 | 275.20217 | 8.93 | 4.4 | -14.2 | 5.9 | 63.3 | 50.5 | 1.9 | 97.2 | 0.0 | | RP-HESI(-) | UCSD MS^2^ |
| 147 | | **(3aR,5R,7R,7aS)-7-(Cyclopropylmethoxy)-5-methoxy-N,N,2,2-tetramethylhexahydro-1,3-benzodioxole-5-carboxamide** | PMI0001739 | na | C17H29NO5 | 21.04 | 345.23979 | 8.18 | 3.5 | -15.7 | 1.5 | 39.0 | 4.9 | 4.3 | 95.1 | na | | RP-HESI(+) | UCSD *in silico* MS^2^ |
| 148 | | **1H-Pyrazole, 4-methyl-3-phenyl-** | PMI0009199 | 13808-62-3 | C10H10N2 | 20.70 | 159.09143 | 5.33 | 3.8 | -7.6 | 2.7 | 48.1 | 52.9 | -1.5 | 89.3 | na | | RP-HESI(+) | UCSD *in silico* MS^2^ |
| 149 | | **5-Methylnicotine** | PMI0009837 | 82111-06-6 | C11H16N2 | 20.09 | 177.13834 | 5.08 | 2.4 | -4.7 | 1.2 | 44.5 | 26.8 | -1.6 | 97.9 | na | | RP-HESI(+) | UCSD *in silico* MS^2^ |
| 150 | | **2,3,5-Trimethyl-5,6-dihydropyrazine** | PMI0009743 | 65826-70-2 | C7H12N2 | 19.93 | 125.10728 | 1.95 | 3.1 | 0.0 | -1.1 | 44.5 | 24.6 | -0.3 | 98.5 | na | | **RP-HESI(+)**, HILIC-HESI(+) | UCSD *in silico* MS^2^ |
| 151 | | **2-Methyl-3-propyl-5,6-dihydropyrazine** | PMI0009741 | 15986-94-4 | C8H14N2 | 19.48 | 139.12274 | 2.77 | 3.6 | -1.5 | -0.5 | 44.2 | 24.9 | -1.7 | 98.2 | na | | **RP-HESI(+)**, HILIC-HESI(+) | UCSD *in silico* MS^2^ |
| 152 | | **6Pyrimidinol, 4-methyl-2-propyl** | PMI0008098 | 16858-16-5 | C8H12N2O | 19.36 | 153.10201 | 3.80 | 2.6 | -3.2 | 0.9 | 44.3 | 32.0 | -1.5 | 91.2 | na | | RP-HESI(+) | UCSD *in silico* MS^2^ |
| 153 | | **(Z,Z,Z)-18-hydroxy-9,12,15-Octadecatrienoic acid** | PMI0006643 | 51327-73-2 | C18H30O3 | 19.35 | 293.21284 | 7.83 | 3.8 | -20.4 | 4.7 | 49.6 | 53.8 | 2.1 | 96.5 | na | | RP-HESI(-) | UCSD *in silico* MS^2^ |
| 154 | | **Niacine** | PMI0000158 | 59-67-6 | C6H5NO2 | 19.03 | 124.03930 | 1.04 | 2.2 | -7.1 | 0.1 | 53.5 | 64.3 | 0.0 | 98.6 | 0.1 | | **RP-HESI(+),** RP-APCI(+) | UCSD MS^2^ |
| 155 | | **1-Methyl-2-((methyl(prop-2-ynyl)amino)methyl)-1H-indol-5-ol** | PMI0012129 | 130081-94-6 | C14H16N2O | 18.96 | 229.13321 | 4.85 | 1.8 | -14.6 | 2.1 | 48.1 | 47.2 | -1.4 | 94.9 | na | | **RP-HESI(+)**, RP-HESI(-) | UCSD *in silico* MS^2^ |
| 156 | | **Cyclo (Phe-Pro)** | PMI0008798 | 3705-26-8 | C14H16N2O2 | 18.92 | 245.12802 | 4.31 | 2.5 | -22.5 | -0.6 | 56.6 | 71.1 | -1.8 | 95.5 | 0.1 | | **RP-HESI(+),** RP-APCI(+) | UCSD MS^2^ |
| 157 | | **1H-Imidazole, 2-ethyl-4-methyl** | PMI0002855 | 931-36-2 | C6H10N2 | 18.89 | 111.09188 | 1.29 | 3.2 | -5.8 | 0.6 | 48.2 | 46.0 | 1.8 | 93.6 | -0.1 | | **RP-HESI(+)**, HILIC-HESI(+) | UCSD MS^2^ |
| 158 | | **Megastigmatrienone** | PMI0007095 | 13215-88-8 | C13H18O | 18.43 | 191.14286 | 7.45 | 5.7 | -6.1 | 3.4 | 50.1 | 52.4 | -0.3 | 98.5 | na | | RP-HESI(+), **RP-APCI(+)** | UCSD *in silico* MS^2^ |
| 159 | | **Tricosanoic acid** | PMI0005915 | 2433-96-7 | C23H46O2 | 18.33 | 353.34321 | 12.81 | 3.2 | -14.6 | 10.9 | 55.0 | 10.6 | 2.0 | 96.4 | 0.0 | | RP-HESI(-) | UCSD MS^2^ |
| 160 | | **Cembrene** | PMI0005665 | 101159-08-4 | C20H32 | 17.87 | 273.25699 | 10.05 | 2.9 | -10.3 | 9.1 | 52.8 | 69.3 | -2.5 | 97.5 | na | | RP-HESI(+) | UCSD *in silico* MS^2^ |
| 161 | | **2-Hydroxypyridine** | PMI0009744 | 72762-00-6 | C5H5NO | 17.79 | 113.07109 | 1.24 | 2.7 | -7.0 | -0.6 | 50.6 | 60.3 | 1.6 | 94.7 | na | | **RP-HESI(+)**, HILIC-HESI(+) | UCSD *in silico* MS^2^ |
| 162 | | **β-Sitosterol** | PMI0001972 | 83-47-6 | C29H50O | 17.70 | 397.38271 | 13.91 | 8.3 | -26.4 | 10.7 | 58.1 | 78.6 | -0.4 | 95.2 | 0.3 | | RP-APCI(+) | UCSD MS^2^ |
| 163 | | **Piperidine, 1-[(5-methoxyindol-2-yl)carbonyl]-** | PMI0012132 | 22930-55-8 | C15H18N2O2 | 17.50 | 259.14374 | 6.34 | 4.0 | -19.7 | 0.9 | 43.5 | 30.1 | -1.4 | 89.0 | na | | **RP-HESI(+)**, RP-APCI(+) | UCSD *in silico* MS^2^ |
| 164 | | **2-Methyl-5-pyridinol** | PMI0002804 | 1121-78-4 | C6H7NO | 17.30 | 110.06027 | 1.67 | 3.2 | -7.1 | 1.1 | 49.1 | 48.9 | 2.1 | 93.3 | 0.1 | | **RP-HESI(+)**, RP-APCI(+), HILIC-HESI(+) | UCSD MS^2^ |
| 165 | | **N-Ethylnorcotinine** | PMI0009836 | 359435-41-9 | C11H14N2O | 17.26 | 191.11773 | 3.26 | 5.6 | -10.9 | 0.3 | 46.2 | 31.1 | -0.8 | 90.6 | -0.1 | | RP-HESI(+), **RP-APCI(+)** | UCSD MS^2^ |
| 166 | | **Campesterol** | PMI0000318 | 474-62-4 | C28H48O | 17.07 | 383.36691 | 13.58 | 7.4 | -25.1 | 10.2 | 59.0 | 83.9 | -0.8 | 95.4 | 0.3 | | RP-APCI(+) | UCSD MS^2^ |
| 167 | | **1-(3-pyridinyl)-1,4-Butanediol** | PMI0011775 | 76014-83-0 | C9H13NO2 | 16.32 | 185.12838 | 3.16 | 4.8 | -11.0 | -0.8 | 46.9 | 40.3 | -0.5 | 94.9 | na | | **RP-HESI(+)**, HILIC-HESI(+) | UCSD *in silico* MS^2^ |
| 168 | | **2-Acetylpyridine** | PMI0003159 | 1122-62-9 | C7H7NO | 16.26 | 139.08637 | 3.01 | 3.2 | -0.7 | 0.9 | 42.6 | 22.4 | -1.8 | 92.6 | na | | RP-HESI(+) | UCSD *in silico* MS^2^ |
| 169 | | **Isoraimonol** | PMI0007970 | 82458-63-7 | C20H32O | 15.99 | 289.25227 | 9.64 | 8.0 | -14.9 | 6.8 | 55.7 | 82.4 | -1.1 | 97.3 | na | | RP-HESI(+), **RP-APCI(+)** | UCSD *in silico* MS^2^ |
| 170 | | **Triacontanoic acid** | PMI0006042 | 506-50-3 | C30H60O2 | 15.83 | 451.45294 | 15.80 | 4.2 | -18.1 | 14.6 | 61.2 | 94.9 | 2.0 | 94.6 | 0.3 | | RP-HESI(-) | UCSD MS^2^ |
| 171 | | **4-Hydroxy-3-(6-hydroxy-5-methyl-3,6-dihydro-2H-pyran-2-yl)-7-methoxy-2,3,8-trimethyl-2,3-dihydronaphtho[1,2-b]furan-6,9-dione** | CSID116471 | 134985-02-7 | C22H24O7 | 15.72 | 401.15912 | 5.01 | 3.9 | -39.1 | 2.6 | 52.3 | 66.3 | -0.9 | 96.2 | na | | RP-HESI(+) | ChemIDplus *in silico* MS^2^ |
| 172 | | **10,12,15-Octadecatrienoic acid, 9-hydroxy** | PMI0012135 | 89886-42-0 | C18H30O3 | 15.34 | 293.21284 | 8.13 | 2.9 | -19.4 | 4.8 | 50.7 | 59.3 | 2.2 | 96.7 | na | | RP-HESI(-) | NIST MS/MS |
| 173 | | **Norcotinine** | PMI0006492 | 5980-06-3 | C9H10N2O | 15.29 | 163.08632 | 2.29 | 2.5 | -13.4 | -1.0 | 51.0 | 47.8 | -1.7 | 98.6 | -0.1 | | **RP-HESI(+)**, RP-APCI(+), HILIC-HESI(+) | UCSD MS^2^ |
| 174 | | **1-Methyl-4-phenyl-5,6-dihydro-2-pyridone** | CSID155585 | 94515-22-7 | C12H13NO | 14.75 | 188.10686 | 5.92 | 5.1 | -11.0 | 1.9 | 45.2 | 29.9 | -0.7 | 96.9 | na | | RP-APCI(+) | ChemIDplus *in silico* MS^2^ |
| 175 | | **Loliolide** | PMI0007137 | 38274-00-9 | C11H16O3 | 14.71 | 197.11688 | 4.23 | 2.9 | -13.0 | 0.6 | 65.4 | 95.9 | -1.7 | 97.9 | 0.0 | | **RP-HESI(+)**, RP-APCI(+) | UCSD MS^2^ |
| 176 | | **Vernolic acid** | PMI0011821 | 503-07-1 | C18H32O3 | 14.70 | 295.22834 | 8.12 | 3.5 | -15.3 | 5.9 | 63.1 | 70.2 | 1.6 | 96.6 | 0.0 | | RP-HESI(-) | UCSD MS^2^ |
| 177 | | **Glycidol oleate** | CSID4510739 | 5431-33-4 | C21H38O3 | 14.68 | 339.28892 | 10.95 | 4.3 | -15.8 | 8.2 | 41.3 | 12.4 | -1.4 | 95.7 | na | | RP-HESI(+) | ChemIDplus *in silico* MS^2^ |
| 178 | | **1H-Pyrazole, 3,4-dimethyl** | PMI0002550 | 2820-37-3 | C5H8N2 | 14.67 | 97.07642 | 1.17 | 3.6 | -1.8 | 1.2 | 42.7 | 23.8 | 4.1 | 94.6 | na | | RP-HESI(+) | UCSD *in silico* MS^2^ |
| 179 | | **(S)-1-acetyl-2-(3-pyridinyl)-Piperidine** | PMI0006574 | 3350-86-5 | C12H16N2O | 14.51 | 205.13317 | 4.18 | 2.5 | -12.0 | 0.8 | 49.6 | 40.5 | -1.8 | 96.5 | 0.1 | | **RP-HESI(+)**, RP-APCI(+), HILIC-HESI(+) | UCSD MS^2^ |
| 180 | | **2(3H)-Furanone, dihydro-5-(3-pyridinyl)** | PMI0004516 | 20971-79-3 | C9H9NO2 | 14.42 | 164.07028 | 2.44 | 2.3 | -10.8 | -0.6 | 42.5 | 17.2 | -2.0 | 97.5 | na | | RP-HESI(+) | UCSD *in silico* MS^2^ |
| 181 | | **o-Toluic acid** | PMI0003710 | 118-90-1 | C8H8O2 | 14.29 | 135.04467 | 2.94 | 2.6 | -5.1 | 2.4 | 46.2 | 27.1 | -3.6 | 98.7 | 0.1 | | RP-HESI(-) | UCSD MS^2^ |
| 182 | | **Cerotinic acid** | PMI0005981 | 506-46-7 | C26H52O2 | 14.22 | 395.39002 | 14.08 | 4.9 | -16.2 | 12.5 | 48.5 | 5.2 | 1.4 | 95.5 | 0.1 | | RP-HESI(-) | UCSD MS^2^ |
| 183 | | **Methyl-pyroglutamate** | PMI0000117 | 4931-66-2 | C6H9NO3 | 14.19 | 144.06533 | 1.55 | 2.5 | -7.1 | -1.9 | 63.0 | 71.2 | -1.3 | 92.8 | 0.0 | | RP-HESI(+) | UCSD MS^2^ |
| 184 | | **1-Octylimidazole** | PMI0011806 | 21252-69-7 | C11H20N2 | 14.17 | 181.16956 | 5.31 | 3.1 | -6.3 | 3.5 | 44.3 | 35.9 | -2.0 | 88.2 | na | | **RP-HESI(+)**, HILIC-HESI(+) | UCSD *in silico* MS^2^ |
| 185 | | **Anatalline** | PMI0005384 | 1189431-36-4 | C15H17N3 | 13.67 | 240.14888 | 3.44 | 5.4 | -14.5 | 0.8 | 55.8 | 82.7 | -2.7 | 90.9 | -0.2 | | **RP-HESI(+)**, HILIC-HESI(+) | UCSD MS^2^ |
| 186 | | **Harmalol** | PMI0011345 | 525-57-5 | C12H12N2O | 13.26 | 218.12843 | 2.99 | 2.6 | -16.2 | 0.4 | 38.8 | 0.0 | -1.8 | 96.0 | na | | **RP-HESI(+)**, RP-APCI(+) | NIST MS/MS |
| 187 | | **Geranyl benzoate** | PMI0009496 | 94-48-4 | C17H22O2 | 13.19 | 259.16886 | 8.14 | 2.9 | -10.9 | 6.1 | 42.4 | 17.3 | -1.5 | 96.3 | na | | RP-HESI(+) | UCSD *in silico* MS^2^ |
| 188 | | **2-(4-Hydroxyphenyl)ethyl octacosanoate** | CSID156828 | 123690-76-6 | C36H64O3 | 12.80 | 543.47920 | 16.19 | 5.3 | -36.0 | 15.4 | 51.8 | 63.2 | 1.7 | 97.8 | na | | RP-HESI(-) | ChemIDplus *in silico* MS^2^ |
| 189 | | **N-[(3S)-1-(1-Amino-1-oxo-3-phenyl-2-propanyl)-2,5-dioxo-3-pyrrolidinyl]-N~2~-{[(2-methyl-2-propanyl)oxy]carbonyl}-L-leucinamide** | CSID169219 | 114646-30-9 | C24H34N4O6 | 12.54 | 492.28018 | 8.10 | 5.1 | na | 1.9 | 38.2 | 1.0 | -3.1 | 93.7 | na | | RP-HESI(+) | ChemIDplus *in silico* MS^2^ |
| 190 | | **Proline** | PMI0011689 | 609-36-9 | C5H9NO2 | 12.53 | 98.06056 | 1.51 | 4.2 | -5.1 | -0.6 | 44.2 | 32.3 | 4.5 | 94.1 | na | | RP-APCI(+) | UCSD *in silico* MS^2^ |
| 191 | | **Cyclo (Pro-Thr)** | PMI0008803 | na | C9H14N2O3 | 12.43 | 199.10755 | 1.55 | 6.7 | -25.9 | -3.0 | 45.4 | 38.6 | -0.9 | 89.5 | na | | RP-APCI(+) | UCSD *in silico* MS^2^ |
| 192 | | **trans-Nicotine-1'-oxide** | PMI0006906 | 51095-86-4 | C10H14N2O | 12.39 | 179.11754 | 1.35 | 3.7 | na | na | 52.8 | 63.8 | -2.0 | 97.9 | -0.1 | | RP-HESI(+) | UCSD MS^2^ |
| 193 | | **2-Methylnicotinamide** | PMI0003712 | 58539-65-4 | C7H8N2O | 12.35 | 137.07071 | 1.78 | 1.6 | -5.1 | 0.4 | 44.4 | 31.9 | -1.7 | 92.1 | na | | **RP-HESI(+)**, RP-APCI(+) | UCSD *in silico* MS^2^ |
| 194 | | **2-[1'-Methyl-1,1'-bi(cyclohexan)-3-en-4-yl]propanoic acid** | CSID179201 | 28745-04-2 | C16H26O2 | 12.05 | 249.18670 | 8.52 | 5.2 | -12.9 | 5.6 | 39.5 | 4.8 | 2.8 | 96.2 | na | | RP-HESI(-) | ChemIDplus *in silico* MS^2^ |
| 195 | | **Pentacosanoic acid** | PMI0005961 | 506-38-7 | C25H50O2 | 11.97 | 381.37428 | 13.66 | 3.2 | -15.7 | 11.9 | 56.5 | 36.1 | 1.2 | 95.6 | 0.1 | | RP-HESI(-) | UCSD MS^2^ |
| 196 | | **Guaiacylacetone** | PMI0000283 | 2503-46-0 | C10H12O3 | 11.96 | 181.08558 | 3.50 | 1.9 | -7.7 | 0.4 | 55.1 | 63.2 | -1.9 | 73.2 | 0.0 | | **RP-HESI(+)**, RP-APCI(+) | UCSD MS^2^ |
| 197 | | **Catechol** | PMI0000457 | 120-80-9 | C6H6O2 | 11.76 | 111.04427 | 1.60 | 2.1 | -4.0 | 0.9 | 57.0 | 86.1 | 2.0 | 98.2 | -0.3 | | RP-HESI(+) | UCSD MS^2^ |
| 198 | | **Cyclo (Ser-Val)** | PMI0008808 | na | C8H14N2O3 | 11.14 | 169.09702 | 1.54 | 6.1 | -27.6 | -1.8 | 45.9 | 39.3 | -0.7 | 91.0 | na | | RP-APCI(+) | UCSD *in silico* MS^2^ |
| 199 | | **6-Hydroxynicotine** | PMI0006525 | 2055-13-2 | C10H14N2O | 11.13 | 179.11756 | 1.74 | 2.4 | -11.3 | -0.6 | 46.8 | 38.4 | -1.9 | 97.8 | na | | RP-HESI(+) | UCSD *in silico* MS^2^ |
| 200 | | **2H-Pyrrol-2-one, 1,5-dihydro-4,5-dimethyl-3-ethyl** | PMI0003857 | na | C8H13NO | 11.00 | 140.10674 | 4.56 | 3.9 | -5.5 | 1.0 | 46.8 | 37.0 | -1.8 | 99.2 | na | | RP-HESI(+) | UCSD *in silico* MS^2^ |
| 201 | | **5,6-Dimethyl-3-pyridinol** | PMI0003225 | 61893-00-3 | C7H9NO | 10.62 | 124.07571 | 3.14 | 2.6 | -8.6 | 1.6 | 56.0 | 72.6 | 0.1 | 99.3 | -0.1 | | **RP-HESI(+)**, RP-APCI(+), HILIC-HESI(+) | UCSD MS^2^ |
| 202 | | **Stigmasterol** | PMI0000319 | 83-48-7 | C29H48O | 10.43 | 395.36705 | 13.60 | 7.5 | -26.3 | 10.2 | 71.8 | 84.0 | -0.4 | 95.6 | 0.0 | | RP-HESI(+), **RP-APCI(+)** | UCSD MS^2^ |
| 203 | | **1-Methyl-2-[(2-propyn-1-ylamino)methyl]-1H-indol-5-ol** | PMI0011842 | 133681-83-1 | C13H14N2O | 10.21 | 215.11776 | 4.67 | 5.0 | -16.8 | 1.6 | 51.5 | 60.8 | -0.6 | 97.5 | na | | RP-APCI(+) | UCSD *in silico* MS^2^ |
| 204 | | **Phenylalanyl-N-{(2S)-3-methyl-2-[(2-oxo-3-piperidinyl)amino]butanoyl}-L-prolinamide** | CSID168955 | 107759-05-7 | C24H35N5O4 | 10.08 | 475.30292 | 9.38 | 6.5 | 0.0 | -0.9 | 40.3 | 3.8 | 0.4 | 98.0 | na | | RP-HESI(+) | ChemIDplus *in silico* MS^2^ |
| 205 | | **Heneicosanoic acid** | PMI0005836 | 2363-71-5 | C21H42O2 | 9.85 | 325.31180 | 11.92 | 2.1 | -13.5 | 9.8 | 65.6 | 96.4 | 1.8 | 97.5 | -0.1 | | RP-HESI(-) | UCSD MS^2^ |
| 206 | | **1-{5-[(2S)-1-Methylpyrrolidin-2-yl]pyridin-2-yl}-3-phenylurea** | PMI0000875 | na | C17H20N4O | 9.83 | 279.16026 | 5.03 | 6.3 | -11.5 | 1.5 | 46.7 | 37.0 | -0.6 | 97.1 | na | | RP-APCI(+) | UCSD *in silico* MS^2^ |
| 207 | | **10-Nonadecenoic acid** | PMI0011829 | 67228-95-9 | C19H36O2 | 9.78 | 295.26469 | 10.26 | 3.3 | -14.4 | 8.2 | 39.7 | 3.4 | 1.5 | 97.1 | na | | RP-HESI(-) | UCSD *in silico* MS^2^ |
| 208 | | **4-(2-Naphthylamino)phenol** | CSID6874 | 93-45-8 | C16H13NO | 9.67 | 253.13312 | 5.32 | 2.9 | -16.3 | 4.1 | 50.4 | 56.3 | -1.8 | 97.9 | na | | RP-HESI(+) | FDA *in silico* MS^2^ |
| 209 | | **Cyclo (Ser-Tyr)** | PMI0008629 | na | C12H14N2O4 | 9.45 | 251.10199 | 3.40 | 3.6 | 0.0 | 0.0 | 49.3 | 52.9 | -2.6 | 96.6 | na | | RP-HESI(+) | UCSD *in silico* MS^2^ |
| 210 | | **Guaiacol, 5-amino-** | PMI0011719 | 1687-53-2 | C7H9NO2 | 9.38 | 140.07048 | 1.34 | 4.9 | -6.9 | -0.1 | 46.1 | 31.1 | -0.9 | 98.6 | -0.4 | | RP-APCI(+) | UCSD MS^2^ |
| 211 | | **γ-Tocotrienol** | PMI0011738 | 14101-61-2 | C28H42O2 | 9.33 | 411.32554 | 13.76 | 4.7 | -25.7 | 10.3 | 42.7 | 18.7 | -0.5 | 95.2 | na | | RP-APCI(+) | NIST MS/MS |
| 212 | | **Tryptophan** | PMI0007921 | 6912-86-3 | C11H12N2O2 | 9.24 | 187.08645 | 5.37 | 7.0 | -18.6 | 1.0 | 46.0 | 42.6 | -0.7 | 88.0 | na | | RP-APCI(+) | UCSD *in silico* MS^2^ |
| 213 | | **Pyranone** | PMI0000228 | 28564-83-2 | C6H8O4 | 8.96 | 127.03906 | 1.50 | 4.6 | -7.7 | -0.4 | 74.5 | 92.1 | 0.6 | 99.2 | 0.0 | | RP-HESI(+), **RP-APCI(+)** | UCSD MS^2^ |
| 214 | | **Nonacosanoic acid** | PMI0006024 | 4250-38-8 | C29H58O2 | 8.85 | 437.43711 | 15.34 | 2.9 | -17.6 | 14.1 | 42.5 | 1.8 | 1.6 | 94.7 | 0.3 | | RP-HESI(-) | UCSD MS^2^ |
| 215 | | **1,4,7,10-Cyclotetradecatetraene, 1,7,11-trimethyl-4(1-methylethenyl)** | PMI0008318 | 101159-07-3 | C20H30 | 8.60 | 271.24169 | 9.55 | 8.9 | -11.0 | 8.6 | 52.3 | 66.0 | -1.3 | 96.9 | na | | RP-HESI(+), **RP-APCI(+)** | UCSD *in silico* MS^2^ |
| 216 | | **Phenylalanine** | PMI0004562 | 63-91-2 | C9H11NO2 | 8.56 | 148.07558 | 4.29 | 5.8 | -8.1 | 1.1 | 47.1 | 38.2 | -0.7 | 98.4 | na | | RP-APCI(+) | UCSD *in silico* MS^2^ |
| 217 | | **7-Cholesterol** | PMI0006700 | 6036-58-4 | C27H46O | 8.55 | 369.35138 | 13.22 | 6.8 | -24.1 | 9.8 | 58.6 | 81.0 | -0.5 | 95.4 | 0.3 | | RP-APCI(+) | UCSD MS^2^ |
| 218 | | **Indolelactic acid** | PMI0012051 | 1821-52-9 | C11H11NO3 | 8.42 | 223.10729 | 2.80 | 2.4 | -21.2 | 1.0 | 42.9 | 22.0 | -2.1 | 94.9 | na | | RP-HESI(+) | UCSD *in silico* MS^2^ |
| 219 | | **5,6-Dimethyl-8-quinolinamine** | CSID99156 | 68527-69-5 | C11H12N2 | 8.35 | 173.10726 | 6.29 | 4.6 | -9.7 | 2.8 | 45.4 | 49.7 | -0.4 | 77.7 | na | | RP-APCI(+) | ChemIDplus *in silico* MS^2^ |
| 220 | | **6-Pyrimidinol, 4,5-dimethyl** | PMI0003252 | 34916-78-4 | C6H8N2O | 8.32 | 125.07104 | 1.93 | 5.1 | -4.6 | -0.2 | 45.6 | 30.4 | 0.8 | 98.7 | na | | RP-APCI(+) | UCSD *in silico* MS^2^ |
| 221 | | **5-Methoxytryptophan** | PMI0011790 | 2504-22-5 | C12H14N2O3 | 8.32 | 217.09702 | 4.43 | 5.3 | -21.3 | 1.0 | 37.0 | 28.1 | -0.6 | 57.7 | na | | RP-APCI(+) | UCSD *in silico* MS^2^ |
| 222 | | **Huperzine A** | PMI0002138 | 102518-79-6 | C15H18N2O | 8.31 | 243.14901 | 7.66 | 7.8 | -22.1 | 0.7 | 42.5 | 18.1 | -0.7 | 95.5 | na | | RP-APCI(+) | UCSD *in silico* MS^2^ |
| 223 | | **(5S,6E,8E,10Z,12S,14Z)-5,12,20-Trihydroxy-6,8,10,14-icosatetraenoic acid** | CSID4942820 | 79008-24-5 | C20H32O5 | 8.03 | 351.21849 | 6.67 | 4.7 | -35.1 | 2.1 | 45.1 | 32.8 | 2.3 | 95.5 | na | | RP-HESI(-) | ChemIDplus *in silico* MS^2^ |
| 224 | | **2(3H)-Furanone, dihydro-5-(3-hydroxy-1-butenyl)-4-((4-(hydroxymethyl)phenyl)amino)-** | CSID4523192 | 144398-01-6 | C15H19NO4 | 7.95 | 295.16474 | 4.41 | 3.3 | -27.5 | -0.9 | 50.3 | 58.5 | -1.8 | 95.0 | na | | RP-HESI(+) | ChemIDplus *in silico* MS^2^ |
| 225 | | **6-(4-Hydroxyphenyl)tetrahydro-2H-pyran-2-one** | PMI0011880 | 89647-77-8 | C11H12O3 | 7.95 | 193.08563 | 3.57 | 6.6 | -15.3 | 0.7 | 46.6 | 36.8 | -1.5 | 97.9 | na | | RP-APCI(+) | UCSD *in silico* MS^2^ |
| 226 | | **Pyrazineethanol, 3-methyl** | PMI0008076 | 61892-92-0 | C7H10N2O | 7.68 | 139.08646 | 2.17 | 5.5 | -4.2 | -0.7 | 44.5 | 30.7 | -0.9 | 92.8 | na | | RP-APCI(+) | UCSD *in silico* MS^2^ |
| 227 | | **3-Aminoquinolin** | PMI0000381 | 580-17-6 | C9H8N2 | 7.10 | 145.07592 | 4.77 | 5.1 | -7.5 | 1.5 | 52.4 | 72.3 | -0.7 | 90.6 | na | | RP-HESI(+), **RP-APCI(+)** | NIST MS/MS |
| 228 | | **1-(2,3,4,9-Tetrahydro-1H-beta-carbolin-1-yl)acetone** | PMI0011850 | 69225-88-3 | C14H16N2O | 7.09 | 229.13337 | 5.38 | 5.7 | -14.1 | 1.0 | 47.8 | 47.2 | -0.8 | 92.6 | na | | RP-APCI(+) | UCSD *in silico* MS^2^ |
| 229 | | **2,3'-Bipyridine, 6-methyl** | PMI0004620 | 78210-78-3 | C11H10N2 | 6.98 | 171.09143 | 1.36 | 2.2 | -5.5 | 1.8 | 58.4 | 91.6 | -1.4 | 98.1 | 0.1 | | HILIC-HESI(+) | UCSD MS^2^ |
| 230 | | **2-Acetyl-4-methylpyridine** | PMI0003666 | 59576-26-0 | C8H9NO | 6.95 | 136.07549 | 4.51 | 2.4 | -2.5 | 1.3 | 50.1 | 29.1 | -1.4 | 98.0 | -0.1 | | **RP-HESI(+)**, RP-APCI(+) | UCSD MS^2^ |
| 231 | | **Harmol** | PMI0011375 | 487-03-6 | C12H10N2O | 6.85 | 199.08626 | 4.42 | 3.0 | -19.3 | 2.5 | 49.5 | 35.1 | -1.7 | 94.9 | -0.1 | | RP-HESI(+) | UCSD MS^2^ |
| 232 | | **Retinol** | PMI0007452 | 68-26-8 | C20H30O | 6.82 | 287.23666 | 9.67 | 7.1 | -18.7 | 6.8 | 61.5 | 64.1 | -1.0 | 97.1 | 0.0 | | RP-HESI(+), **RP-APCI(+)** | UCSD MS^2^ |
| 233 | | **Furaneol** | PMI0000503 | 3658-77-3 | C6H8O3 | 6.66 | 129.05454 | 2.43 | 2.4 | -3.4 | 0.3 | 57.8 | 68.1 | -0.6 | 98.5 | 0.0 | | **RP-HESI(+)**, RP-APCI(+) | UCSD MS^2^ |
| 234 | | **Ricinoleic acid** | PMI0011792 | 141-22-0 | C18H34O3 | 6.59 | 297.24398 | 8.36 | 4.0 | -18.3 | 5.7 | 53.4 | 29.1 | 1.5 | 96.9 | 0.0 | | RP-HESI(-) | UCSD MS^2^ |
| 235 | | **2-[4-(2H-Indazol-2-yl)phenyl]-1-propanol** | CSID2327477 | 81265-79-4 | C16H16N2O | 6.59 | 253.13329 | 7.39 | 5.8 | -9.3 | 3.5 | 43.6 | 41.0 | -1.0 | 78.2 | na | | RP-APCI(+) | ChemIDplus *in silico* MS^2^ |
| 236 | | **Sinapic acid** | PMI0002013 | 7362-37-0 | C11H12O5 | 6.58 | 225.07529 | 2.87 | 2.8 | -15.0 | 1.3 | 46.8 | 38.0 | -2.1 | 98.5 | na | | RP-HESI(+) | UCSD *in silico* MS^2^ |
| 237 | | **1,3-Naphthalenediol, 1,2,3,4,4a,5,6,7-octahydro-6[1-(hydroxymethyl)ethenyl]-4,4a-dimethyl-, (1a,3b,4b,4aa,6a)** | PMI0008294 | na | C15H24O3 | 6.55 | 235.16905 | 6.50 | 4.2 | -18.9 | 1.3 | 45.5 | 31.4 | -0.8 | 97.0 | na | | **RP-APCI(+)**, RP-HESI(-) | UCSD *in silico* MS^2^ |
| 238 | | **Methoxy-Eugenole** | PMI0000039 | 6627-88-9 | C11H14O3 | 6.53 | 195.10130 | 6.26 | 4.0 | -7.4 | 1.8 | 51.5 | 44.6 | -1.4 | 97.0 | -0.1 | | RP-HESI(+) | UCSD MS^2^ |
| 239 | | **2-Cyclopenten-1-one, 4-ethyl-2-methyl** | PMI0003263 | 71278-13-2 | C8H12O | 6.39 | 125.09620 | 4.96 | 4.9 | -0.5 | 1.6 | 47.0 | 36.4 | 0.9 | 99.4 | na | | RP-APCI(+) | UCSD *in silico* MS^2^ |
| 240 | | **N-Furfurylnornicotine** | PMI0006598 | 78210-85-2 | C14H16N2O | 6.31 | 229.13323 | 1.25 | 7.7 | -8.8 | 1.3 | 51.0 | 40.6 | -1.4 | 97.8 | 0.0 | | HILIC-HESI(+) | UCSD MS^2^ |
| 241 | | **4,4-Dimethylcholesterol** | PMI0006734 | 1253-88-9 | C29H50O | 6.30 | 397.38265 | 12.44 | 8.8 | -25.5 | 10.9 | 49.0 | 50.5 | -0.6 | 95.2 | na | | RP-APCI(+) | UCSD *in silico* MS^2^ |
| 242 | | **α-Acetylbutyrolactone** | PMI0003390 | 517-23-7 | C6H8O3 | 6.17 | 129.05463 | 1.58 | 4.4 | -4.0 | -1.3 | 48.1 | 39.5 | 2.6 | 98.9 | 0.1 | | RP-APCI(+) | UCSD MS^2^ |
| 243 | | **Butanamide, 4-cyano** | PMI0002611 | 53897-27-1 | C5H8N2O | 6.11 | 113.07122 | 1.23 | 5.8 | -11.7 | -1.4 | 42.3 | 21.2 | 4.9 | 96.0 | na | | RP-APCI(+) | UCSD *in silico* MS^2^ |
| 244 | | **5-Methoxy-3-(2-pyridinylmethyl)-1H-indole** | CSID53154 | 101832-06-8 | C15H14N2O | 5.81 | 239.11763 | 6.73 | 5.7 | -14.8 | 2.6 | 48.2 | 45.7 | -1.1 | 96.7 | na | | RP-APCI(+) | ChemIDplus *in silico* MS^2^ |
| 245 | | **2-Isobutyl-3-methoxypyrazine** | PMI0007079 | 24683-00-9 | C9H14N2O | 5.73 | 167.11778 | 4.79 | 5.5 | -1.3 | 2.6 | 43.5 | 27.9 | -0.6 | 90.2 | na | | RP-APCI(+) | UCSD *in silico* MS^2^ |
| 246 | | **Hexadecanoic acid, 16-hydroxy-** | PMI0005664 | 506-13-8 | C16H32O3 | 5.72 | 271.22837 | 8.07 | 5.7 | -18.1 | 5.1 | 51.4 | 45.0 | 1.8 | 97.3 | -0.2 | | RP-HESI(-) | UCSD MS^2^ |
| 247 | | **Propenylguaethol** | PMI0007299 | 94-86-0 | C11H14O2 | 5.70 | 161.09597 | 6.46 | 3.6 | -8.2 | 2.9 | 47.3 | 39.0 | -0.7 | 98.4 | na | | RP-APCI(+) | UCSD *in silico* MS^2^ |
| 248 | | **Stigmasteryl palmitate** | PMI0008533 | 2308-84-1 | C45H78O2 | 5.60 | 633.59673 | 17.54 | 10.4 | -39.1 | 18.5 | 39.8 | 3.0 | -0.2 | 96.5 | na | | RP-APCI(+) | UCSD *in silico* MS^2^ |
| 249 | | **1H-Benzotriazole** | PMI0003127 | 95-14-7 | C6H5N3 | 5.53 | 120.05569 | 1.48 | 2.3 | -10.6 | 1.3 | 40.1 | 7.8 | 0.6 | 93.3 | na | | RP-HESI(+) | UCSD *in silico* MS^2^ |
| 250 | | **(Z,Z)-18-hydroxy-9,12-Octadecadienoic acid** | PMI0006650 | 4546-59-2 | C18H32O3 | 5.43 | 295.22833 | 8.41 | 4.6 | -19.4 | 5.2 | 46.5 | 38.5 | 1.6 | 96.0 | na | | RP-APCI(+), **RP-HESI(-)** | UCSD *in silico* MS^2^ |
| 251 | | **9H-Carbazole, 2-amino** | PMI0004918 | 4539-51-9 | C12H10N2 | 5.39 | 181.07728 | 5.67 | 5.5 | -16.7 | 2.4 | 40.8 | 16.4 | 0.9 | 88.7 | na | | RP-HESI(-) | UCSD *in silico* MS^2^ |
| 252 | | **Hexadecanoic acid, 10,16-dihydroxy** | PMI0005722 | 3233-90-7 | C16H32O4 | 5.38 | 287.22338 | 6.40 | 4.1 | -23.4 | 3.0 | 45.7 | 34.0 | 2.1 | 97.1 | na | | RP-HESI(-) | UCSD *in silico* MS^2^ |
| 253 | | **Caryophyllene oxide** | PMI0000511 | 1139-30-6 | C15H24O | 5.35 | 203.17933 | 8.53 | 5.6 | -5.0 | 4.6 | 52.9 | 0.0 | -0.5 | 98.1 | 0.0 | | RP-APCI(+) | UCSD MS^2^ |
| 254 | | **Isonicotinamide** | PMI0003199 | 1453-82-3 | C6H6N2O | 5.16 | 123.05543 | 1.33 | 6.7 | -9.0 | -0.3 | 58.8 | 53.2 | 1.1 | 94.0 | 0.0 | | RP-APCI(+) | UCSD MS^2^ |
| 255 | | **o-Toluidin** | PMI0002779 | 95-53-4 | C7H9N | 5.15 | 108.08108 | 1.08 | 7.3 | -1.1 | 1.4 | 52.4 | 66.0 | 2.9 | 99.6 | na | | HILIC-HESI(+) | UCSD *in silico* MS^2^ |
| 256 | | **2,6-Dodecadien-1-ol, 3,7,11-trimethyl** | PMI0005409 | 7226-86-0 | C15H28O | 5.11 | 242.24737 | 9.37 | 3.1 | -9.9 | 5.8 | 40.7 | 11.2 | -2.1 | 94.6 | na | | RP-HESI(+) | UCSD *in silico* MS^2^ |
| 257 | | **2H-Pyrrol-2-one, 1,5-dihydro-3,4-dimethyl** | PMI0002871 | 4030-22-2 | C6H9NO | 5.08 | 112.07589 | 2.40 | 2.9 | -4.9 | 0.0 | 50.9 | 54.3 | 1.8 | 98.1 | -0.3 | | **RP-HESI(+)**, RP-APCI(+) | UCSD MS^2^ |
| 258 | | **5-Methyl-2-pyridinol** | PMI0002816 | 1003-68-5 | C6H7NO | 5.05 | 110.06039 | 2.07 | 4.9 | -7.0 | 0.0 | 54.9 | 69.6 | 3.2 | 97.4 | -0.1 | | RP-APCI(+) | UCSD MS^2^ |
| 259 | | **N~5~-(Diaminomethylene)-N~2~-(2,3-dihydroxybenzoyl)-L-ornithyl-L-threonine** | CSID170867 | 143651-45-0 | C17H25N5O7 | 4.91 | 429.20929 | 3.12 | 5.0 | 0.0 | -0.3 | 39.5 | 0.0 | 0.2 | 98.0 | na | | RP-HESI(+) | ChemIDplus *in silico* MS^2^ |
| 260 | | **Hydroxycotinine** | PMI0006903 | 34834-67-8 | C10H12N2O2 | 4.87 | 193.09692 | 1.91 | 2.5 | -16.0 | -1.5 | 53.6 | 48.0 | -1.2 | 93.3 | 0.0 | | **RP-HESI(+)**, RP-APCI(+) | UCSD MS^2^ |
| 261 | | **γ-Nonalactone** | PMI0004394 | 104-61-0 | C9H16O2 | 4.86 | 139.11164 | 5.80 | 4.7 | -4.8 | 1.9 | 57.0 | 81.9 | -0.7 | 98.9 | -0.5 | | RP-APCI(+) | UCSD MS^2^ |
| 262 | | **Vitamin K1** | PMI0007593 | 84-80-0 | C31H46O2 | 4.82 | 451.35694 | 14.67 | 7.6 | -26.0 | 12.3 | 64.6 | 98.6 | -0.3 | 96.2 | 0.1 | | RP-HESI(+), **RP-APCI(+)** | UCSD MS^2^ |
| 263 | | **1-keto-α-Cyperone** | PMI0005461 | 38043-97-9 | C15H20O2 | 4.78 | 233.15342 | 7.50 | 5.5 | -13.6 | 4.1 | 49.5 | 51.9 | -0.8 | 96.8 | na | | RP-HESI(+), **RP-APCI(+)** | UCSD *in silico* MS^2^ |
| 264 | | **2H-Pyrrol-2-one, 3-ethyl-1,5-dihydro-4-methyl** | PMI0003313 | 766-36-9 | C7H11NO | 4.75 | 126.09142 | 3.80 | 4.4 | -5.5 | 0.5 | 47.8 | 40.8 | 0.7 | 98.9 | na | | **RP-APCI(+)**, HILIC-HESI(+) | UCSD *in silico* MS^2^ |
| 265 | | **Ethanone, 1-(3,4-dihydro-4-methylpyrazin-2-yl)** | PMI0003817 | na | C7H10N2O | 4.72 | 139.08648 | 3.00 | 5.5 | -2.2 | -0.8 | 42.2 | 20.2 | -0.8 | 91.9 | na | | RP-APCI(+) | UCSD *in silico* MS^2^ |
| 266 | | **Pyrazine, dimethyl** | PMI0000239 | 5910-89-4 | C6H8N2 | 4.70 | 109.07628 | 3.07 | 6.4 | 1.2 | 0.6 | 52.7 | 63.7 | 2.4 | 93.2 | -0.1 | | **RP-HESI(+)**, RP-APCI(+), HILIC-HESI(+) | UCSD MS^2^ |
| 267 | | **Tyrosine** | PMI0004882 | 55520-40-6 | C9H11NO3 | 4.64 | 164.07039 | 2.44 | 3.0 | -13.6 | 0.4 | 46.6 | 35.4 | -1.2 | 98.9 | na | | RP-APCI(+) | UCSD *in silico* MS^2^ |
| 268 | | **2-Ethyl-3-pyridinol** | PMI0003065 | 61893-02-5 | C7H9NO | 4.55 | 124.07576 | 1.17 | 2.9 | -6.4 | 1.6 | 53.2 | 53.5 | 0.5 | 98.7 | 0.0 | | HILIC-HESI(+) | UCSD MS^2^ |
| 269 | | **m-Acetylphenol** | PMI0000285 | 121-71-1 | C8H8O2 | 4.31 | 137.05946 | 3.42 | 3.3 | -5.9 | 1.4 | 52.6 | 63.9 | -1.8 | 96.2 | -0.3 | | RP-HESI(+) | UCSD MS^2^ |
| 270 | | **Nonadecanoic acid** | PMI0005752 | 646-30-0 | C19H38O2 | 4.28 | 297.28038 | 11.02 | 5.7 | -12.3 | 8.7 | 59.2 | 78.1 | 1.6 | 97.0 | -0.2 | | RP-HESI(-) | UCSD MS^2^ |
| 271 | | **Farnesylacetic acid** | PMI0011818 | 6040-06-8 | C17H28O2 | 4.28 | 263.20208 | 8.80 | 4.8 | -15.1 | 6.1 | 53.0 | 34.3 | 1.6 | 97.1 | 0.1 | | RP-HESI(-) | UCSD MS^2^ |
| 272 | | **(2E)-3-(3-Methylphenyl)acrylic acid** | PMI0011803 | 14473-89-3 | C10H10O2 | 4.26 | 161.06068 | 4.52 | 4.0 | -7.5 | 2.9 | 45.3 | 28.4 | -0.8 | 99.2 | na | | RP-HESI(-) | UCSD *in silico* MS^2^ |
| 273 | | **17-Hydroxylinolenic acid** | PMI0011840 | na | C18H30O3 | 4.23 | 277.21601 | 8.66 | 7.4 | -19.4 | 4.7 | 45.3 | 30.7 | -0.7 | 96.8 | na | | RP-APCI(+) | UCSD *in silico* MS^2^ |
| 274 | | **4-Ethylcatechol** | PMI0000305 | 1124-39-6 | C8H10O2 | 4.16 | 139.07512 | 4.39 | 2.3 | -5.7 | 1.9 | 54.9 | 68.9 | -1.7 | 97.4 | 0.1 | | RP-HESI(+) | UCSD MS^2^ |
| 275 | | **Strychnocarpine** | PMI0011889 | 59156-98-8 | C12H12N2O | 4.09 | 199.08799 | 6.16 | 5.7 | -16.3 | 0.6 | 41.1 | 14.7 | 1.5 | 87.2 | 0.4 | | RP-HESI(-) | UCSD MS^2^ |
| 276 | | **1-p-Tolylcyclopropanecarboxylic acid** | PMI0011808 | 83846-66-6 | C11H12O2 | 4.03 | 175.07652 | 5.35 | 3.1 | -9.3 | 2.0 | 53.8 | 25.6 | 0.4 | 98.6 | 0.0 | | RP-HESI(-) | UCSD MS^2^ |
| 277 | | **[1-Methyl-3-oxo-2-pentylidenecyclopentyl]acetic acid** | PMI0011854 | 958790-53-9 | C13H20O3 | 3.97 | 223.13427 | 4.06 | 3.7 | -14.0 | 2.4 | 50.9 | 58.3 | 1.3 | 97.7 | na | | RP-HESI(-) | UCSD *in silico* MS^2^ |
| 278 | | **Phenylacetic acid** | PMI0000156 | 103-82-2 | C8H8O2 | 3.94 | 135.04467 | 4.49 | 5.0 | -5.4 | 1.5 | 44.4 | 27.9 | -3.5 | 98.4 | na | | RP-HESI(-) | UCSD *in silico* MS^2^ |
| 279 | | **Hexadecanoic acid, 2-hydroxy-1(hydroxymethyl)ethyl ester** | PMI0007519 | 23470-00-0 | C19H38O4 | 3.94 | 313.27339 | 15.41 | 7.1 | -22.3 | 6.4 | 40.2 | 6.0 | -1.0 | 96.3 | na | | RP-APCI(+) | UCSD *in silico* MS^2^ |
| 280 | | **5-(2-Aminoethyl)guaiacol** | CSID1685 | na | C9H13NO2 | 3.87 | 150.09124 | 3.72 | 5.1 | -7.8 | 0.4 | 46.0 | 33.4 | -0.7 | 97.3 | na | | RP-APCI(+) | FDA *in silico* MS^2^ |
| 281 | | **Cyclohexylphenylacetic acid** | PMI0011801 | 3894-09-5 | C14H18O2 | 3.68 | 217.12366 | 6.93 | 3.4 | -11.2 | 3.9 | 50.3 | 13.5 | 1.2 | 96.8 | 0.0 | | RP-HESI(-) | UCSD MS^2^ |
| 282 | | **5-Oxo-1-tetradecyl-3-pyrrolidinecarboxylic acid** | PMI0011834 | 10054-22-5 | C19H35NO3 | 3.65 | 326.26870 | 9.29 | 7.8 | -23.2 | 5.8 | 43.4 | 21.7 | -0.8 | 96.2 | na | | **RP-APCI(+),** RP-HESI(-) | UCSD *in silico* MS^2^ |
| 283 | | **N-Nonylimidazole** | PMI0011878 | 53657-08-2 | C12H22N2 | 3.54 | 195.18548 | 3.56 | 2.7 | -7.3 | 4.0 | 46.7 | 46.8 | -0.5 | 87.3 | na | | HILIC-HESI(+) | UCSD *in silico* MS^2^ |
| 284 | | **3-Amino-2-piperidone** | HMDB00323 | 1892-22-4 | C5H10N2O | 3.41 | 97.07654 | 1.18 | 6.0 | -7.2 | -2.2 | 46.7 | 40.2 | 4.5 | 98.5 | na | | RP-APCI(+) | HMDB *in silico* MS^2^ |
| 285 | | **2H-Pyran-2-one, 3-hydroxy-6-methyl** | PMI0003324 | 73692-69-0 | C6H6O3 | 3.37 | 127.03904 | 3.79 | 10.2 | -7.9 | 0.2 | 40.5 | 5.1 | 0.6 | 98.2 | na | | RP-APCI(+) | UCSD *in silico* MS^2^ |
| 286 | | **(4-Methyl-1-phenyl-1H-pyrrolo[2,3-b]pyridin-3-yl)methanol** | CSID2303055 | 50839-70-8 | C15H14N2O | 3.31 | 239.11765 | 5.65 | 5.9 | -17.9 | 2.7 | 49.9 | 54.5 | -1.0 | 96.0 | na | | RP-APCI(+) | ChemIDplus *in silico* MS^2^ |
| 287 | | **Verbenol** | PMI0010059 | 18881-04-4 | C10H16O | 3.29 | 153.12728 | 6.56 | 4.4 | -3.4 | 2.5 | 53.7 | 63.8 | -0.8 | 98.2 | -0.3 | | RP-APCI(+) | UCSD MS^2^ |
| 288 | | **Amyl cinnamyl alcohol** | PMI0009335 | 101-85-9 | C14H20O | 3.24 | 205.15857 | 7.51 | 4.7 | -9.7 | 4.4 | 47.2 | 25.6 | -0.7 | 97.1 | 0.2 | | RP-APCI(+) | UCSD MS^2^ |
| 289 | | **2-Vinylpyridine** | PMI0000241 | 100-69-6 | C7H7N | 3.09 | 106.06550 | 4.60 | 5.7 | 1.1 | 1.3 | 55.0 | 45.2 | 3.6 | 92.4 | 0.0 | | RP-APCI(+) | UCSD MS^2^ |
| 290 | | **Cyclo (Ile-Val)** | PMI0008777 | na | C11H20N2O2 | 2.93 | 195.14910 | 6.58 | 5.1 | -16.3 | 0.7 | 48.0 | 42.4 | -0.4 | 98.0 | na | | RP-APCI(+) | UCSD *in silico* MS^2^ |
| 291 | | **Glyceryl palmitate** | PMI0000383 | 542-44-9 | C19H38O4 | 2.90 | 313.27338 | 14.48 | 6.3 | -21.5 | 6.2 | 39.1 | 0.2 | -1.0 | 96.6 | na | | RP-APCI(+) | NIST MS/MS |
| 292 | | **α-Ionol** | PMI0000344 | 25312-34-9 | C13H22O | 2.89 | 177.16364 | 8.17 | 7.0 | -6.6 | 4.3 | 63.5 | 89.1 | -0.7 | 98.0 | -0.1 | | RP-APCI(+) | UCSD MS^2^ |
| 293 | | **Oxazole, 4-pentyl** | PMI0003483 | na | C8H13NO | 2.85 | 140.10688 | 4.56 | 5.1 | -0.4 | 2.7 | 44.1 | 22.7 | -0.8 | 98.6 | na | | RP-APCI(+) | UCSD *in silico* MS^2^ |
| 294 | | **Stigmasta-5,7,22,25-tetraen-3-ol** | PMI0011883 | 119386-11-7 | C29H44O | 2.84 | 409.34632 | 13.16 | 7.2 | -28.1 | 9.5 | 51.9 | 65.0 | -0.4 | 94.9 | na | | RP-HESI(+), **RP-APCI(+)** | UCSD *in silico* MS^2^ |
| 295 | | **Cyclo (Pro-Tyr)** | PMI0008615 | 4549-02-4 | C14H16N2O3 | 2.81 | 261.12317 | 2.43 | 5.7 | -30.8 | -1.3 | 53.1 | 68.1 | -0.8 | 98.0 | na | | RP-APCI(+) | UCSD *in silico* MS^2^ |
| 296 | | **3-Acetylaniline** | PMI0011382 | 99-03-6 | C8H9NO | 2.77 | 136.07563 | 3.09 | 7.0 | -6.2 | 0.7 | 50.5 | 47.0 | -0.5 | 98.3 | -0.2 | | RP-APCI(+) | UCSD MS^2^ |
| 297 | | **1H-Indole-3-ethanol** | PMI0004297 | 526-55-6 | C10H11NO | 2.69 | 144.08071 | 4.59 | 6.7 | -11.5 | 1.3 | 65.7 | 72.8 | -0.4 | 98.3 | 0.0 | | RP-APCI(+) | UCSD MS^2^ |
| 298 | | **1-(2-Ethyloctahydro-1H-isoindol-1-yl)methanamine** | CSID83484 | 56625-54-8 | C11H22N2 | 2.67 | 183.18536 | 4.93 | 4.0 | -2.9 | 1.9 | 46.2 | 43.9 | -1.2 | 88.4 | na | | HILIC-HESI(+) | ChemIDplus *in silico* MS^2^ |
| 299 | | **2,3-Dimethylindole** | PMI0000262 | 91-55-4 | C10H11N | 2.62 | 146.09634 | 6.64 | 4.0 | -5.2 | 3.1 | 54.6 | 63.1 | -0.6 | 98.3 | -0.2 | | RP-APCI(+) | UCSD MS^2^ |
| 300 | | **2-Pyridinamine, 5-methyl** | PMI0002787 | 1603-41-4 | C6H8N2 | 2.57 | 109.07635 | 2.01 | 5.2 | -4.5 | -0.1 | 50.5 | 62.4 | 3.0 | 93.6 | na | | HILIC-HESI(+) | UCSD *in silico* MS^2^ |
| 301 | | **1H-Imidazole, butyl** | PMI0003282 | 50790-93-7 | C7H12N2 | 2.45 | 125.10740 | 3.14 | 1.6 | -5.0 | 1.2 | 50.7 | 37.2 | 0.6 | 98.7 | 0.1 | | HILIC-HESI(+) | UCSD MS^2^ |
| 302 | | **1H-Indol-5-ol** | PMI0011367 | 1953-54-4 | C8H7NO | 2.44 | 134.05989 | 2.33 | 3.0 | -10.2 | 1.0 | 63.2 | 43.0 | -1.1 | 97.0 | 0.0 | | RP-HESI(+) | UCSD MS^2^ |
| 303 | | **Methyl nicotinate** | PMI0000773 | 93-60-7 | C7H7NO2 | 2.30 | 155.08128 | 1.62 | 2.2 | -1.6 | 0.9 | 44.5 | 32.4 | -1.6 | 92.2 | na | | HILIC-HESI(+) | UCSD *in silico* MS^2^ |
| 304 | | **Cinnamic acid** | PMI0008604 | 621-82-9 | C9H8O2 | 2.28 | 147.04483 | 3.54 | 4.2 | -5.4 | 2.4 | 47.5 | 46.2 | -2.2 | 90.4 | 0.4 | | RP-HESI(+), **RP-HESI(-)**, HILIC-HESI(+) | UCSD MS^2^ |
| 305 | | **Benzeneacetonitrile, 4-hydroxy** | PMI0003605 | 14191-95-8 | C8H7NO | 2.27 | 134.06004 | 3.24 | 5.7 | -9.3 | 0.7 | 47.0 | 36.0 | 0.0 | 98.8 | na | | RP-APCI(+) | UCSD *in silico* MS^2^ |
| 306 | | **Cyclo (Leu-Val)** | PMI0008786 | 5625-50-3 | C11H20N2O2 | 2.27 | 195.14909 | 6.67 | 4.9 | -16.3 | 0.7 | 47.1 | 39.1 | -0.5 | 97.1 | na | | RP-APCI(+) | UCSD *in silico* MS^2^ |
| 307 | | **1,2-Benzenediamine, 4,5-dimethyl** | PMI0003754 | 3171-45-7 | C8H12N2 | 2.21 | 137.10723 | 2.78 | 2.3 | -5.5 | 1.0 | 49.5 | 50.2 | -0.7 | 98.2 | na | | HILIC-HESI(+) | UCSD *in silico* MS^2^ |
| 308 | | **1-(4-Methoxyphenyl)-5-methyl-1H-pyrrole-2-acetic acid** | CSID2305866 | 60352-53-6 | C14H15NO3 | 2.13 | 244.09831 | 7.28 | 6.7 | -16.3 | 1.4 | 43.0 | 19.9 | 1.6 | 97.2 | na | | RP-HESI(-) | ChemIDplus *in silico* MS^2^ |
| 309 | | **Jasmolone** | PMI0011865 | 54383-66-3 | C11H16O2 | 1.98 | 181.12214 | 5.86 | 5.6 | -9.7 | 1.5 | 49.9 | 52.4 | -0.9 | 98.3 | na | | RP-APCI(+) | UCSD *in silico* MS^2^ |
| 310 | | **1-Cyclohexylpiperazine** | PMI0011807 | 17766-28-8 | C10H20N2 | 1.97 | 169.16973 | 5.14 | 4.2 | -4.1 | 1.9 | 43.7 | 30.6 | -1.2 | 89.5 | na | | HILIC-HESI(+) | UCSD *in silico* MS^2^ |
| 311 | | **Squalene** | PMI0000029 | 111-02-4 | C30H50 | 1.94 | 411.39840 | 15.50 | 6.8 | -14.8 | 13.1 | 55.7 | 66.4 | -0.3 | 95.2 | 0.3 | | RP-APCI(+) | UCSD MS^2^ |
| 312 | | **1-(5,6,7,8-Tetrahydro-1-naphthalenyl)piperazine** | CSID89139 | 57536-84-2 | C14H20N2 | 1.94 | 217.16971 | 4.94 | 3.7 | -13.3 | 2.8 | 44.0 | 23.4 | -1.0 | 98.0 | na | | HILIC-HESI(+) | ChemIDplus *in silico* MS^2^ |
| 313 | | **(1S,3S)-1-Ethyl-2,3,4,9-tetrahydro-1H-beta-carboline-3-carboxylic acid** | CSID155141 | 134930-19-1 | C14H16N2O2 | 1.88 | 243.11419 | 5.71 | 3.8 | -22.5 | 1.8 | 40.7 | 32.0 | 1.2 | 73.1 | na | | RP-HESI(-) | ChemIDplus *in silico* MS^2^ |
| 314 | | **Cyclo (Ile-Ile)** | PMI0008771 | na | C12H22N2O2 | 1.72 | 225.16121 | 5.87 | 5.0 | -17.0 | 1.2 | 39.4 | 1.6 | 1.6 | 97.2 | na | | RP-HESI(-) | UCSD *in silico* MS^2^ |
| 315 | | **picolinamide** | PMI0000185 | 1452-77-3 | C6H6N2O | 1.71 | 123.05545 | 2.14 | 3.7 | -9.0 | 0.0 | 53.4 | 62.8 | 1.3 | 94.7 | -0.1 | | RP-APCI(+) | UCSD MS^2^ |
| 316 | | **1-Benzyl-3-piperidinone hydrochloride** | PMI0011787 | 40114-49-6 | C12H15NO | 1.69 | 207.14903 | 2.95 | 3.9 | -6.4 | 1.2 | 46.9 | 39.8 | -0.8 | 95.6 | na | | HILIC-HESI(+) | UCSD *in silico* MS^2^ |
| 317 | | **2,3-Diethylpyrazine** | PMI0003748 | 15707-24-1 | C8H12N2 | 1.65 | 137.10720 | 5.17 | 4.1 | 0.0 | 1.7 | 51.2 | 40.2 | -0.9 | 91.4 | 0.1 | | HILIC-HESI(+) | UCSD MS^2^ |
| 318 | | **1H-Pyrrolo[2,3-b]pyridine, 2-methyl** | PMI0003586 | 23612-48-8 | C8H8N2 | 1.51 | 133.07598 | 2.21 | 6.0 | -5.2 | 2.3 | 43.3 | 25.3 | -0.3 | 91.5 | na | | HILIC-HESI(+) | UCSD *in silico* MS^2^ |
| 319 | | **2,3'-Bipyrrolidine** | PMI0004687 | 38602-84-5 | C8H16N2 | 1.40 | 141.13844 | 5.63 | 2.4 | -2.0 | 0.2 | 47.0 | 45.0 | -1.3 | 91.4 | na | | HILIC-HESI(+) | UCSD *in silico* MS^2^ |
| 320 | | **1H-Imidazole, 2-methyl-4-(1-methylethyl)** | PMI0003280 | 37455-52-0 | C7H12N2 | 1.39 | 125.10737 | 5.75 | 4.2 | -4.3 | 1.0 | 47.0 | 37.7 | 0.4 | 97.7 | na | | HILIC-HESI(+) | UCSD *in silico* MS^2^ |
| 321 | | **13'-Hydroxy-gamma-tocopherol** | HMDB12561 | na | C28H48O3 | 1.29 | 415.35689 | 12.82 | 9.9 | -28.4 | 9.4 | 51.8 | 65.1 | -0.4 | 94.4 | na | | RP-APCI(+) | HMDB *in silico* MS^2^ |
| 322 | | **2-(2-Isobutyl-3-furyl)propanoic acid** | CSID2289407 | na | C11H16O3 | 1.28 | 195.10290 | 4.26 | 6.5 | -6.4 | 2.9 | 48.5 | 45.7 | 1.2 | 98.1 | na | | RP-HESI(-) | ChemIDplus *in silico* MS^2^ |
| 323 | | **3-Methyl-pyridazine** | PMI0000242 | 1632-76-4 | C5H6N2 | 1.06 | 95.06083 | 1.57 | 3.0 | -1.4 | -0.3 | 62.8 | 76.9 | 4.9 | 96.7 | 0.0 | | RP-HESI(+) | UCSD MS^2^ |
| 324 | | **9-Methyladenine** | PMI0011802 | 700-00-5 | C6H7N5 | 1.02 | 150.07734 | 3.26 | 2.0 | -12.5 | -0.3 | 54.3 | 69.9 | -0.5 | 92.5 | 0.1 | | HILIC-HESI(+) | UCSD MS^2^ |
| 325 | | **2-Methoxy-6-(2-propenyl)phenol** | PMI0010195 | 579-60-2 | C10H12O2 | 0.96 | 182.11737 | 5.44 | 3.4 | -4.4 | 2.2 | 42.5 | 15.2 | -1.1 | 98.7 | na | | HILIC-HESI(+) | UCSD *in silico* MS^2^ |
| 326 | | **4-Imidazolemethanol** | PMI0009960 | 822-55-9 | C4H6N2O | 0.85 | 99.05579 | 3.51 | 4.9 | -14.2 | -1.3 | 47.2 | 46.3 | 5.1 | 95.5 | na | | HILIC-HESI(+) | UCSD *in silico* MS^2^ |
| 327 | | **Diacetin** | PMI0000341 | 102-62-5 | C7H12O5 | 0.85 | 159.06466 | 2.32 | 9.9 | -5.0 | -0.5 | 60.5 | 25.3 | -3.0 | 98.3 | 0.0 | | RP-APCI(+) | UCSD MS^2^ |
| 328 | | **3-Ethylpyridine** | PMI0000240 | 536-78-7 | C7H9N | 0.78 | 108.08113 | 4.53 | 8.7 | 0.9 | 1.7 | 53.7 | 62.1 | 3.3 | 98.4 | 0.1 | | RP-APCI(+) | UCSD MS^2^ |
| 329 | | **3,5-Diethyl-2-methylpyrazine** | PMI0007257 | 18138-05-1 | C9H14N2 | 0.42 | 151.12282 | 5.50 | 3.5 | -1.0 | 2.2 | 47.1 | 46.5 | -1.0 | 90.5 | na | | HILIC-HESI(+) | UCSD *in silico* MS^2^ |
| 330 | | **4,5-Dicyanoimidazole** | CSID63894 | 1122-28-7 | C5H2N4 | 0.29 | 119.03528 | 1.18 | 3.8 | -28.2 | 0.1 | 42.3 | 18.9 | 0.0 | 93.3 | na | | RP-HESI(+) | ChemIDplus *in silico* MS^2^ |
| 331 | | **2(3H)-Furanone, dihydro-5-(1-hydroxyethyl)** | PMI0003461 | 27610-27-1 | C6H10O3 | 0.19 | 131.07046 | 1.47 | 5.7 | -8.4 | -1.5 | 53.5 | 58.1 | 1.4 | 98.0 | 0.0 | | RP-HESI(+) | UCSD MS^2^ |
|  | | | | | | | | | | | | | | | |  |  |  |  |

**Table S2 Subset of compounds in 3R4F-derived smoke identified with UCSD MS^2^ and NIST MS/MS libraries.** All compounds were confirmed with reference standards. The serial numbers (#) correspond to those in Table S1, which contains further information on the compounds

| **#** | **Name** | **Identifier** | **CAS** | **UCSD MS^2^ Frag. Score** | **NIST MS/MS Frag. Score** |
| --- | --- | --- | --- | --- | --- |
| 2 | Nicotine | PMI0004286 | 22083-74-5 | 98.9 | 97.7 |
| 4 | Triacetin | PMI0000113 | 102-76-1 | 100.0 | 0.0 |
| 15 | Linolic acid | PMI0000168 | 60-33-3 | 64.0 | 0.0 |
| 40 | Norharman | PMI0000439 | 244-63-3 | 63.9 | 16.9 |
| 72 | Harman | PMI0000269 | 486-84-0 | 57.0 | 36.6 |
| 92 | 3-Pyridinol | PMI0000271 | 109-00-2 | 77.2 | 30.7 |
| 100 | α-Tocopherol | PMI0006937 | 59-02-9 | 91.3 | 87.7 |
| 146 | Moroctic acid | PMI0011796 | 20290-75-9 | 50.5 | 50.2 |
| 164 | 2-Methyl-5-pyridinol | PMI0002804 | 1121-78-4 | 48.9 | 25.2 |
| 182 | Cerotinic acid | PMI0005981 | 506-46-7 | 5.2 | 0.0 |
| 197 | Catechol | PMI0000457 | 120-80-9 | 86.1 | 0.9 |
| 210 | Guaiacol, 5-amino- | PMI0011719 | 1687-53-2 | 31.1 | 11.9 |
| 231 | Harmol | PMI0011375 | 487-03-6 | 35.1 | 59.7 |
| 232 | Retinol | PMI0007452 | 68-26-8 | 64.1 | 1.6 |
| 234 | Ricinoleic acid | PMI0011792 | 141-22-0 | 29.1 | 27.7 |
| 297 | 1H-Indole-3-ethanol | PMI0004297 | 526-55-6 | 72.8 | 14.5 |

**Figure S1 Identified compounds of tobacco smoke by use of the four separate chromatographic/ionization approaches in LC-HRAM-MS-based NTS.** LogP_OW_, log-transformed octanol/water partition coefficient calculated by ACD/Labs Percepta software suite

**Figure S2 Differentiation of structural isomers in tobacco smoke using LC-HRAM-MS and an experimental MS^2^ fragmentation database.** The NTS workflow allows discrimination of compounds with same accurate mass and isotopic distribution, as illustrated for cotinine (upper panel) and N-formylnornicotine (lower panel) due to their different fragmentation spectra

| **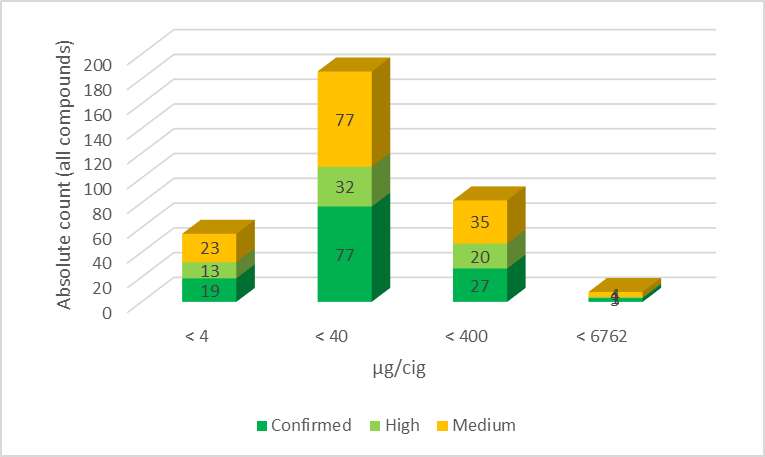** | **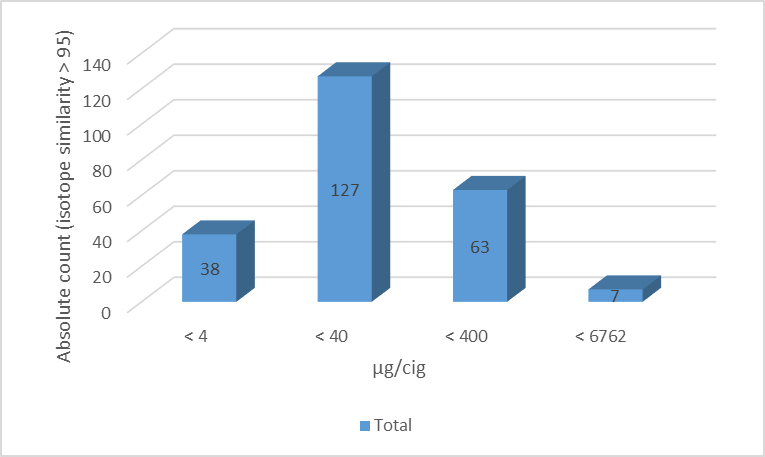** |
| --- | --- |
| **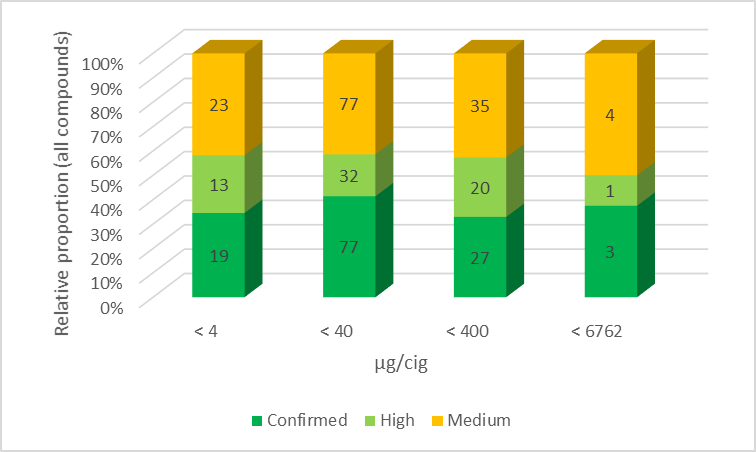** | **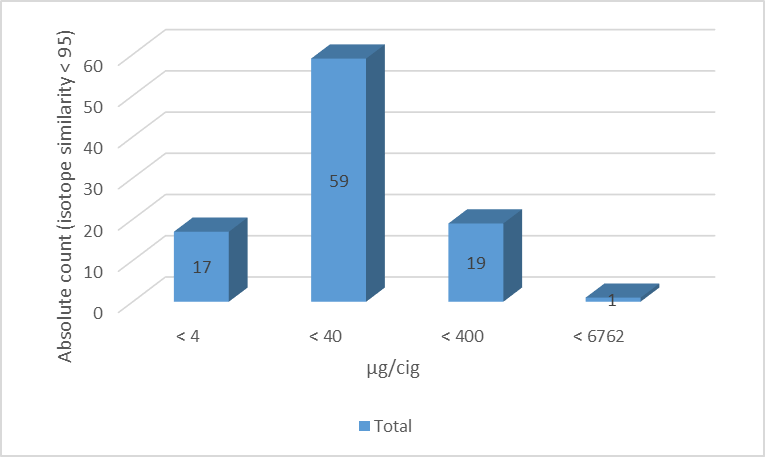** |

**Figure S3 Distribution of groups of compounds with different identification confidence and isotope similarity within the concentration range.** Confidence levels: dark green, confirmed: tR and mass spectra were within specified tolerance ranges in comparison to a standard under the same experimental conditions; light green, high: overall score > 50 or overall score > 45 and FS > 45; yellow, medium: overall score < 45 or overall score between 45 and 50 and FS < 45. Values displayed in the bar charts always represent absolute compound numbers

**
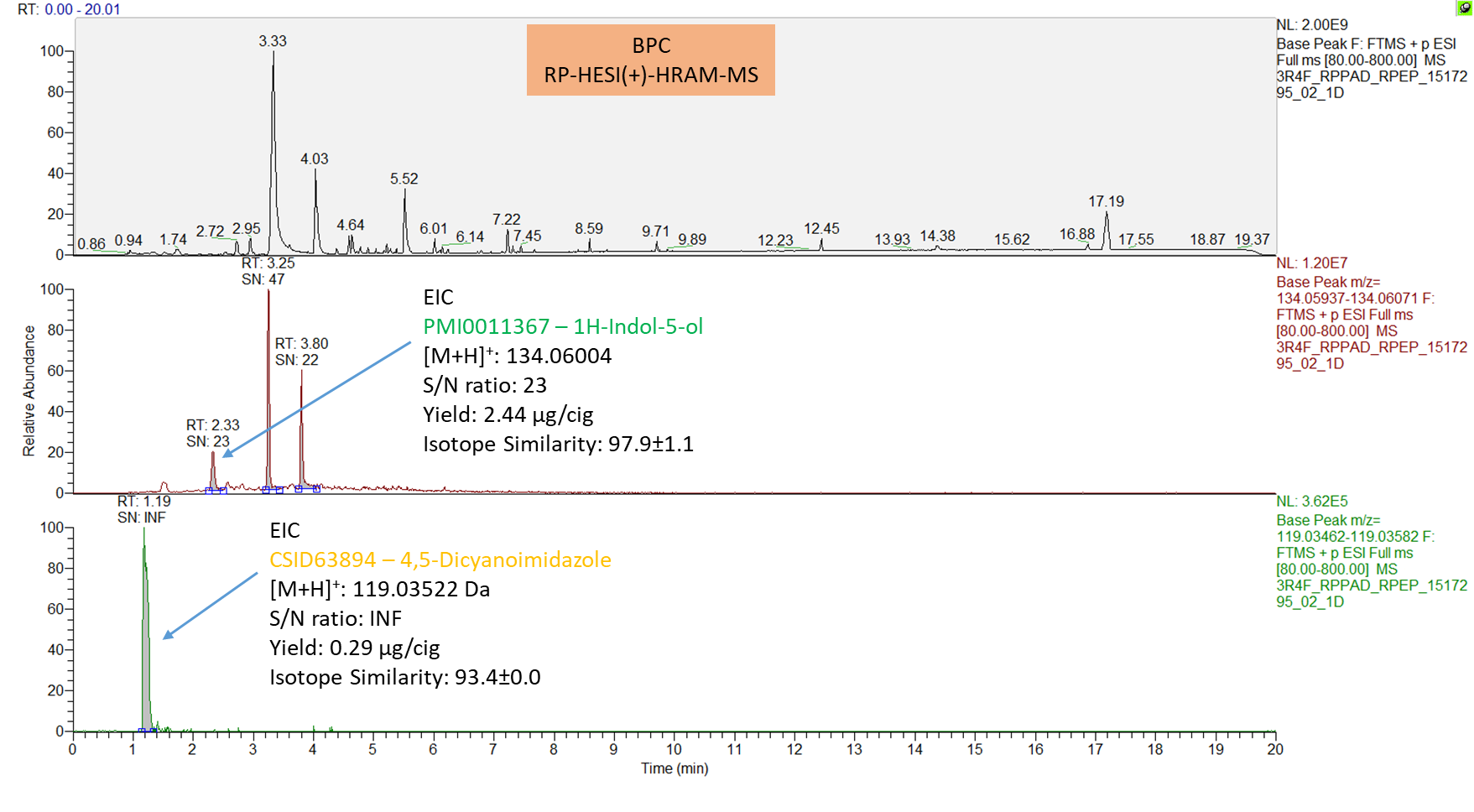
**

**
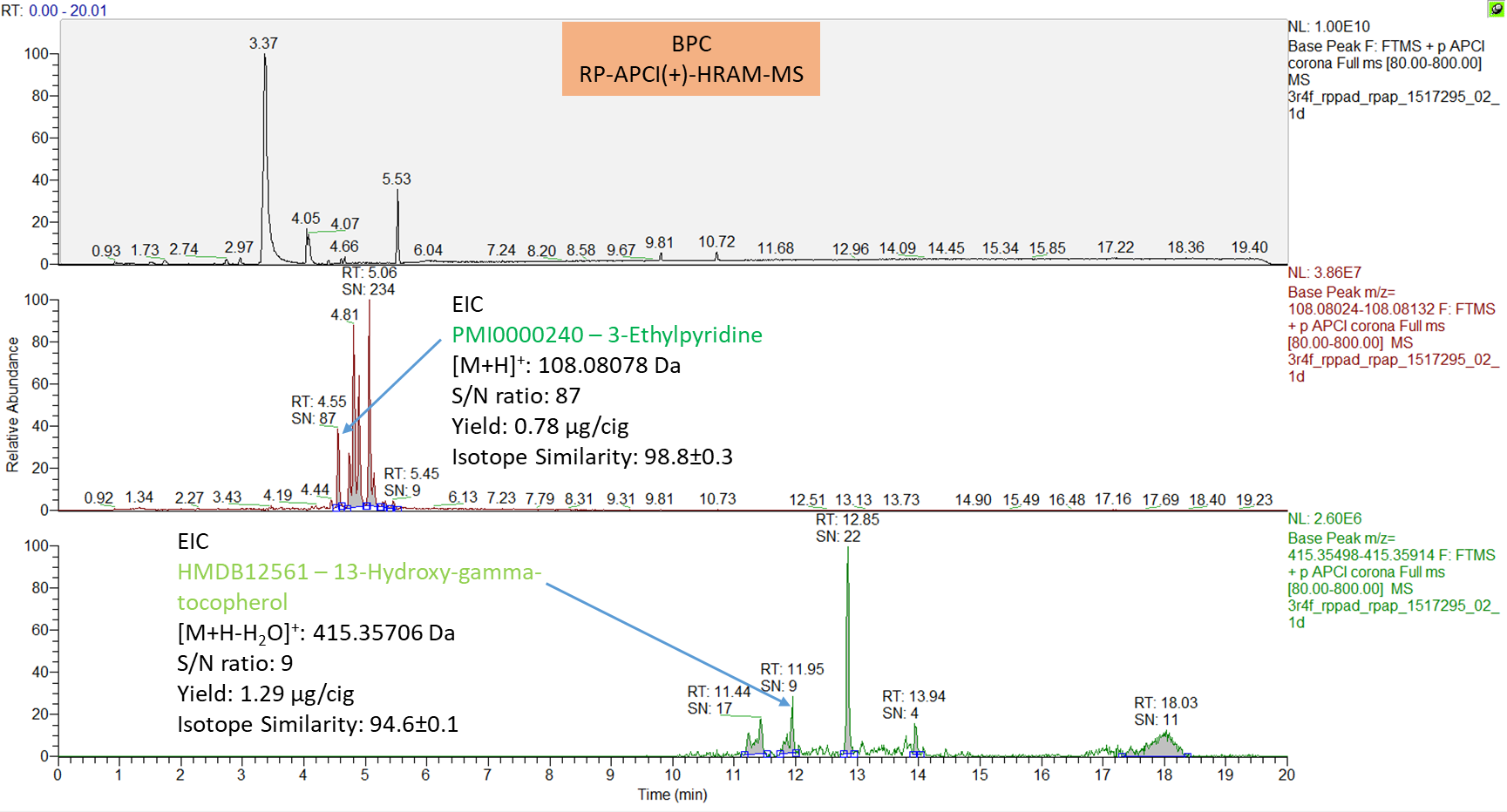
**

**Figure S4 Base peak and extracted ion chromatograms for two compounds of low concentration for each of the four analytical methods.** Confidence levels: dark green, confirmed: tR and mass spectra were within specified tolerance ranges in comparison to a standard under the same experimental conditions; light green, high: overall score > 50 or overall score > 45 and FS > 45; yellow, medium: overall score < 45 or overall score between 45 and 50 and FS < 45. Isotope similarity values are given as mean±SD calculated from three sample preparation replicates

**
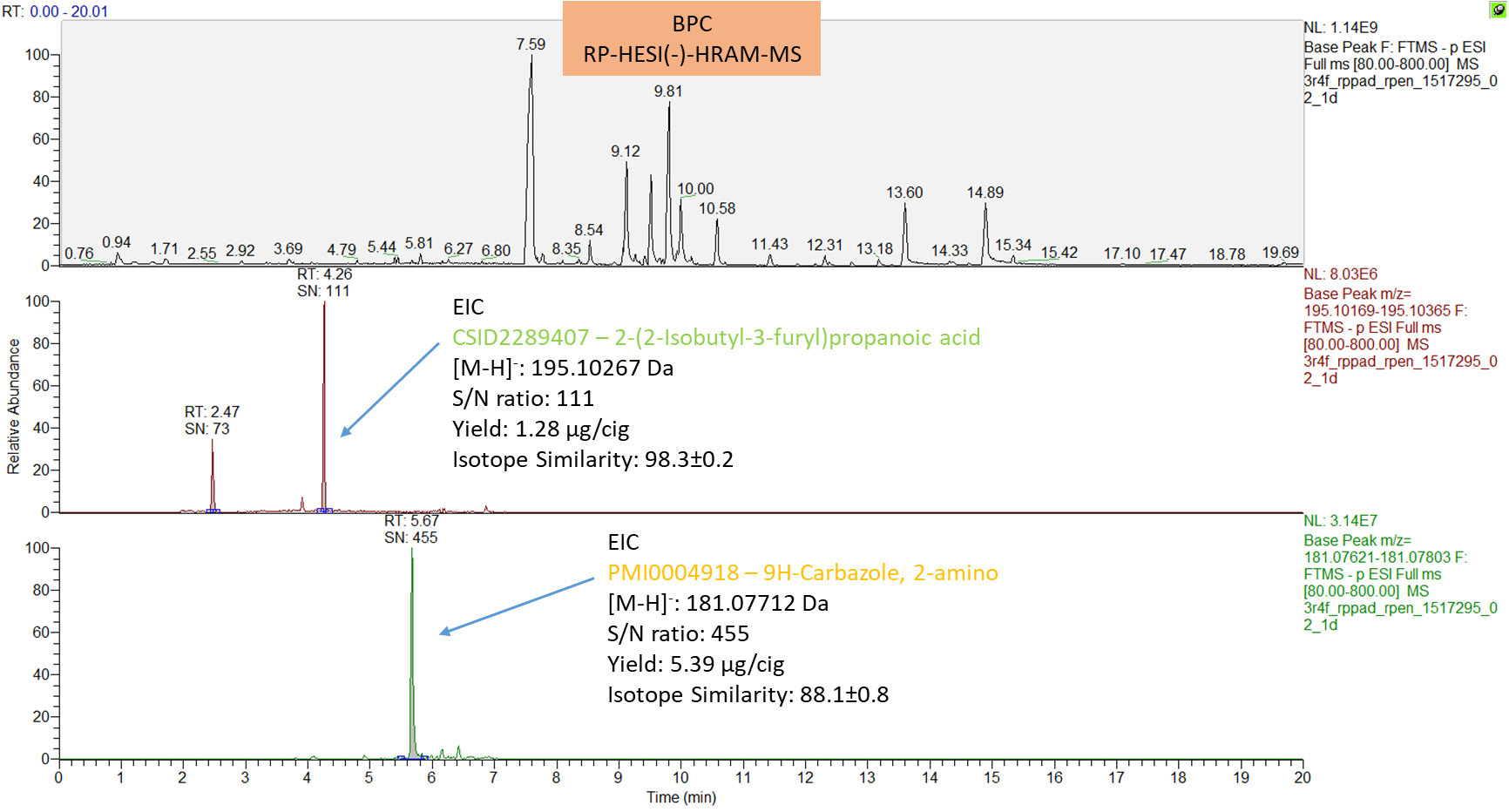
**

**
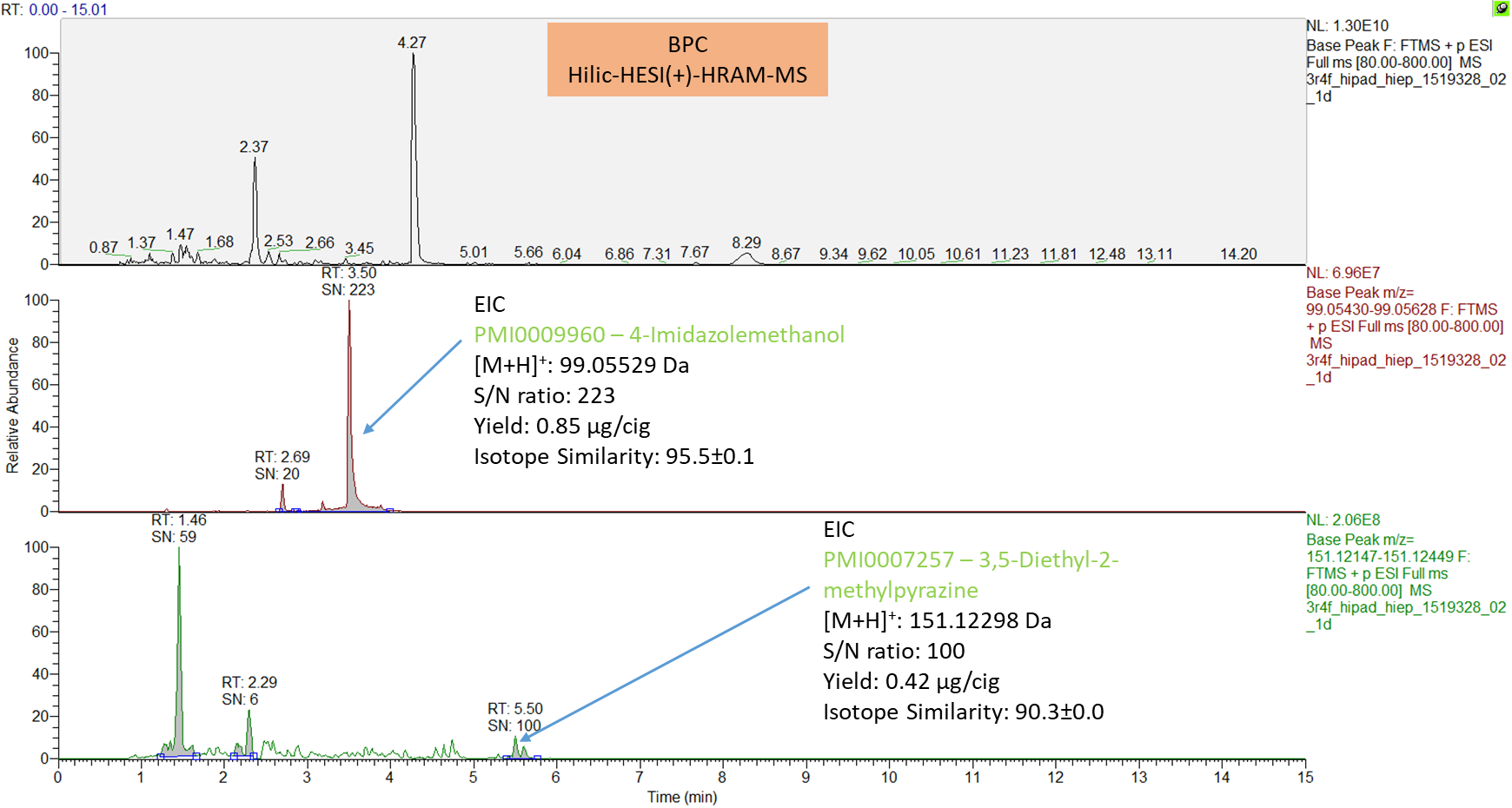
**

**Figure S4 continued.**
